# INTERNATIONAL KIDNEY CANCER SYMPOSIUM: EUROPE

**DOI:** 10.15586/jkcvhl.v13iSP2.480

**Published:** 2026-04-16

**AUTHORS**

Cecilie Lindgaard, Department of Molecular Medicine, Aarhus University Hospital, Aarhus, Denmark and Department of Clinical Medicine, Aarhus University, Aarhus, Denmark

Jesper Jespersen, Department of Molecular Medicine, Aarhus University Hospital, Aarhus, Denmark and Department of Clinical Medicine, Aarhus University, Aarhus, Denmark

Mads Holm Christensen, Department of Immunology and Microbiology, University of Copenhagen, Copenhagen, Denmark and Department of Clinical Microbiology, Rigshospitalet, Copenhagen, Denmark

Arnon Møldrup Knudsen, Aarhus University Hospital, Aarhus, Denmark

Niels Fristrup, Department of Clinical Medicine, Aarhus University, Aarhus, Denmark and Department of Oncology, Aarhus University Hospital, Aarhus, Denmark

Anna Krarup Keller, Department of Clinical Medicine, Aarhus University, Aarhus, Denmark and Department of Urology, Aarhus University Hospital, Aarhus, Denmark

Thomas Bjarnsholt, Department of Immunology and Microbiology, University of Copenhagen, Copenhagen, Denmark and Department of Clinical Microbiology, Rigshospitalet, Copenhagen, Denmark

Iben Lyskjær, Department of Molecular Medicine, Aarhus University Hospital, Aarhus, Denmark and Department of Clinical Medicine, Aarhus University, Aarhus, Denmark

Background: Clear cell renal cell carcinoma (ccRCC) remains a clinical challenge. Recent evidence from other solid tumors suggests that intratumoral bacteria can influence anti-tumor immunity and treatment response. However, the presence of bacteria within ccRCC tumor tissue remains debated, and direct tissue-based visual evidence is lacking.

Methods: We performed 16S rRNA sequencing on fresh frozen tumor and adjacent normal renal tissue from 24 ccRCC patients across stages (n = 28 samples). In parallel, formalin-fixed paraffin-embedded tumor and normal tissue from 31 ccRCC patients across stages and recurrence states were analyzed using tissue microarray (n = 221 cores). Bacterial presence was assessed using fluorescence in situ hybridization (FISH) with universal pan-bacterial peptide nucleic acid (PNA) probes targeting bacterial 16S rRNA and confocal microscopy (LSM980). Bacteria-detected cores were further analyzed using 6000-plex CosMx spatial transcriptomics using paired differential pseudo-bulk expression analysis and pathway-level enrichment analyses.

Results: 16S rRNA sequencing detected bacterial DNA in both tumor and normal tissue. Enhydrobacter was enriched in stage IV tumors (p = 0.03; n = 6 patients), but as a common skin commensal, this may reflect contamination rather than a true biological signal, emphasizing the need for tissue-based validation. In contrast, PNA-FISH detected rare but clear intratumoral bacteria in 12 tumor cores from 9 patients, and no bacterial signal was observed in matched normal renal tissue. Paired spatial transcriptomics (n = 12 tumor region pairs) showed limited gene- and pathway-level significance.

Conclusion: This study provides the first direct, tissue-based visual evidence of bacterial detection in ccRCC tumors. Although bacterial presence appears rare and spatially heterogeneous, the biological relevance of these bacteria remains unclear. Further whole-section analyses and orthogonal validation are needed to determine whether intratumoral bacteria play a role in ccRCC biology and treatment response.

