# Supplementary material for: INTERNATIONAL KIDNEY CANCER SYMPOSIUM: EUROPE
Source: J Kidney Cancer VHL. 2026 Apr 16;13(SP2):7–80. doi: 10.15586/jkcvhl.v13iSP2.480 (PMC13660974; doi:10.15586/jkcvhl.v13iSP2.480)
Supplement: Related texts

# Abstract: SQSTM1/p62 accumulation is a hallmark of FLCN loss in Birt-Hogg-Dubé syndrome-associated kidney cancer

**AUTHORS**

Helene Knævelsrud, Department of Molecular Medicine, Institute of Basic Medical Sciences, and Centre for Cancer Cell Reprogramming, Institute of Clinical Medicine, University of Oslo, Oslo, Norway and Department of Molecular Cell Biology, Institute for Cancer Research, Oslo University Hospital, Oslo, Norway

Halvor Ullern, Department of Molecular Medicine, Institute of Basic Medical Sciences, and Centre for Cancer Cell Reprogramming, Institute of Clinical Medicine, University of Oslo, Oslo, Norway and Department of Molecular Cell Biology, Institute for Cancer Research, Oslo University Hospital, Oslo, Norway

Julie Aarmo Johannessen, Department of Molecular Medicine, Institute of Basic Medical Sciences, and Centre for Cancer Cell Reprogramming, Institute of Clinical Medicine, University of Oslo, Oslo, Norway and Department of Molecular Cell Biology, Institute for Cancer Research, Oslo University Hospital, Oslo, Norway

Feyza Kasikci, Department of Molecular Medicine, Institute of Basic Medical Sciences, and Centre for Cancer Cell Reprogramming, Institute of Clinical Medicine, University of Oslo, Oslo, Norway and Department of Molecular Cell Biology, Institute for Cancer Research, Oslo University Hospital, Oslo, Norway

Miriam Formica, Department of Molecular Medicine, Institute of Basic Medical Sciences, and Centre for Cancer Cell Reprogramming, Institute of Clinical Medicine, University of Oslo, Oslo, Norway and Department of Molecular Cell Biology, Institute for Cancer Research, Oslo University Hospital, Oslo, Norway

Naghmeh Karimi Melve, Department of Molecular Medicine, Institute of Basic Medical Sciences, and Centre for Cancer Cell Reprogramming, Institute of Clinical Medicine, University of Oslo, Oslo, Norway and Department of Molecular Cell Biology, Institute for Cancer Research, Oslo University Hospital, Oslo, Norway

Siri Andresen, Department of Molecular Medicine, Institute of Basic Medical Sciences, and Centre for Cancer Cell Reprogramming, Institute of Clinical Medicine, University of Oslo, Oslo, Norway and Department of Molecular Cell Biology, Institute for Cancer Research, Oslo University Hospital, Oslo, Norway

Andreas Brech, Centre for Cancer Cell Reprogramming, Institute of Clinical Medicine, University of Oslo, Oslo, Norway and Department of Molecular Cell Biology, Institute for Cancer Research, Oslo University Hospital, Oslo, Norway

Karol Axcrona, Department of Urology, Akershus University Hospital, Lørenskog, Norway

Kjersti Jørgensen, Department of Medical Genetics, Oslo University Hospital and University of Oslo, Oslo, Norway

Lorant Farkas, Department of Pathology, Akershus University Hospital, Lørenskog, Norway and Department of Pathology, Oslo University Hospital, Oslo, Norway

Jorrit M. Enserink, Centre for Cancer Cell Reprogramming, Institute of Clinical Medicine, University of Oslo, Oslo, Norway and Department of Molecular Cell Biology, Institute for Cancer Research, Oslo University Hospital, Oslo, Norway and The Department of Biosciences, UiO

Birt-Hogg-Dubé syndrome (BHD) is an autosomal, dominant condition caused by Folliculin (FLCN) mutation and characterized by enhanced risk for kidney tumors. Previous studies have shown constitutive nuclear localization of the transcription factor TFEB and simultaneous hyperactivation of canonical MTORC1 signaling in the absence of FLCN. Here we assess the impact on autophagy under this situation of combined anabolic and catabolic activation. Using an established BHD patient-derived kidney cancer cell line, we confirmed that TFEB was permanently localized in the nucleus combined with an increase in canonical MTORC1 signaling, whereas bulk autophagy flux and LC3 lipidation was unaffected by FLCN status. However, we found that the autophagy receptor SQSTM1/p62 accumulated in enlarged puncta in the absence of FLCN. Finally, we recapitulate these findings in a Norwegian cohort of BHD kidney tumor samples. Our results demonstrate SQSTM1/p62 accumulation as a hallmark of FLCN loss, although SQSTM1/p62 appeared dispensable for anchorage-independent growth.

# Abstract: Autophagy profiling and dependency in renal cell carcinoma patient-derived organoids

**AUTHORS**

Julie Johannessen, University of Oslo, Oslo, Norway; Oslo University Hospital, Oslo, Norway

Feyza Kasikci, University of Oslo, Oslo, Norway

Sampada Bhagwat, University of Oslo, Oslo, Norway

Siri Andresen, University of Oslo, Oslo, Norway

Karl Henrik Østby, Aker Oslo University Hospital, Oslo, Norway

Nicolai Wessel, Aker Oslo University Hospital, Oslo, Norway

Anders Selnes, Aker Oslo University Hospital, Oslo, Norway

Klaudia Orlowska, University of Oslo, Oslo, Norway

Helene Knævelsrud, University of Oslo, Oslo, Norway; Oslo University Hospital, Oslo, Norway

Renal cell carcinoma (RCC), the most common form of kidney cancer, poses significant challenges due to its complex nature and resistance to conventional therapies. Although recent advantages in immunotherapy have improved the outcome for many patients, some patient groups do not respond to the first line treatment. Recent studies have shed light on the role of autophagy, a cellular degradation and recycling process, in the development and progression of RCC. By analyzing a biobank of fresh frozen RCC tumor tissue and adjacent normal tissue, we have identified distinct subtype-specific patterns based on levels of key autophagy markers. To further explore these mechanisms, we have established a protocol to grow patient-derived organoids (PDOs) from both normal kidney and tumor tissue, providing a valuable platform for preclinical studies. Our goal is to investigate the potential autophagy dependency of RCC PDOs by modulating key autophagic pathways, aiming to identify novel therapeutic strategies that could enhance treatment outcomes for RCC patients. We hypothesize that certain subtypes of RCC is sensitive of autophagy modulation. These findings highlight the potential of RCC PDOs as a robust tool for studying the role of autophagy in cancer biology and for developing targeted therapies.

# Abstract: Bioengineered renal cancer bone-metastasis organoid models for drug testing

**AUTHORS**

Nathalie Bock, Queensland University of Technology, Brisbane, Australia

Philip Rowell, Metro South Health, Brisbane, Australia

Ghazaleh Hashemi, Queensland University of Technology, Brisbane, Australia

Anu T. Koikalethu, Queensland University of Technology, Brisbane, Australia

Sophie Napier, Queensland University of Technology, Brisbane, Australia

Lisa Philp, Queensland University of Technology, Brisbane, Australia

Scott Sommerville, Queensland University of Technology, Brisbane, Australia

Erik W. Thompson, Queensland University of Technology, Brisbane, Australia

Introduction: Cell-line and patient-derived organoids (PDOs) are increasingly used to support precision oncology in renal cell carcinoma (RCC), including bone-metastatic disease. However, the commonly used 3D matrix Matrigel® limits biophysical tunability, contains xenogeneic components, and exhibits batch variability, constraining reproducibility and clinical translation. We evaluated tunable, gelatin-based extracellular-matrix–mimicking hydrogels as alternatives for renal cancer bone-metastasis organoid culture and drug testing, hypothesizing improved control over organoid viability and treatment response across cell-line and patient-derived systems.

Methods: Renal cancer organoids derived from the Caki-1 cell line and patient-derived RCC bone-metastasis samples were encapsulated in hydrogels with defined stiffness (approximately 1–5 kPa). Organoids were cultured for three days prior to treatment with doxorubicin for an additional three days. Matrigel and free-floating, no-ECM cultures served as comparators. Organoid size and viability were quantified to assess matrix-dependent growth and drug response.

Results: In Caki-1 spheroids, organoid area remained stable across all hydrogel stiffness conditions, while no-ECM cultures exhibited progressive enlargement. Viability increased with hydrogel stiffness, reaching approximately 250%, 325%, and 550% of baseline levels in low-, medium-, and high-stiffness conditions, respectively, by day 6. In contrast, viability in Matrigel and no-ECM cultures remained comparatively constant (approximately 100–200%). In patient-derived RCC bone-metastasis organoids, no-ECM cultures produced larger organoids, while hydrogel and Matrigel conditions were comparable in size. Viability remained stable across all ECM conditions (approximately 90–100%) with no stiffness-dependent enhancement observed. Doxorubicin reduced viability across all models. Drug-response trends were consistent between cell-line and patient-derived organoids, although greater sensitivity was observed in cell-line systems.

Conclusions: Semi-synthetic hydrogels provide a controllable platform for renal cancer organoid culture and drug testing. Distinct stiffness-dependent effects in cell-line organoids, contrasted with stable responses in patient-derived samples, highlight the importance of ECM context when modelling metastatic RCC and support improved preclinical screening strategies.

# Abstract: Tumor Microenvironment and Metabolic Dysregulation Correlate with Treatment Outcomes in Renal Cell Carcinoma

**AUTHORS**

Poornima Verma, Sanjay Gandhi Post Graduate Institute of Medical Sciences, Lucknow, India

Background: RCC remains largely refractory to therapy, with response rates of 34% for TKI–ICI combinations and 25.7% for ICIs alone. Tumor metabolism and immunosuppressive TME may contribute to resistance, but clinical correlations remain unclear.

Objective: To delineate the association between RCC TME/metabolites and regulatory T cell (Treg) activity with treatment outcomes.

Methods: Sixty RCC patients receiving TKIs, ICIs, or combination therapy were enrolled. Tumor biopsies and peripheral blood samples were collected at baseline and after 8 weeks of therapy. Multiplex immunohistochemistry and flow cytometry quantified Tregs, CD8+ T cells, and myeloid-derived suppressor cells. Targeted metabolomics assessed 50 tumor-associated metabolites, including glycolytic, TCA, lipid, and tryptophan–kynurenine pathway intermediates. Clinical response was evaluated using RECIST 1.1.

Results: Non-responders (n = 34) displayed higher Treg density (mean 18.6% vs. 7.2% in responders, p < 0.001) and increased IL-10/TGF-β expression. CD8+ T cell infiltration was significantly lower in non-responders (12.1% vs. 22.4%, p < 0.01). Metabolomics revealed elevated lactate (2.8-fold), kynurenine (3.1-fold), palmitate (2.2-fold), and reduced α-ketoglutarate (0.6-fold) in non-responders (all p < 0.01). Integrative Treg–metabolite signature predicted therapy response with 82% accuracy (AUC 0.87). Subgroup analysis showed TKI–ICI combination responders had lower lactate/kynurenine ratios and higher CD8/Treg ratios compared with monotherapy responders (p < 0.05).

Conclusion: RCC patients with elevated Treg infiltration and altered tumor metabolite profiles exhibit poor response to targeted and immunotherapies. These findings highlight immune–metabolic biomarkers as predictive tools to guide personalized therapeutic strategies.

# Abstract: NEK5 is a PROTAC-Targetable Hidden Driver in VHL-Deficient Kidney Cancer

**AUTHORS**

Qing, Zhang, UT Southwestern Medical Center

Clear cell renal cell carcinoma (ccRCC) harbors metabolic vulnerabilities beyond canonical HIF signaling, yet the identity and mechanisms of non-genetic dependencies remain largely undefined. Here, Here, by integrating NetBID2-based computational modeling of ccRCC molecular networks pre-trained and fine-tuned across multiple patient RNA-seq cohorts with in vitro and in vivo functional genomicsusing patient-derived regulatory networks (SJARACNe→NetBID2) coupled with in vitro and in vivo functional genomics, we identify never-in-mitosis A–related kinase 5 (NEK5) as a hidden driver selectively required for VHL-deficient ccRCC. NEK5 activity—but not mRNA expression—is elevated in VHL-mutant tumors. Genetic ablation of NEK5 suppresses anchorage-independent growth, delays orthotopic tumor formation, and reduces metastasis, while sgRNA-resistant NEK5 fully restores tumorigenicity. Mechanistically, NEK5 is a mitochondrial protein that binds to the translocator protein TSPO to control respiration, membrane potential, and redox homeostasis. NEK5 loss increases oxygen consumption yet decreases ΔΨm and ATP/ADP ratios, producing uncoupled oxidative phosphorylation and excessive mitochondrial ROS; ROS scavenging restores growth. A kinase-dead NEK5 mutant rescues proliferation, and ATP-competitive inhibition is ineffective, demonstrating a largely kinase-independent mechanism. Epistasis positions TSPO downstream of NEK5, and VHL loss strengthens NEK5–TSPO association, linking this axis to the VHL pathway. Guided by these mechanistic insights, we developed Cereblon-based PROTACs that degrade NEK5, recapitulate the phenotypes of genetic loss, and selectively impair VHL-deficient ccRCC growth. These findings unveil NEK5 as an activity-defined, mitochondria-centered vulnerability in ccRCC and demonstrate how network-inference frameworksnetwork-based activity inference can expose non-enzymatic targets amenable to protein degradation therapies.

# Abstract: Molecular mimicry between gut microbial antigens and tumour-associated antigens as a mechanism of immune checkpoint inhibitor response in metastatic renal cell carcinoma (RCC)

**AUTHORS**

Raquel Ramos, The University of Surrey, Guildford, UK

Anna Krukowska, The University of Surrey, Guildford, UK

Hossam Attia, Royal Surrey County Hospital, Guildford, UK

Agnieszka Michael, The University of Surrey, Guildford, UK

Nicola Annels, The University of Surrey, Guildford, UK

Immune checkpoint inhibitors (ICIs) have transformed the management of metastatic renal cell carcinoma (RCC); however, durable clinical benefit is achieved in only a subset of patients. The gut microbiome has emerged as a predictor of ICI response, but the immunological mechanisms underlying this association remain poorly defined. Molecular mimicry, whereby microbial antigens share homology with tumour-associated antigens (TAAs), represents a plausible mechanism linking microbiome composition to anti-tumour immunity.

We have developed a bioinformatics-driven pipeline that integrates microbial species associated with ICI response and curated renal cancer antigen datasets to predict candidate antigenic mimics. This pipeline is being applied to renal patients initiating ICI-based immunotherapy to identify bacterial species enriched in responders and to prioritise microbial antigens with predicted homology to RCC TAAs.

To support these analyses, a prospective longitudinal study is ongoing in patients with metastatic RCC receiving ICI-based therapies, including combination regimens. Stool and blood samples are collected at baseline and longitudinally during treatment. Planned analyses include stool metagenomics and metabolomics, peripheral blood immune phenotyping, plasma profiling, and tumour immunohistochemistry. Following completion of patient recruitment, predicted microbial mimics will be assessed experimentally by stimulating peripheral blood mononuclear cells to measure cross-reactive CD8^+^ T cell responses.

This project is at an early stage, with patient recruitment and sample collection ongoing and computational workflows under implementation and validation. Biological validation and clinical correlation analyses are pending. We will present the overall study design and integrated analytical framework linking computational discovery with prospective clinical and immunological investigation, with the aim of defining microbiome-driven mechanisms of immunotherapy response in RCC.

# Abstract: The effectiveness of drug combination on treatment clear cell carcinoma compared to papillary renal carcinoma

**AUTHORS**

Samy L. Habib, South Texas Veterans Health Care System, San Antonio, TX, USA

Kidney Cancer is the 7th leading cause of cancer and one of the most lethal cancers in the USA. There are several cell phonotypes of kidney cancer including clear cell renal cell carcinoma (ccRCC), papillary (pRCC) and chromophobe (chRCC). ccRCC is the most common (∼80%) subtype while papillary RCC (pRCC) is the second most common subtype with frequency of ∼15%, of malignant kidney tumors. ACHN cells is a model of papillary (pRCC) while SN-12 cells and 786-O cells are models for ccRCC. The current treatment of kidney cancer patients showed no survival benefit of using sorafenib or sunitinib alone. This study designed to test efficiency of sunitinib or rapamycin or drugs combination on regulation cell apoptosis, cell proliferation, cell migration and cell invasion. Sunitinib treatment at 2.5 and 5 μM dose showed significant decrease in cell proliferation to 90-95%, while rapamycin treatment showed decrease to 25-40% in 786-O and SN-12 cells, respectively compared untreated cells for 24hrs. While treatment ACHN cells with sunitinib or rapamycin showed decrease 60-70% and 25-35%, respectively compared to control cells. On the other hands, sunitinib treatment showed increase in cell apoptosis to 250-300% in 786-O cells and 200-300% in SN-12 cells, while rapamycin showed increase 100-120% in 786-O cells, 100-150 % in SN-12 cells respectively. While treatment ACHN cells with sunitinib or rapamycin showed decrease in cell proliferation 100-120% compared with control cells. Drug combination showed sharp decrease in cell proliferation in SN12 and 786-O cells to 90-99%, respectively compared to 70% in ACHN cells. On the other hands, drug combination increases cell apoptosis to 400% in SN-12 cells compared to 200 and 300 in ACHN and 786-O cells, respectively. Sunitinib alone showed significant decrease in cell invasion and cell migration in SN-12 and 786-O cells (60-70%, respectively) and 40-50% in ACHN cells compared to rapamycin (30% in SN-12 and 40 and 20% in 786-O and ACHN cells, respectively). While drug combination showed sharp decrease in cell invasion and cell migration to 70 and 90% in SN-12 and 786-O cells, respectively, and 50% in ACHN cells. In summary, this study demonstrates the effectiveness of drug combination on treatment patients with clear cell carcinoma compared to patients with papillary renal carcinoma. The outcome of the study will help the drug selection for treatment kidney cancer patient with different type of renal cancer.

# Abstract: AI-assisted Delphi Consensus: An Italian multidisciplinary expert working group effort for patients with RCC

**AUTHORS**

Sarah Scagliarini, Medical Oncology Unit, AORN Cardarelli Hospital, Naples, Italy

Brigida Anna Maiorano, Department of Medical Oncology, IRCCS San Raffaele Hospital, Milan, Italy

Veronica Mollica, Medical Oncology, IRCCS Azienda Ospedaliero-Universitaria di Bologna, Bologna, Italy

Pasquale Rescigno, Department of Oncology, Candiolo Cancer Institute FPO-IRCCS, Turin, Italy; Centre for Cancer, Translational and Clinical Research Institute, Newcastle, UK

Riccardo Campi, Unit of Urology and Renal Transplantation, Careggi University Hospital, Florence, Italy

Matteo Santoni, Medical Oncology Unit, Macerata General Hospital, Macerata, Italy

Roberto Contieri, Department of Urology and Gynecology, Istituto Nazionale Tumori IRCCS Fondazione G. Pascale, Naples, Italy

Luigi Formisano, Department of Clinical Medicine and Surgery, University of Naples Federico II, Naples, Italy

Sara Elena Rebuzzi, Medical Oncology Unit 2, Ospedale Molinette, Azienda Ospedaliero-Universitaria Città della Salute e della Scienza di Torino, Turin, Italy

Davide Bimbatti, Oncology 1 Unit, Istituto Oncologico Veneto IOV - IRCCS, Padua, Italy

Giulia Marvaso, Department of Oncology and Hemato-Oncology, University of Milan, Milan, Italy; Radiotherapy Division, IRCCS European Institute of Oncology, Milan, Italy

Marilena Di Napoli, Department of Urology and Gynecology, Istituto Nazionale Tumori IRCCS Fondazione G. Pascale, Naples, Italy

Marco Stellato, Genitourinary Medical Oncology, Fondazione IRCCS Istituto Nazionale dei Tumori di Milano, Milan, Italy

Umberto Capitanio, IRCCS San Raffaele Scientific Institute, Urological Research Institute (URI), Milan, Italy

Luca Tagliaferri, Radiotherapy Division, Catholic University, CCC of Rome, Italy

Mimma Rizzo, Medical Oncology Unit, Azienda Ospedaliera Universitaria Consorziale Policlinico di Bari, Bari, Italy

Vincenza Conteduca, Department of Medical and Surgical Sciences, Unit of Medical Oncology and Biomolecular Therapy, University of Foggia, Policlinico Riuniti, Foggia, Italy

Claudia Ungarelli, International Kidney Cancer Coalition (IKCC) and ANTURE Patient Advocacy Group

Sebastiano Buti, Medical Oncology Unit, University Hospital of Parma, Parma, Italy

Francesco Massari, Medical Oncology, IRCCS Azienda Ospedaliero-Universitaria di Bologna, Bologna, Italy

Massimo Di Maio, Medical Oncology at Molinette Hospital; Professor of Medical Oncology at University of Turin; AIOM President

Roberto Iacovelli. Associate Professor of Medical Oncology at Catholic University, CCC of Rome, Italy

People with cancer deserve a multidisciplinary approach and a consistent treatment plan, independent of the treating center. The management of kidney cancer has become increasingly complex with the introduction of adjuvant therapies and novel first-line treatments, resulting in major decision-making challenges. Despite evidence from randomized clinical trials and real-world data, current guidelines still leave relevant grey areas to physician discretion. In 2025, two European Delphi consensus studies in renal cell carcinoma were conducted, while the American AUC3 consensus was only recently published. Based on the uncertainties highlighted by these initiatives, an Italian multidisciplinary working group of 41 experts on RCC, including medical oncologists, urologists, and radiation oncologists, led one of the first AI-assisted Delphi consensus in oncology. The aim of this pilot study was to leverage artificial intelligence to overcome intrinsic limitations of the Delphi method while preserving methodological rigor. Consensus was defined as an agreement level greater than 85%. AI support enabled the creation of a virtual evidence library, identification of unresolved clinical scenarios, analysis of open-ended expert feedback, and algorithmic quantification of agreement using Certainty and Contradiction Indices, allowing iterative reformulation of statements between rounds. Two voting rounds were conducted between July and September 2025. Forty-four statements were validated for the first round and grouped into five domains: locoregional disease, adjuvant therapy, relapse after adjuvant therapy, metastatic first-line treatment, and subsequent lines. After the first vote, 18 statements required rewording, 2 were rejected, and 24 were approved. In October 2025, an in-person meeting with experts, patient advocacy representatives, and patients discussed consensus results and unmet needs. Consensus exceeded 90% for most surgical and systemic scenarios, whereas disagreement persisted in post-surgical residual disease and oligoprogression definitions, including during adjuvant therapy. This Italian pilot study confirms AI-assisted Delphi consensus as a robust tool to validate homogeneous recommendations for RCC patients.



# Abstract: Real world use of Artificial Intelligence for automated prognostication and systemic therapeutic decision making in localized RCC

**AUTHORS**

Kenneth Barker, 1. Mayo Clinic, Phoenix, AZ, USA

Umair Ayub, Mayo Clinic, Phoenix, AZ, USA

Muhammad Humayun, Mayo Clinic, Phoenix, AZ, USA

Talha Tahir, Mayo Clinic, Phoenix, AZ, USA

Irbaz Riaz, Mayo Clinic, Phoenix, AZ, USA

Background: Validated prognostic models (Leibovich 2018, SSIGN, UISS) and KEYNOTE-564 risk stratification guide surveillance and adjuvant therapy decisions in localized clear-cell renal cell carcinoma (ccRCC), yet adoption remains limited by workflow barriers. AI-assisted EMR tools may enable rapid, standardized risk assessment.

Methods: We retrospectively analyzed 92 patients with localized ccRCC who underwent radical nephrectomy and oncology evaluation (December 2021– September 2025) across three Mayo Clinic sites. Pathology reports and oncology notes were extracted via SQL queries. A zero-shot GPT-4o pipeline parsed clinical and pathologic variables. Expert oncology clinicians determined gold-standard prognostic classifications using validated calculators and KEYNOTE-564 eligibility criteria. AI outputs used rule-based algorithms implementing published scoring definitions with large language model variable extraction (no model training). AI performance and novice manual scoring by two first-year fellows were compared against expert assessment using accuracy, precision, recall, and F1 scores.

Results: Among 92 patients (median age 64 years), AI demonstrated near-perfect agreement with expert assessment for KEYNOTE-564 adjuvant immunotherapy eligibility (accuracy 97%, precision 100%, recall 97%, F1 98%). For three-class prognostic stratification, AI achieved perfect performance for Leibovich disease-free survival risk (100% all metrics). SSIGN performance was strong (accuracy 91%, precision 91%, recall 93%, F1 92%), comparable to novice abstraction (accuracy 92%, precision 89%, recall 91%, F1 90%). UISS binary classification showed 81% accuracy, 74% precision, 100% recall, and 85% F1, with a tendency toward false-positive classification.

Conclusions: AI-enabled EMR tools accurately automate prognostic risk stratification and adjuvant therapy eligibility after nephrectomy for localized ccRCC. Prospective validation and development of multimodal AI platforms integrating pathologic, radiologic, and clinical data are planned.

# Abstract: Survival Outcomes of Fecal Microbiota Transplantation Plus Pembrolizumab and Axitinib in Metastatic Renal Cell Carcinoma: Extended follow up analysis of the TACITO trial

**AUTHORS**

Daniela Arduini, Department of Medical and Surgical Sciences, UOC Oncologia Medica, Fondazione Policlinico Universitario Agostino Gemelli IRCCS, Rome, Italy

Chiara Ciccarese, Department of Medical and Surgical Sciences, UOC Oncologia Medica, Fondazione Policlinico Universitario Agostino Gemelli IRCCS, Rome, Italy; Department of Translational Medicine and Surgery, Università Cattolica del Sacro Cuore, Rome, Italy

Denis Occhipinti, Department of Translational Medicine and Surgery, Università Cattolica del Sacro Cuore, Rome, Italy

Serena Porcari, Department of Medical and Surgical Sciences, UOC CEMAD Centro Malattie dell'Apparato Digerente, Medicina Interna e Gastroenterologia, Fondazione Policlinico Universitario Gemelli IRCCS, Rome, Italy

Debora Rondinella, Department of Medical and Surgical Sciences, UOC CEMAD Centro Malattie dell'Apparato Digerente, Medicina Interna e Gastroenterologia, Fondazione Policlinico Universitario Gemelli IRCCS, Rome, Italy

Gianluca Quaranta, Department of Laboratory and Hematology Sciences, Fondazione Policlinico Universitario A. Gemelli IRCCS, Rome, Italy

Andrea Severino, Department of Medical and Surgical Sciences, UOC CEMAD Centro Malattie dell'Apparato Digerente, Medicina Interna e Gastroenterologia, Fondazione Policlinico Universitario Gemelli IRCCS, Rome, Italy

Sebastiano Buti, Department of Medicine and Surgery, University of Parma, Parma, Italy

Giuseppe Fornarini, UO Oncologia Medica 1, IRCCS Ospedale Policlinico San Martino, Genoa, Italy

Francesca Primi, Medical Oncology, Central Hospital of Belcolle, Viterbo, Italy

Luciano Stumbo, Department of Medical Oncology, Campus Bio-Medico University of Rome, Rome, Italy

Diana Giannarelli, Facility di Epidemiologia e Biostatistica, Fondazione Policlinico Universitario A. Gemelli IRCCS, Rome, Italy

Maurizio Sanguinetti, Department of Laboratory and Hematology Sciences, Fondazione Policlinico Universitario A. Gemelli IRCCS, Rome, Italy; Department of Basic Biotechnological Sciences, Intensive and Perioperative Clinics, Università Cattolica del Sacro Cuore, Rome, Italy

Luca Masucci, Department of Laboratory and Hematology Sciences, Fondazione Policlinico Universitario A. Gemelli IRCCS, Rome, Italy; Department of Basic Biotechnological Sciences, Intensive and Perioperative Clinics, Università Cattolica del Sacro Cuore, Rome, Italy

Antonio Gasbarrini, Department of Medical and Surgical Sciences, UOC Gastroenterologia, Fondazione Policlinico Universitario Agostino Gemelli IRCCS, Rome, Italy; Department of Translational Medicine and Surgery, Università Cattolica del Sacro Cuore, Rome, Italy

Giampaolo Tortora, Department of Medical and Surgical Sciences, UOC Oncologia Medica, Fondazione Policlinico Universitario Agostino Gemelli IRCCS, Rome, Italy; Department of Translational Medicine and Surgery, Università Cattolica del Sacro Cuore, Rome, Italy

Laurence Zitvogel, Department CIBIO, University of Trento, Trento, Italy

Nicola Segata, Department CIBIO, University of Trento, Trento, Italy

Gianluca Ianiro, Department of Medical and Surgical Sciences, UOC Gastroenterologia, Fondazione Policlinico Universitario Agostino Gemelli IRCCS, Rome, Italy; Department of Translational Medicine and Surgery, Università Cattolica del Sacro Cuore, Rome, Italy

Roberto Iacovelli, Department of Medical and Surgical Sciences, UOC Oncologia Medica, Fondazione Policlinico Universitario Agostino Gemelli IRCCS, Rome, Italy; Department of Translational Medicine and Surgery, Università Cattolica del Sacro Cuore, Rome, Italy

Introduction: Treatment for metastatic renal cell carcinoma (mRCC) is based on immune checkpoint inhibitor (ICI) combinations; however, durable long-term survival remains limited for many patients. Increasing evidence supports a role for the gut microbiome in modulating ICI efficacy. The TACITO trial reported as fecal microbiota transplantation (FMT) from complete ICI-responders increased PFS over placebo in mRCC receiving pembrolizumab plus axitinib.

Methods: TACITO is an investigator-initiated, randomized, double-blind, placebo-controlled phase 2 trial, enrolling mRCC patients treated with first-line pembrolizumab plus axitinib. Patients were randomized to receive donor FMT (dFMT) or placebo FMT (p-FMT) three times in 6 months, unless disease progression occurred. Both groups underwent the first procedure by colonoscopy, followed by capsulized FMT after 12 weeks and 24 weeks, respectively. We reported here the updated values for progression free survival (PFS) and overall survival (OS) and safety.

Results: Forty-five patients were randomized to d-FMT (23 pts) or p-FMT (22 pts). After a median follow-up for PFS of 33.0 months, 33 patients progressed (16 vs. 17). The median PFS was 23.2 months (95%CI, 9.5 – 36.9) in the d-FMT arm compared to 8.7 months (95%CI, 2.2 – 15.3); this difference was significant (p=0.043). In patients with favorable IMDC risk, the median values for PFS was 26.9 months for d-FMT and was not reached in the p-FMT arm (p=0.78). In patients with intermediate/poor IMDC risk, the median PFS was respectively 14.3 vs. 5.1 months (p=0.031). After a median follow up for OS of 40.0 months, 22 patients died, 8 in the d-FMT arm and 14 in the p-FMT arm. The median OS was 45.6 months (95%CI, NR – NR) in the d-FMT arm compared to 28.3 months (95%CI, 16.4 – 40.1), (p=0.052). In patients with favorable IMDC risk, the median values for OS was 45.6 months for d-FMT and was not reached in the p-FMT arm (p=0.37). In patients with intermediate/poor IMDC risk, the median values for PFS were respectively 41.9 vs. 21.5 months (p=0.062). At extended follow-up safety profile remained unchanged.

Conclusions: Extended follow-up of TACITO trial confirmed that donor FMT combined with pembrolizumab and axitinib provides sustained improvements in PFS and OS in treatment-naïve mRCC, reinforcing the potential of microbiome modulation as a strategy to enhance ICI efficacy.

# Abstract: Clear-cell versus non–clear-cell metastatic renal cell carcinoma treated with sunitinib: similar progression-free survival but divergent overall survival in a real-world cohort

**AUTHORS**

Mehdi Alem, Hassan II University Hospital, Faculty of Medicine, Pharmacy and Dentistry, Fez, Morocco

Sara Nejjari, Hassan II University Hospital, Faculty of Medicine, Pharmacy and Dentistry, Fez, Morocco

Mounir Belcadi, Hassan II University Hospital, Faculty of Medicine, Pharmacy and Dentistry, Fez, Morocco

Lamiae Amaadour, Hassan II University Hospital, Faculty of Medicine, Pharmacy and Dentistry, Fez, Morocco

Karima Oualla, Hassan II University Hospital, Faculty of Medicine, Pharmacy and Dentistry, Fez, Morocco

Zineb Benbrahim, Hassan II University Hospital, Faculty of Medicine, Pharmacy and Dentistry, Fez, Morocco

Samia Arifi, Hassan II University Hospital, Faculty of Medicine, Pharmacy and Dentistry, Fez, Morocco

Nawfel Mellas, Hassan II University Hospital, Faculty of Medicine, Pharmacy and Dentistry, Fez, Morocco

Background: Clear-cell renal cell carcinoma (ccRCC) and non–clear-cell renal cell carcinoma (non-ccRCC) represent biologically distinct entities with different responses to systemic therapies. Although sunitinib is a standard first-line treatment for metastatic RCC, data comparing its efficacy and tolerability between ccRCC and non-ccRCC in real-world settings remain limited. This study aimed to compare clinical outcomes and safety profiles of sunitinib between ccRCC and non-ccRCC patients treated in routine practice.

Methods: This retrospective study analyzed 69 patients with metastatic renal cell carcinoma (RCC) who received sunitinib treatment at the Medical Oncology Department of Hassan II University Hospital in Fès, from January 1, 2014, to June 31, 2025. Patients were divided into two groups: clear-cell RCC (ccRCC) and non-clear-cell RCC (non-ccRCC). The study compared overall survival (OS), progression-free survival (PFS), and objective response rate (ORR) between the two groups. Survival outcomes were assessed using the Kaplan-Meier method, and treatment response was evaluated according to RECIST criteria. All statistical analyses were performed using SPSS version 27.0. A p-value < 0.05 was considered statistically significant.

Results: Among the 69 patients, 53 (76.8%) had ccRCC and 16 (23.2%) had nonccRCC. The mean age was 58.8 years, and all patients received sunitinib as first-line therapy. According to IMDC criteria, 58% of patients were classified as poor risk. With a median follow-up of 58.8 months, PFS did not significantly differ between ccRCC and non-ccRCC patients. The mean PFS was 38.7 months (95% CI: 25.7– 51.8) in the ccRCC group and 73.7 months (95% CI: 59.7–87.7) in the non-ccRCC group, with no statistically significant difference (log-rank p=0.197). In contrast, OS was significantly longer in patients with non-ccRCC compared with ccRCC. Median OS was 27.6 months in the non-ccRCC group versus 3.6 months in the ccRCC group, with mean OS estimates of 35.6 months (95% CI: 14.6–56.6) and 17.5 months (95% CI: 9.6–25.3), respectively (log-rank p=0.044). The overall objective response rate (ORR) was higher in the ccRCC group, with 45% of patients showing a partial response (PR) and 10% showing stable disease (SD). In contrast, the nonccRCC group had an ORR of 30%, with 15% of patients showing a PR and 20% showing SD. The difference in response rate between the two groups was not statistically significant (p = 0.089). Treatment-related adverse events were frequent. Grade 3–4 toxicities occurred in 50.7% of patients. The most commonly reported adverse events included fatigue (29%), hypertension (30.4%), hematological toxicity (24.6%), hand–foot syndrome (18.8%), mucositis (14.5%), and vomiting (13%). No histology-specific safety signals were identified.

Conclusions: In this real-world cohort of metastatic RCC treated with first-line sunitinib, PFS was comparable between ccRCC and non-ccRCC patients. However, non-ccRCC histology was associated with significantly longer overall survival. The safety profile of sunitinib was consistent across histological subtypes, with a high incidence of grade 3–4 toxicities. These findings highlight the prognostic heterogeneity of RCC histologies and support the need for histology-adapted therapeutic strategies.



# Abstract: Reproducing COMPARZ Trial in real-world practice: non-inferiority of pazopanib versus sunitinib as first-line treatment for metastatic renal cell carcinoma

**AUTHORS**

Mehdi Alem, Hassan II University Hospital, Faculty of Medicine, Pharmacy and Dentistry, Fez, Morocco

Sara Nejjari, Hassan II University Hospital, Faculty of Medicine, Pharmacy and Dentistry, Fez, Morocco

Mounir Belcadi, Hassan II University Hospital, Faculty of Medicine, Pharmacy and Dentistry, Fez, Morocco

Lamiae Amaadour, Hassan II University Hospital, Faculty of Medicine, Pharmacy and Dentistry, Fez, Morocco

Karima Oualla, Hassan II University Hospital, Faculty of Medicine, Pharmacy and Dentistry, Fez, Morocco

Zineb Benbrahim, Hassan II University Hospital, Faculty of Medicine, Pharmacy and Dentistry, Fez, Morocco

Samia Arifi, Hassan II University Hospital, Faculty of Medicine, Pharmacy and Dentistry, Fez, Morocco

Nawfel Mellas, Hassan II University Hospital, Faculty of Medicine, Pharmacy and Dentistry, Fez, Morocco

Background: Metastatic renal cell carcinoma (mRCC) remains a major therapeutic challenge. Pazopanib and sunitinib, two tyrosine kinase inhibitors, have demonstrated comparable efficacy as first-line treatment, as shown in the COMPARZ trial. This study aimed to compare overall survival (OS), progression-free survival (PFS), objective response rate (ORR), and safety profiles of pazopanib and sunitinib in a real-world cohort of patients with mRCC treated at a North African tertiary center.

Methods: This retrospective cohort study included patients diagnosed with mRCC and treated at the Medical Oncology Department of CHU Hassan II, Fez, Morocco, between January 1, 2014 and December 31, 2023. Eligible patients received either pazopanib or sunitinib as first-line therapy and had complete medical records. OS, PFS, and ORR were compared between treatment groups. Survival outcomes were estimated using the Kaplan–Meier method, and tumor response was assessed according to RECIST criteria. Statistical analyses were performed using SPSS version 27.0. A p-value <0.05 was considered statistically significant.

Results: A total of 91 patients with mRCC were included, of whom 22 (24.2%) received pazopanib and 69 (75.8%) received sunitinib as first-line treatment. The median age was 58.9 years (range 17–84), with a male predominance (70.4%). Most patients had an ECOG performance status of 1 (54.9%). Clear cell carcinoma was the predominant histological subtype (80.2%), and Fuhrman grade 2 was the most frequent (34.1%). The most common metastatic sites were lymph nodes (56%), followed by lung and liver metastases (48.4% each). According to the IMDC classification, 53.8% of patients were in the poor-risk group. Median PFS was 10.3 months (95% CI: 9.3–11.2) in the pazopanib group and 9.9 months (95% CI: 9.3– 10.5) in the sunitinib group (HR 1.08; 95% CI: 0.85–1.38; p=0.039), confirming non-inferiority. Median OS was 23.7 months (95% CI: 22.2–25.2) with pazopanib and 23.0 months (95% CI: 21.8–24.2) with sunitinib (HR 1.04; 95% CI: 0.82–1.31; p=0.556). ORR was comparable between pazopanib and sunitinib (32.1% vs 30.8%, p=0.88). Stable disease was observed in 42.9% and 46.2% of patients, respectively, while progressive disease occurred in 25.0% and 23.1%. Adverse events were reported in 75.0% of patients receiving pazopanib and 84.6% of those receiving sunitinib. Grade 3–4 toxicities were more frequent with sunitinib (37.5%) than with pazopanib (18.4%), although the difference was not statistically significant (p=0.48). The most common toxicities included fatigue, hypertension, diarrhea, and hand–foot syndrome. Dose reductions due to adverse events were required in 21.4% of patients in the pazopanib group versus 34.6% in the sunitinib group.

Conclusions: In this real-world cohort, pazopanib and sunitinib demonstrated comparable efficacy as first-line treatment for mRCC. However, pazopanib was associated with a more favorable safety profile, in line with results from the COMPARZ trial.



# Abstract: A single arm phase 2 study of sasanlimab, palbociclib and axitinib in metastatic renal cell carcinoma - SPARCC

**AUTHORS**

Prateek Khanna, Dana-Farber Cancer Institute, Boston, MA, USA

Bradley McGregor, Dana-Farber Cancer Institute, Boston, MA, USA

Srinivas Viswanathan, Dana-Farber Cancer Institute, Boston, MA, USA

Michael Serzan, Dana-Farber Cancer Institute, Boston, MA, USA

Wenxin Xu, Dana-Farber Cancer Institute, Boston, MA, USA

Bicky Thapa, Dana-Farber Cancer Institute, Boston, MA, USA

Wanling Xie, Dana-Farber Cancer Institute, Boston, MA, USA

Toni Choueiri, Dana-Farber Cancer Institute, Boston, MA, USA

Stephanie Berg, Dana-Farber Cancer Institute, Boston, MA, USA

Background: Clear cell renal cell carcinoma (ccRCC) is characterized by loss of VHL through mutational inactivation or hypermethylation1. In addition, mutation, deletion or hypermethylation of CDKN2A, a negative regulator of CDK4/6, has been observed in 15% of ccRCC which is associated with worse survival2. Synthetic lethality between decreased CDK4/6 activity and VHL loss has been described in diverse human ccRCC cell lines and xenografts3. Moreover, in translocation renal cell carcinoma (tRCC), a rare and aggressive variant histology, CDK4/6 have been shown to be activated downstream of TFE3 translocation4. In preclinical studies, CDK4/6 inhibitors have shown in vitro activity against ccRCC and tRCC5 while combination with a VEGFR-TKI (abemaciclib with sunitinib) led to tumor reduction in murine xenografts5. Moreover, CDK4/6 inhibition has demonstrated immunomodulatory properties with potential synergism with anti-PD(L)-1 therapies6,7. Current study evaluates the efficacy of combining palbociclib, a CDK4/6 inhibitor; with axitinib, a VEGFR-TKI; and sasanlimab, a PD-1 inhibitor.

Methods: This multi-center, single-arm, phase II study evaluates the safety and efficacy of combination treatment with palbociclib, axitinib, and sasanlimab in patients with metastatic renal cell carcinoma (NCT07123090). Patients with unresectable advanced or ccRCC or tRCC are eligible. Patients will receive sasanlimab 300 mg s.c. every 4 weeks (1 cycle length), with palbociclib 75 mg daily week 2-4 every cycle, and axitinib 5 mg twice daily. The primary objective is to determine the overall response rate of the combination treatment. Secondary objectives include estimating the rate of complete or deep partial response (>80% tumor shrinkage per RECIST1.1), progression free survival and overall survival and evaluating safety of the combination treatment. The study will employ a single-arm one stage design with planned sample size of 25 patients. The study began enrollment in multiple sites across the United States beginning December 2025.

# Abstract: Microbiome impacting therapy with inulin gel in combination with ipilimumab and nivolumab in advanced renal cell carcinoma: ICON trial

**AUTHORS**

Ulka Vaishampayan, University of Michigan, Ann Arbor, MI, USA

John Rice, University of Michigan, Ann Arbor, MI, USA

Sarah Yentz, University of Michigan, Ann Arbor, MI, USA

Irene Tsung, University of Michigan, Ann Arbor, MI, USA

Cristiane Bergerot, Grupo Oncoclínicas, São Paulo, Brazil

James Moon, University of Michigan, Ann Arbor, MI, USA

Background: The gut microbiome has been shown to impact the safety and efficacy of immune checkpoint inhibitor (ICI) therapy. Inulin is a plant-derived prebiotic dietary fiber that significantly enhanced systemic anti-tumor efficacy of αnti-PD-1 therapy and led to decreased rates of immunotherapy induced colitis in preclinical models. A colon retentive preparation of inulin gel was developed at the University of Michigan. A study in healthy volunteers established the tolerability of inulin gel. Based on preclinical data demonstrating enhanced ICI efficacy and favorable clinical tolerability, this clinical trial was designed to evaluate the combination of inulin gel with ipilimumab and nivolumab in patients with advanced clear cell renal cancer.

Study Design: Patient Eligibility. Eligible patients have advanced clear cell or sarcomatoid RCC with no prior systemic anticancer therapy for metastatic disease. Adjuvant pembrolizumab is permitted if completed > 6 months prior to study enrollment. Patients must be candidates for ipi-nivo as determined by their treating physician. Performance status of 0-1 and adequate bone marrow, renal and liver function are required.

Methods: After an initial safety run-in of 6 patients receiving the combination, patients are randomized 2:1 to the combination of ipilimumab (ipi) 1mg/kg + nivolumab (nivo) 3mg/kg (ipi-nivo) and inulin gel 10g twice daily vs ipi+nivo standard of care therapy. The primary endpoint is the proportion of patients free of progression at 6 months. Secondary endpoints include response rate, overall survival, and incidence of immune related and other toxicities. Correlative objectives include assessment of liquid biopsy and baseline tissue genomic sequencing for prognostic and predictive markers, measuring the change in short chain fatty acid levels in stool and changes in the microbiome. Quality of life will be evaluated by patient reported surveys. We anticipate enrollment of 48 patients. The sample size is not sufficiently large to do a formal hypothesis test between the two randomized arms in the study. A confidence interval for the difference in the response rate and progression free survival between the two arms will be provided. The analysis will provide the preliminary data required to consider planning a larger randomized trial. Clinical trial information: NCT06866262 Supported by DOD Clinical trial award KC220225



# Abstract: Farnesyl transferase inhibitor (FTI) darlifarnib (KO-2806) combined with cabozantinib (cabo) in clear cell renal cell carcinoma (ccRCC) patients after prior exposure to cabo: Preliminary phase 1 results from FIT-001 (NCT06026410)

**AUTHORS**

Yousef Zakharia, Mayo Clinic Comprehensive Cancer Center, Rochester, MN, USA

Adam E. Singer, Division of Hematology-Oncology, University of California Los Angeles Medical Center, Los Angeles, CA, USA

Benjamin Garmezy, Sarah Cannon Research Institute, Nashville, TN, USA

Jacob Thomas, Division of Medical Oncology, Department of Medicine, University of Southern California Norris Comprehensive Cancer Center, Los Angeles, CA, USA

Douglas E. Laux, Division of Hematology, Oncology and Blood & Marrow Transplantation, University of Iowa Hospitals and Clinics, Iowa City, IA, USA

Jason Henry, Sarah Cannon Research Institute at Health One, Denver, CO, USA

Liza Villaruz, UPMC Hillman Cancer Center, Pittsburgh, PA, USA

Sanjay Goel, Rutgers Cancer Institute of New Jersey, New Brunswick, NJ, USA

Paria Mahboub Johnson, Clinical Development, Kura Oncology, Inc., San Diego, CA, USA

Jenchun Kuan, Biometrics, Kura Oncology, Inc., San Diego, CA, USA

Binaifer Balsara, Clinical Development, Kura Oncology, Inc., San Diego, CA, USA

Jabed Seraj, Clinical Development, Kura Oncology, Inc., San Diego, CA, USA

Mollie Leoni, Clinical Development, Kura Oncology, Inc., San Diego, CA, USA

Adanma Ayanambakkam, Stephenson Cancer Center, University of Oklahoma Health Sciences Center, Oklahoma City, OK, USA

Background: Efficacy limitations in 2L+ ccRCC highlight the need for improved therapeutic options for non-responders and for those who progress on VEGFR-TKI. Preclinical studies have shown darlifarnib resensitizes cabo-exposed ccRCC by blocking mTORC1 activation. Clinical data in ccRCC (ESMO’25, 2604P) support the potential of a darlifarnib+cabo combination. Here we report safety and preliminary antitumor activity of the combination in prior cabo-exposed ccRCC patients.

Methods: Darlifarnib 3, 5 or 8mg (QD, PO, 7d on/off) + cabo 40mg or 60mg (QD, PO) was evaluated in 28d cycles. Patients must have received ≥1 prior IO-based treatment. Primary objective: safety; secondary objectives: antitumor activity, pharmacokinetics.

Results: As of 8 Dec 2025, 70 RCC patients were enrolled; median age: 68y; 79% male. Most common (≥20%) any-grade darlifarnib-related AEs were neutropenia (41%), fatigue (30%), diarrhea and nausea (26% each), and thrombocytopenia (21%). Most common (≥10%) grade ≥3 darlifarnib-related AE was neutropenia (30%). Among ccRCC patients with prior cabo exposure (n=18), 56% (n=10) had cabo as the immediate prior line and 67% (n=12) had received additional prior line(s) of TKI. In 16 prior cabo-exposed response-evaluable patients, ORRa was 43.8% and DCRb was 93.8% (Table). Five of the 7 responders had BORc of SDd on prior cabo treatment, and 4 out of those 5 had received cabo as the immediate prior line. Longest duration of response observed was 11.2 months. Updated data to be presented.

Conclusions: This combination demonstrated a manageable safety profile and promising antitumor activity in ccRCC patients with prior cabo exposure. Activity of the combination in a post-cabo setting is suggestive of darlifarnib’s contribution to antitumor activity in this underserved patient population. These data support further development of darlifarnib and cabo in ccRCC.



# Abstract: Treatment Barriers and Shared Decision-Making Disparities in Kidney Cancer Across Europe: Results from the IKCC Global Patient Survey

**AUTHORS**

Eric Jonasch, MD Anderson Cancer Center, Houston, TX, USA

Olivier Exertier, International Kidney Cancer Coalition (IKCC), Amsterdam, Netherlands

Deborah Maskens, International Kidney Cancer Coalition (IKCC), Amsterdam, Netherlands

Axel Bex, Royal Free London NHS Foundation Trust, University College London, Division of Surgery and Interventional Science, London, UK; The Netherlands Cancer Institute, Amsterdam, Netherlands

Saeed Dabestani, Department of Urology, Kristianstad Central Hospital, Region Skane, Kristianstad, Sweden; Department of Translational Medicine, Division of Urological Cancers, Lund University, Lund, Sweden

Selcuk Erdem, Division of Urologic Oncology, Department of Urology, Istanbul University Istanbul Faculty of Medicine, Istanbul, Turkey

Margaret Hickey, International Kidney Cancer Coalition (IKCC), Amsterdam, Netherlands

Karin Kastrati, Nierenkrebs-Netzwerk Deutschland e.V., Germany

Roman Sosnowski, Department of Urology and Oncological Urology, Warmian-Masurian Cancer Center, Olsztyn, Poland

Yüksel Ürün, Ankara University School of Medicine, Department of Medical Oncology, Ankara, Turkey

Michael Jewett, Princess Margaret Cancer Centre and Department of Surgery (Urology), University of Toronto, Toronto, Canada

The International Kidney Cancer Coalition (IKCC) conducted the 2025 Global Patient Survey to characterize patient experiences and identify unmet needs in kidney cancer. We report findings on shared decision making (SDM), disease-related knowledge, and treatment barriers, comparing respondents from Europe with those from the rest of the world (RoW) and assessing variability across European countries. The survey was developed by an IKCC steering committee with patient advocates, medical experts, and the Picker Institute, cognitively tested, translated into 16 languages, and administered online and on paper. Data were analysed using cross-tabulations. Between September 24 and November 15, 2024, 2,677 global responses were received, including 1,247 from Europe. European respondents were more likely than those in RoW to report involvement in SDM to their desired level (60% vs 52%, p<0.001), with substantial variation across European countries (73% in Italy to 46% in Poland). Compared with RoW, Europeans more frequently reported completely understanding their cancer stage (62% vs 55%) and kidney cancer subtype (46% vs 42%), reported similar understanding of recurrence risk, treatment options, and treatment recommendations, but lower understanding of survival probability (32% vs 38%, p=0.004). Fewer Europeans reported barriers influencing treatment choices than RoW (41% vs 67%, p<0.001); however, marked country-level differences were observed. Long wait times were the most frequently reported barrier in Europe (15% overall; range 5% in France to 48% in Poland). Despite higher reported SDM involvement, clinically meaningful knowledge gaps and access barriers persist across Europe, with considerable heterogeneity between countries. These findings underscore the need for enhanced clinician–patient communication, improved patient education, timely access to care, and country-specific interventions to reduce disparities.

# Abstract: Evaluating worldwide disparities in clinical trials in patients with localized renal cell carcinoma

**AUTHORS**

Jaya Goud, City of Hope Comprehensive Cancer Center, Duarte, CA, USA

Ruchi Agarwal, City of Hope Comprehensive Cancer Center, Duarte, CA, USA

Koral Shah, City of Hope Comprehensive Cancer Center, Duarte, CA, USA

Daniela Castro, City of Hope Comprehensive Cancer Center, Duarte, CA, USA

Xiaochen Li, City of Hope Comprehensive Cancer Center, Duarte, CA, USA

Miguel Zugman, City of Hope Comprehensive Cancer Center, Duarte, CA, USA

Teebro Paul, City of Hope Comprehensive Cancer Center, Duarte, CA, USA

Samuel Dickter, City of Hope Comprehensive Cancer Center, Duarte, CA, USA

Lea Dickter, City of Hope Comprehensive Cancer Center, Duarte, CA, USA

Lily Lau, City of Hope Comprehensive Cancer Center, Duarte, CA, USA

Aaron Lee, City of Hope Comprehensive Cancer Center, Duarte, CA, USA

Nasr Chaudhary, City of Hope Comprehensive Cancer Center, Duarte, CA, USA

Salvador Jaime-Casas, City of Hope Comprehensive Cancer Center, Duarte, CA, USA

Benjamin Mercier, City of Hope Comprehensive Cancer Center, Duarte, CA, USA

JoAnn Hsu, MD Anderson Cancer Center, Houston, TX, USA

Nazli Dizman, City of Hope Comprehensive Cancer Center, Duarte, CA, USA

Alexander Chehrazi-Raffle, City of Hope Comprehensive Cancer Center, Duarte, CA, USA

Abhishek Tripathi, City of Hope Comprehensive Cancer Center, Duarte, CA, USA

Regina Barragan-Carrillo, City of Hope Comprehensive Cancer Center, Duarte, CA, USA

Sumanta Pal, City of Hope Comprehensive Cancer Center, Duarte, CA, USA

Clinical trials for renal cell carcinoma (RCC) are essential for improving patientoutcomes, but remain heavily concentrated in high-income countries (HICs). We aimedto assess the global availability of clinical trials for localized RCC and evaluatedisparities by country income level. We queried ClinicalTrials.gov to identify all studies enrolling patients with RCC fromJune 2019 to June 2024. Noninterventional, pediatric, and metastatic trials wereexcluded. Participating countries were classified by income based on World BankRanking (WBR): HICs, upper-middle-income (UMICs), lower-middle-income (LMICs),and low-income countries (LICs). Data were collected on disease stage, histology, study phase, sponsor type, and multinational status. The Kruskal-Wallis test assesseddifferences in country-level characteristics across WBR groups. Multivariable logisticregression assessed associations between country characteristics and trial availability. A total of 71 eligible trials investigating localized RCC were identified. The majority(73.2%) were conducted exclusively in HICs (HIC-only trials), while 19 trials (26.8%)included sites in non-high-income countries (non-HICs). Academic sponsorship wasmore frequent in non-HIC trials compared with HIC-only trials (84.2% vs. 73.1%). Funding from industry was more frequent in HIC-only trials compared with non-HICtrials (23.1% vs. 15.8%). Despite early-phase (I, II) studies remaining the most commonacross all settings, late-phase (III, IV) studies were more frequent among HIC-only trials (11.5% vs. 5.3%). In multivariable analysis, higher gross national income (OR 18.54, 95% CI 3.07-111.90; p=0.002) and greater percentage of health expenditure (OR 1.56, 95% CI 1.05–2.32; p=0.027) were significantly associated with increased availability of clinical trials. Clinical trials for localized RCC are overwhelmingly centered in HICs, specifically inwealthier countries with greater investment in healthcare. These disparities underscorethe need to expand research infrastructure in resource-limited settings to improverepresentation and ensure advances in care are globally accessible.

# Abstract: Key service elements needing improvement: The Kidney Cancer UK Patient survey

**AUTHORS**

Malcolm Packer, Kidney Cancer UK, UK

Andrew Greaves, Royal Free Hospital, London, UK

Ekaterini Boleti, Royal Free Hospital, London, UK

Natalie Chanley, 3. Royal Preston Hospital, Preston, UK

Background: The first ever national evidence-based guidance on kidney cancer covering England and Wales were launched in March 2026. This was the result of extended campaigning by Kidney Cancer UK, and we sought to capture patients’ experiences of services prior to guidelines’ launch.

Methods: We have been performing an annual patient survey for 12 years to measure renal cancer patient experience. We used our latest survey to examine key areas that we anticipated the guidelines would address. The survey form included 73 multiple choice and 5 free text questions. Publicised via the Charity’s website and social media platforms, questionnaires were available online, and sent by post, on request.

Results: We received 945 responses. Results revealed that 26% patients were initially misdiagnosed in primary care. More than half (54%) of the diagnosed cases were detected as incidental findings, during investigations for an unrelated condition. One hundred and sixty seven patients (20%) were diagnosed when their cancer reached stage 4. In 546 (66%) of cases biopsy was not offered to patients. Of the … (87%) patients who had surgery, … (77%) had radical nephrectomy, an increase over the previous year with a corresponding reduction in partial nephrectomies. Only 64 (40%) of stage 4 patients reported being offered systemic therapy. Most patients (88%) were involved in decisions about their treatment, but 20% were unhappy with information and support before and after surgery and 29% reported not receiving enough information about systemic therapy side-effects.

Conclusions: Our survey reveals areas requiring improvement throughout the kidney cancer pathway, including diagnosis, treatment, and patient information and support. The newly drafted national NICE guidance needs to include all areas of kidney cancer diagnosis and management and will require continuous evaluation, for example through the National Kidney Cancer Audit.

# Abstract: Bridging Gaps in Kidney Cancer Care in Rural India: A Patient Advocacy and Care Navigation Model from a Resource-Limited Setting

**AUTHORS**

Aditya Manna, MAS Clinic & Hospital, Tamluk, WB, India

Saptaparna Jana, Aditya's Cancer Care, Tamluk, WB, India

Kidney cancer outcomes in low- and middle-income countries are significantly influenced by delayed diagnosis, limited access to specialized oncology services, and socioeconomic barriers. In rural India, these challenges are compounded by poor health literacy, financial toxicity, geographic inaccessibility, and fragmented care pathways. This abstract describes a real-world, patient-centered kidney cancer care and advocacy model developed and implemented in a rural Indian setting by an oncology social worker and patient advocate with over two decades of experience in community oncology and palliative care. My role encompasses early patient navigation, risk counseling, coordination of diagnostics and referrals, treatment adherence support, management of treatment-related toxicities, and integration of palliative and survivorship care for patients with localized and advanced renal cell carcinoma. Working closely with oncologists, surgeons, and multidisciplinary teams, I facilitate shared decision-making, address psychosocial distress, and mitigate financial barriers through counseling and linkage to support resources. Special emphasis is placed on follow-up continuity, symptom monitoring, and caregiver education, particularly for patients receiving systemic therapies or living with metastatic disease. Observational insights from this rural practice highlight frequent late-stage presentation, underutilization of nephron-sparing approaches, treatment interruptions due to economic hardship, and limited access to immunotherapy. However, structured patient advocacy and community-based support have demonstrated improvements in treatment compliance, symptom control, patient-reported outcomes, and end-of-life care planning. This experience underscores the critical role of non-physician patient advocates in strengthening kidney cancer care delivery in resource-constrained settings. Integrating advocacy, navigation, and palliative principles into routine oncology practice may offer a scalable pathway to reduce disparities and improve equity in global kidney cancer care.



# Abstract: Identifying Aggressiveness of Kidney Cancer by Infrared Visualization

**AUTHORS**

Besarion Partsvania, Georgian Technical University, Tbilisi, Georgia

Vera Jeladze, Georgian Technical University, Tbilisi, Georgia

Tamaz Sulaberidze, Clinic “JERARSI”, Tbilisi, Georgia Alexandre Khuskivadze, Clinic “JERARSI”, Tbilisi, Georgia

Sophia Abazadze, Georgian Technical University, Tbilisi, Georgia

Accurate intraoperative assessment of kidney cancer aggressiveness is essential for surgical decision-making, influencing the choice between nephron-sparing surgery and radical nephrectomy, resection margins, and recurrence risk. However, reliable differentiation between indolent and aggressive renal tumors during surgery remains challenging, as macroscopic appearance often fails to reflect tumor biology. Intraoperative pathological techniques are limited by processing time and workflow disruption, creating a need for rapid, non-invasive, and biologically meaningful intraoperative tools. In this study, we present a rapid infrared (IR) visualization approach for assessing kidney cancer aggressiveness. Kidney tissue samples obtained after nephrectomy were examined using an infrared transmission system based on 850 nm light-emitting diodes and a CCD infrared camera. Transmitted IR signals were processed using custom-developed software giving spatial variations in tissue absorption. IR findings were correlated with histopathological analysis using corresponding tissue sections. For each specimen, average illumination ratios between malignant and adjacent non-malignant regions were calculated, and statistical significance was evaluated using Student’s t-test. Total analysis time per sample did not exceed 5–6 minutes. Infrared visualization revealed reproducible spatial patterns associated with renal tissue type and tumor aggressiveness. Fibrotic and low-grade tissues demonstrated relatively uniform IR transmission, whereas high-grade clear-cell and sarcomatoid carcinomas exhibited pronounced signal attenuation and heterogeneity. The correspondence between histopathological grading and IR darkening supports IR visualization as a rapid, non-contact, and label-free method for whole-surface assessment of kidney tumors, with potential application as a complementary intraoperative tool for real-time oncologic decision-making.



# Abstract: Specific in vivo detection of V2R-positive metastatic ccRCC using a toxin-based PET radioligand

**AUTHORS**

Nicolas Gilles, CEA, Joliot Institute, Paris, France

Goran Stanajic-Petrovic, CEA, Joliot Institute, Paris, France

Khawla Schmeis, CEA, Joliot Institute, Paris, France

Charles Truillet, CEA, Joliot Institute, Paris, France

The diagnosis of metastatic clear cell renal cell carcinoma (mccRCC) remains challenging due to the lack of highly specific diagnostic tools. We validate an imaging modality for mccRCC based on the first radioligand targeting the type 2 vasopressin receptor (V2R). V2R is ectopically expressed in several tumor types, including mccRCC. This imaging approach utilizes [18F]F-MQ232, a radiolabeled peptide derived from snake venom, which exhibits high in vivo selectivity for V2R. The V2R-selective peptide MQ232 conjugated with either a cyanine 5 (Cy5) or a fluorine 18 (18F) bearing prosthetic group were synthetized. V2R-coding RNA expression was quantitatively assessed while V2R protein expression was evaluated by flow cytometry using Cy5-MQ232. The tumor targeting and selectivity of the modified MQ232 peptides were assessed using in vivo fluorescence imagery in tumor-bearing mice, developed from cells expressing V2R at different levels. Pharmacological properties of [18F]F-MQ232 were determined in rodents by metabolic evaluation and PET pharmacokinetic analysis. V2R-positive tumor labeling and imaging capabilities were evaluated in human Caki-1 xenografted mice. [18F]F-MQ232 is a highly relevant radioligand whose tumor uptake directly correlates with V2R expression levels in tissues, demonstrating its specificity to V2R-expressing tumors. Replacing the peptide moiety by an isoform unable to interact with V2R leads to a drastic decrease in the radioligand’s tumor uptake, highlighting its origin in a specific, ligand/receptor type interaction between the MQ232 moiety and V2R. PET/CT imaging of Caki-1 xenografted mice demonstrated the ability of [18F]F-MQ232 to allow specific detection of the tumor compartment. RT-qPCR screening of metastatic and non-metastatic ccRCC biopsies from patients confirms V2R expression, emphasizing the scope of such a new imaging modality development. This work validates the V2R-targeting strategy for mccRCC using [18F]F-MQ232 and demonstrates that human mccRCC tissues express V2R, confirming the suitability of such a specific imaging technique for metastasis extension assessment.



# Abstract: Provider Perceptions of Cure in Renal Cell Cancer (RCC)

**AUTHORS**

Dena Battle, KCCure, Alexandria, VA, USA

Laurence Albiges, Gustave Roussy Cancer Campus, University of Paris Sud, Villejuif, France

Ithaar Derweesh, Rush University, Chicago, IL, USA

Meghan Griffith, KCCure, Alexandria, VA, USA

Alex Kutikov, Fox Chase Cancer Center, Philadelphia, PA, USA

Lisa Pickering, Royal Marsden Hospital, London, UK

Elizabeth Plimack, Fox Chase Cancer Center, Philadelphia, PA, USA

Sarah Psutka, University of Washington, Seattle, WA, USA

Daniel Shapiro, University of Wisconsin, Madison, WI, USA

Ulka Vaishampayan, University of Michigan, Ann Arbor, MI, USA

Tian Zhang, UT Southwestern Medical Center, Dallas, TX, USA

Michael Staehler, University of Munich, Munich, Germany

Introduction and Objectives: Following a prior KCCure patient survey on perceptions of cure, we conducted a survey to assess physicians’ views on cure in renal cell carcinoma (RCC).

Methods: The survey was distributed at the ESMO 2025 Congress via QR code on a KCCure poster about patient perspectives on cure, emailed to key opinion leaders in GU oncology, and shared through targeted social media outreach.

Results: A total of 177 providers participated (105 medical oncologists, 65 urologists, 7 radiation oncologists); 85% practiced in academic centers. While 78% reported that >75% of patients with localized RCC can be cured, only 9% felt similarly about locally advanced disease and 2% about metastatic disease. Overall, 75% of physicians were hesitant to use the word “cure,” consistent across specialties. Major barriers included uncertainty about long-term outcomes (87%) and lack of a consistent definition (75%). Compared with medical oncologists, urologists more often cited emotional reactions from patients (31% vs 16%, p=0.007) and concern about loss to follow-up (24% vs 9%, p=0.0024). Urologists were also more likely to select “never” when asked when a patient could be considered cured; 47% felt metastatic patients should never be told they are cured versus 26% of medical oncologists. Regarding acceptable recurrence risk, 71% selected <20%, 50% selected <5%, and 19% chose “other,” suggesting individualized criteria.

Conclusions: Substantial hesitancy surrounds use of the term “cure” among RCC specialists. Even when cure rates are perceived as high, physicians remain reluctant to use the term, mirroring patient reservations. Standardizing definitions and communication around “cure” in RCC may improve clarity, alignment, and shared decision-making.

# Abstract: Neoadjuvant axitinib plus avelumab in patients with high risk localized clear cell renal cell carcinoma: the NeoAvAx trial

**AUTHORS**

Anne de Ruijter, Division of Molecular Carcinogenesis, The Netherlands Cancer Institute, Amsterdam, Netherlands

J.C. Koen van der Mijn, Division of Medical Oncology, The Netherlands Cancer Institute, Amsterdam, Netherlands

Niels Graafland, Department of Urology, The Netherlands Cancer Institute, Amsterdam, Netherlands

Hans van Thienen, Division of Medical Oncology, The Netherlands Cancer Institute, Amsterdam, Netherlands

Sofie Wilgenhof, Division of Medical Oncology, The Netherlands Cancer Institute, Amsterdam, Netherlands

Benoit Nicolet, Division of Molecular Oncology & Immunology, The Netherlands Cancer Institute, Amsterdam, Netherlands

Maurits van Montfoort, Department of Pathology, The Netherlands Cancer Institute, Amsterdam, Netherlands

Brunolf Lagerveld, Department of Urology, OLVG, Amsterdam, Netherlands

Patricia Zondervan, Department of Urology, Amsterdam University Medical Center, Amsterdam, Netherlands

Harrie Beerlage, Department of Urology, Amsterdam University Medical Center, Amsterdam, Netherlands

Jeroen van Moorselaar, Department of Urology, Amsterdam University Medical Center, Amsterdam, Netherlands

Ilayda Hasakiogullari, CellCarta, Wilrijk, Belgium

Mark Kockx, CellCarta, Wilrijk, Belgium

Pieter-Jan van Dam, CellCarta, Wilrijk, Belgium

Bernadett Szabados, Royal Free London NHS Foundation Trust, London, UK

Christian U. Blank, Division of Medical Oncology, The Netherlands Cancer Institute, Amsterdam, Netherlands

Thomas Powles, Royal Free London NHS Foundation Trust, London, UK; Barts Cancer Institute, Queen Mary University of London, London, UK

Daniel J. Vis, Division of Molecular Carcinogenesis, The Netherlands Cancer Institute, Amsterdam, Netherlands

Lodewyk Wessels, Division of Molecular Carcinogenesis, The Netherlands Cancer Institute, Amsterdam, Netherlands

John B.A.G. Haanen, Division of Medical Oncology, The Netherlands Cancer Institute, Amsterdam, Netherlands

Axel Bex, Department of Urology, The Netherlands Cancer Institute, Amsterdam, Netherlands; Royal Free London NHS Foundation Trust, London, UK; Barts Cancer Institute, Queen Mary University of London, London, UK

Neoadjuvant immunotherapy is more effective than adjuvant treatment in melanoma and lung cancer, but limited data are available for renal cell carcinoma (RCC). In NeoAvAx, a single arm phase II clinical study, 40 patients with high risk localized clear cell renal cell carcinoma (ccRCC) received 3 months of axitinib (VEGFR1-3 inhibitor) and avelumab (anti-PDL1) followed by nephrectomy. The primary endpoint was safety and radiographic response rate (RECIST 1.1) to systemic treatment. All patients were evaluable for the study endpoints and underwent surgical resection within 14 weeks of starting systemic therapy. Ten (25%) patients had a radiographic partial response with a median downsizing of the primary tumor of 18.7% (0-43.9%). No new safety signals were seen with Grade 3-4 adverse events in 6 (15%) patients. Fifteen (38%) patients showed a pathologic response of which three (7.5%) patients had a major pathologic response (<10% vital tumor). After a median follow-up of 52.9 months, a recurrence occurred in 22 patients (55%), and the median disease-free (DFS) survival was 25.1 months. Seven (18%) patients died of ccRCC, all of whom were pathologic non-responders. Pathological responders showed higher levels of various innate immune cells, while TLS and activated DC signatures associated with improved DFS. Treatment induced CD8 T-cell infiltration into the tumor in both pathological responders and non-responders (p=0.019). The persistence of expanded TCR-clones during treatment is associated with improved DFS (log-rank p=0.014, Cox PH HR=0.29, p=0.0041). Patients with a higher post-treatment TCR-diversity demonstrate improved DFS (log-rank p=0.002, Cox PH HR=0.125, p=0.037). Neoadjuvant treatment is safe, induces deep pathological responses in a subset of patients and may promote the development of long-lasting immune responses. Information about the pathologic response, gene expression signatures and CD8 T-cell influx may help to design personalized adjuvant therapies and individualized surveillance protocols.

# Abstract: Your Blood Knows: Immune Receptor Repertoires Predict Outcomes in Localized RCC

**AUTHORS**

Christina Espeseth Grand, Department of Molecular Medicine, Aarhus University, Aarhus, Denmark

Laura Iisager, Department of Molecular Medicine, Aarhus University, Aarhus, Denmark

Jesper Jespersen, Department of Molecular Medicine, Aarhus University, Aarhus, Denmark

Cecilie Lindgaard, Department of Molecular Medicine, Aarhus University, Aarhus, Denmark

Anna Krarup Keller, Department of Urology, Aarhus University Hospital, Aarhus, Denmark

Tommy Kjærgaard Nielsen, Department of Urology, Aalborg University Hospital, Aalborg, Denmark

Mie G. Thorlund, Department of Urology, Odense University Hospital, Odense, Denmark

Niels Fristrup, Department of Oncology, Aarhus University Hospital, Aarhus, Denmark

Iben Lyskjær, Department of Molecular Medicine, Aarhus University, Aarhus, Denmark

Background: Despite curative-intent surgery, a substantial proportion of patients with localized renal cell carcinoma (RCC) develop recurrence, and current risk stratification remains limited. Given the immunogenic nature of RCC, systemic immune features may reflect tumor biology and provide non-invasive prognostic biomarkers.

Methods: Peripheral blood was collected at diagnosis from 134 patients with localized RCC. Genomic DNA from buffy coats underwent high-throughput sequencing of individual T-cell receptor (TCR α, β, γ, δ) and B-cell receptor (BCR heavy, κ, λ) chains. Immune diversity and clonality were quantified. Simpson diversity indices were used to assess immune repertoire diversity. Patients were stratified by repertoire features. Associations with survival were evaluated using Kaplan–Meier and multivariable Cox models adjusted for age and sex.

Results: TCRα diversity provided clinically meaningful prognostic information, refining risk stratification beyond the Leibovich score in intermediate-risk patients. Low TCRα diversity identified a subgroup with markedly worse overall survival (median split, log-rank p = 0.0018, n = 50). BCR diversity also showed risk-group-specific associations. In high-risk patients, higher BCR diversity was associated with worse overall survival (lowest quartile versus highest quartile, p = 0.015, n=26), but not in intermediate-risk patients (lowest quartile versus the remaining, p = 0.38, n = 51). In intermediate-risk patients, multivariable Cox models adjusted for age and sex (n = 50) showed that TCRα and BCR-heavy diversity were independently associated with overall survival. Higher TCRα diversity (HR = 0.63, p = 0.019) and higher BCR-heavy diversity (HR = 0.62, p = 0.006) were each associated with reduced mortality risk (per standard deviation increase).

Conclusion: Circulating immune repertoire profiling provides non-invasive prognostic information in localized RCC. In this 134-patient cohort, strong T- and B-cell signals were detected, and ongoing recruitment will expand the dataset to validate them.

# Abstract: Impact of intratumoral tertiary lymphoid structures on response and toxicity to immune checkpoint inhibitors from the BIONIKK trial in metastatic clear cell renal cell carcinoma

**AUTHORS**

Isaias Hernández-Verdin, Centre de Recherche des Cordeliers, Sorbonne Université, INSERM, Université Paris Cité, Paris, France

Cheng-Ming Sun, Dana-Farber Cancer Institute, Boston, MA, USA

Maxime Meylan, Dana-Farber Cancer Institute, Boston, MA, USA

Mostefa Bennamoun, Department of Medical Oncology, Institut Mutualiste Montsouris, Paris, France

Virginie Verkkare, Département d’Anatomopathologie, Hôpital Européen Georges Pompidou, Assistance Publique-Hôpitaux de Paris – Université Paris Cité, Paris, France

Laure Fournier, Département de Radiologie, Hôpital Européen Georges Pompidou, Assistance PubliqueHôpitaux de Paris – Université Paris Cité, Paris, France

Marine Gross-Goupil, Department of Medical Oncology, Centre Hospitalier Universitaire de Bordeaux - Hôpital Saint-André, Bordeaux, France

Christophe Tournigand, Department of Medical Oncology, Hôpital Henri-Mondor, AP-HP Centre -Université de Paris Est, Créteil, France; INSERM, IMRB, Créteil, France

Delphine Borchiellini, Department of Medical Oncology, Centre Antoine Lacassagne, Université Côte d'Azur, Nice, France

Brigitte Laguerre, Department of Medical Oncology, Centre Eugene - Marquis, Rennes, France

Denis Maillet, Department of Medical Oncology, IMMUCARE, Centre Hospitalier Lyon Sud, Institut de Cancérologie des Hospices de Lyon (IC-HCL), Pierre-Bénite, France; Faculté de Médecine Jacques Lisfranc, Saint Etienne, France

Philippe Barthélémy, Department of Medical Oncology, Institut de Cancérologie Strasbourg Europe, Strasbourg, France

Gwenaelle Gravis, Department of Medical Oncology, Institut Paoli-Calmettes, Aix-Marseille University, CRCM, Marseille, France

Elodie Coquan, Department of Medical Oncology, Centre de Lutte Contre le Cancer François Baclesse, Caen, France

Diane Pannier, Department of Medical Oncology, Centre Oscar Lambret, Lille, France

Nadine Houede, Department of Medical Oncology, Gard Cancer Research Institute, Nîmes Caremeau University Hospital, Montpellier University, Nîmes, France

Stéphane Oudard, Département de Cancérologie Médicale, Hôpital Européen Georges Pompidou, Assistance Publique-Hôpitaux de Paris – Université Paris Cité, Paris, France

Catherine Sautès-Fridman, Centre de Recherche des Cordeliers, Sorbonne Université, INSERM, Université Paris Cité, Paris, France

Wolf Herman Fridman, Centre de Recherche des Cordeliers, Sorbonne Université, INSERM, Université Paris Cité, Paris, France

Yann-Alexandre Vano, Département de Cancérologie Médicale, Hôpital Européen Georges Pompidou, Assistance Publique-Hôpitaux de Paris – Université Paris Cité, Paris, France

Immune checkpoint inhibitors (ICI) have improved outcomes in metastatic clear cell renal cell carcinoma (ccRCC), particularly in tumors harboring tertiary lymphoid structures (TLS), yet clinical benefit and immune-related adverse events (AEs) remain unclear. Identifying tumor immune features that predict both efficacy and toxicity is therefore critical. Using samples from the BIONIKK trial, we investigated how tumor immune organization and TLS influence outcomes in patients treated with anti-PD-1, anti-PD-1 plus anti-CTLA-4, or tyrosine kinase inhibitors (TKIs).

Transcriptomic profiling of 199 tumors identified an immune-based signature (PRISMA) enriched in immune-infiltrated tumors and strongly associated with response to PD-1–based therapies, particularly anti-PD-1+CTLA-4. PRISMA-high patients exhibited higher response rates (64% versus 35% responders, p=0.007), and significantly prolonged progression-free (PFS, 16.2 versus 7.8 months, p=0.003) and overall survival (not-reached versus 45.9 months, p=0.024), as well as delayed time to subsequent therapy (32.6 versus 10.6 months, p=0.002) under combination immunotherapy, while no benefit was observed with TKIs.

Immunohistochemical analyses showed that PRISMA-positive tumors were enriched in intratumoral TLS and adaptive immune cell populations. Stratification of TLS-positive tumors by GABA activity revealed distinct clinical phenotypes: TLS-positive GABA-low tumors were associated with improved response (79% versus 9% responders, p<0.001) and PFS under ICI (24.0 versus 6.3 months, p<0.001), compared to TLS-positive GABA-high tumors.

Imoreover, skin, renal, and nervous system AEs were differentially enriched according to tumor TLS-GABA profiles, with distinct temporal patterns following ICI initiation. Multivariate logistic regression adjusting for immune infiltration markers confirmed that TLS-GABA groups independently predicted the occurrence of specific AE categories (Odds-Ratio=3.84, CI95%: 1.01-16.68, p=0.05), supporting a direct link between intratumoral immune organization and systemic immune toxicity.

Altogether, these findings indicate that intratumoral immune architecture and metabolic modulation jointly shape both efficacy and toxicity of ICIs in ccRCC, enabling biologically informed treatment selection and AE risk.

# Abstract: Angiogenesis related blood biomarkers of response to immune checkpoint inhibitors (I) and VEGFR-TKI combination in Renal Cell Carcinoma (RCC): results from the ANGIOCOR prospective trial

**AUTHORS**

Zoé Guillaume, Department of Medical Oncology, Hôpital Européen Georges Pompidou, APHP.Centre – Université de Paris, Paris, France; Paris Cardiovascular Research Center, INSERM, Université de Paris Cité, Paris, France

Marie Auvray-Kuentz, Department of Medical Oncology, Hôpital Européen Georges Pompidou, APHP.Centre – Université de Paris, Paris, France; Paris Cardiovascular Research Center, INSERM, Université de Paris Cité, Paris, France

Loïc Jaffrelot, Department of Medical Oncology, Pitié Salpêtrière Hospital, Assistance Publique-Hôpitaux de Paris (AP-HP) – Sorbonne Université, Paris, France

Louis-Marie Garcin, Department of Medical Oncology, Hôpital Européen Georges Pompidou, APHP.Centre – Université de Paris, Paris, France; Paris Cardiovascular Research Center, INSERM, Université de Paris Cité, Paris, France

Maud Velev, Department of Medical Oncology, Hôpital Européen Georges Pompidou, APHP.Centre – Université de Paris, Paris, France; Paris Cardiovascular Research Center, INSERM, Université de Paris Cité, Paris, France

Olivier Huillard, Department of Medical Oncology, Hôpital Cochin, APHP.Centre – Université de Paris, Paris, France

Carolina Saldana, Department of Medical Oncology, Centre Hospitalier Universitaire Henri Mondor, Assistance Publique-Hôpitaux de Paris (AP-HP), Créteil, France

Sébastien Bertil^6^, Biological Hematology, Hôpital Européen Georges Pompidou, APHP.Centre – Université de Paris, Paris, France

Hamid Lardjani, Department of Medical Oncology, Hôpital Européen Georges Pompidou, APHP.Centre – Université de Paris, Paris, France; Paris Cardiovascular Research Center, INSERM, Université de Paris Cité, Paris, France

Katia Merad, Department of Medical Oncology, Hôpital Européen Georges Pompidou, APHP.Centre – Université de Paris, Paris, France; Paris Cardiovascular Research Center, INSERM, Université de Paris Cité, Paris, France

Dominique Helley, Paris Cardiovascular Research Center, INSERM, Université de Paris Cité, Paris, France; Biological Hematology, Hôpital Européen Georges Pompidou, APHP.Centre – Université de Paris Cité, Paris, France

Eric Tartour, Paris Cardiovascular Research Center, INSERM, Université de Paris Cité, Paris, France; Biological Immunology, Hôpital Européen Georges Pompidou, APHP.Centre – Université de Paris Cité, Paris, France

Constance Thibault, Department of Medical Oncology, Hôpital Européen Georges Pompidou, APHP.Centre – Université de Paris, Paris, France; Paris Cardiovascular Research Center, INSERM, Université de Paris Cité, Paris, France

Stéphane Oudard, Department of Medical Oncology, Hôpital Européen Georges Pompidou, APHP.Centre – Université de Paris, Paris, France; Paris Cardiovascular Research Center, INSERM, Université de Paris Cité, Paris, France

Laetitia Mauge, Paris Cardiovascular Research Center, INSERM, Université de Paris Cité, Paris, France; Biological Hematology, Hôpital Européen Georges Pompidou, APHP.Centre – Université de Paris, Paris, France

In the evolving therapeutic landscape in RCC, predictive biomarkers of efficacy are necessary. The ANGIOCOR trial prospectively assessed the predictive role of circulating angiogenesis biomarkers (CAB). This multicenter non-interventional clinical trial (NCT05285579) included naïve RCC patients (pts) treated according to ESMO guidelines, either with IO (nivolumab + ipilimumab, NI) or IO+TKI combinations. Biomarkers such as VEGF-A, sVEGFR1&2, sNeuropilin-1 (NRP-1), angiopoietin 2 (Ang-2), PlGF, PDGF-BB, sVCAM1, sPD-L1, anhydrase carbonic (CA9) and MMP-9 were assessed at baseline using Luminex or ELISA assays. CAB levels were compared between treatment groups (Kruskal-Wallis test) and according to progression-free survival (PFS) (Mann Whitney and Fischer’s exact tests). This study was sponsored by AP-HP and supported by FONCER contre le Cancer and A.R.T.u.R. Eighty-two pts were included: median age 67 yo; 72% male; 88.5% clear-cell RCC, 87% metastatic stage, IMDC risk score favorable/intermediate/poor for 23%/50%/27%. Baseline levels of CAB were not different between pts treated with NI (n=37) or IO+TKI (n=45, n=7 pembrolizumab+axitinib, n=14 nivolumab+cabozantinib, n=24 pembrolizumab+lenvatinib). Considering PFS, pts with PFS<12mo in the IO+TKI group (13/45) presented higher levels of VEGF-A, sPD-L1, sNRP-1, Ang-2 and Carbonic anhydrase (CA)-9 than pts with PFS>12mo (p<0.05). In the IO group, pts with PFS<12mo (18/37) presented higher levels of Ang-2 and CA-9 (p<0.05). Conversely, higher levels of VEGF-A, sVEGFR1, sPD-L1 or sNRP-1 were associated with a higher rate of pts with a PFS<12mo in the IO+TKI group. More pts had a PFS<12mo among the pts with the highest level of Ang-2, whatever the treatment (p<0.05). This study revealed association between CAB levels and progression status at 12mo particularly in the group IO+TKI. Based on these results, ongoing exploration aims to decipher mechanisms that could explain resistance to IO+TKI combinations.

# Abstract: Genome-wide DNA Methylation Profiling Identifies Prognostic Epigenetic Subtypes and a Novel Risk Score in Clear Cell Renal Cell Carcinoma

**AUTHORS**

Laura Iisager, Department of Molecular Medicine, Aarhus University Hospital, Aarhus, Denmark and Department of Clinical Medicine, Aarhus University, Aarhus, Denmark

Cecilie Lindgaard, Department of Molecular Medicine, Aarhus University Hospital, Aarhus, Denmark and Department of Clinical Medicine, Aarhus University, Aarhus, Denmark

Johanne Ahrenfeldt, Department of Molecular Medicine, Aarhus University Hospital, Aarhus, Denmark and Department of Clinical Medicine, Aarhus University, Aarhus, Denmark

Jesper Jespersen, Department of Molecular Medicine, Aarhus University Hospital, Aarhus, Denmark and Department of Clinical Medicine, Aarhus University, Aarhus, Denmark

Karoline Kondrup, Department of Molecular Medicine, Aarhus University Hospital, Aarhus, Denmark and Department of Clinical Medicine, Aarhus University, Aarhus, Denmark

Laura Zanini, Department of Molecular Medicine, Aarhus University Hospital, Aarhus, Denmark and Department of Clinical Medicine, Aarhus University, Aarhus, Denmark

Anna K. Keller, Department of Clinical Medicine, Aarhus University, Aarhus, Denmark and Department of Urology, Aarhus University Hospital, Aarhus, Denmark

Line Raaby, Department of Molecular Medicine, Aarhus University Hospital, Aarhus, Denmark and Department of Clinical Medicine, Aarhus University, Aarhus, Denmark and Department of Pathology, Aarhus University Hospital, Aarhus, Denmark

Mie G. Thorlund, Department of Urology, Odense University Hospital, Odense, Denmark

Karina D. Sørensen, Department of Molecular Medicine, Aarhus University Hospital, Aarhus, Denmark and Department of Clinical Medicine, Aarhus University, Aarhus, Denmark

Niels Fristrup, Department of Clinical Medicine, Aarhus University, Aarhus, Denmark and Department of Oncology, Aarhus University Hospital, Aarhus, Denmark

Iben Lyskjær, Department of Molecular Medicine, Aarhus University Hospital, Aarhus, Denmark and Department of Clinical Medicine, Aarhus University, Aarhus, Denmark

Background: Clear cell renal cell carcinoma (ccRCC) is characterized by marked molecular heterogeneity and unpredictable clinical behavior. Current prognostic models primarily rely on clinical and histopathological features but lack sufficient precision to guide individualized patient management. We investigated whether genome-wide DNA methylation profiling, integrated with copy number alterations (CNAs), could improve risk stratification in ccRCC.

Methods: We performed methylated DNA immunoprecipitation sequencing (MeDIP-seq) on 116 primary ccRCC tumors and 34 matched normal kidney samples, achieving genome-wide coverage including regulatory and repetitive regions not captured by array-based platforms. CNAs and tumor purity were inferred from low-pass whole-genome sequencing. Differentially methylated regions (DMRs) were identified, and a novel methylation score (MethScore) was derived from tumor-enriched hypermethylated regions. Associations with clinicopathological variables and survival were assessed and validated using TCGA KIRC data where possible.

Results: ccRCC tumors displayed global hypomethylation with focal hypermethylation at key chromosomal arms, including 3p, 9p, and 14q. Hypermethylation on chromosome 3p was significantly enriched in patients with recurrence or metastatic disease, independent of CNAs. Clustering of methylation profiles identified an aggressive epigenetic subtype associated with poor survival (HR 3.13, p < 0.001). A MethScore based on 2,424 hypermethylated DMRs increased with stage and metastatic status and was strongly predictive of overall survival. In multivariable Cox regression including age and stage, MethScore remained independently associated with overall survival (HR 1.24, P = 0.038), confirming its prognostic value beyond established clinical parameters. High MethScore also stratified poor outcomes in localized cases (HR 1.65, P = 0.003), outperforming traditional risk models.

Conclusions: Genome-wide methylation profiling reveals clinically relevant epigenetic subtypes and identifies a robust methylation-based prognostic score in ccRCC. These findings support the integration of epigenetic biomarkers into future risk stratification frameworks, particularly to guide adjuvant treatment decisions in localized high-risk disease.

# Abstract: Eosinophils in Clear-Cell Renal Cell Carcinoma

**AUTHORS**

Marie Gilon, Department of Medical Oncology, University Hospital of Liège, Liège, Belgium

Claire Josse, Laboratory of Medical Oncology, GIGA-Research, University Hospital of Liège, Liège, Belgium

Delphine Boulet, Laboratory of Medical Oncology, GIGA-Research, University Hospital of Liège, Liège, Belgium

Nancy Detrembleur, Department of Anatomical Pathology, University Hospital of Liège, Liège, Belgium

Vincent Bours, Department of Medical Genetics, University Hospital of Liège, Liège, Belgium

David Waltregny, Department of Urology, University Hospital of Liège, Liège, Belgium

Jean-Pascal Machiels, Department of Medical Oncology, Cliniques Universitaires Saint-Luc, Brussels, Belgium

Guy Jerusalem, Department of Medical Oncology, University Hospital of Liège, Liège, Belgium

Christine Gennigens, Department of Medical Oncology, University Hospital of Liège, Liège, Belgium

Eosinophils (EOS), mainly known for their role in allergic and anti-parasitic responses, have recently emerged as potential players in cancer immunity. This study investigated their impact in clear-cell renal cell carcinoma (ccRCC) through a retrospective study and an in vivo model. We retrospectively analyzed data from 169 patients with localized ccRCC. Collected data included clinical and anatomopathological characteristics, survival outcomes, as well as hematological values at diagnosis, annually up to five years, and at progression. Tissue EOS infiltration was quantified in 96 patients using Major Basic Protein staining across three regions: tumor center, invasive margin (1mm-wide region centered on the tumor-healthy tissue border), and peritumoral tissue. To produce an in vivo model, murine ccRCC cells were implanted in mouse kidneys, with one group receiving anti-interleukin-5 injections to decrease blood EOS and a control group. Higher baseline relative and absolute blood EOS counts were significantly associated with longer distant relapse-free survival (dRFS; p = 0.006 and 0.017), but not with cancer-specific survival (p = 0.97 and 0.71). Tissue EOS infiltration predominated in tumor periphery compared to tumor center, being mainly concentrated in the invasive margin (p = 0.016). When corrected for nuclear grade, higher tissue EOS infiltration in the invasive margin was associated with longer dRFS (HR = 0.69, p = 0.0299). In the in vivo model, a consistent decline in EOS count was observed in the anti-IL5 group compared to the control (p<0.0001), while leukocyte levels remained stable (p=0.605). Primary tumor growth was similar between groups (p=0.44), but lung metastases were significantly higher in the anti-IL5 group (p=0.0008). These results highlight eosinophils' potential as accessible, cost-effective biomarkers for risk stratification in localized ccRCC. In vivo experiments suggest a causal role, as eosinophil depletion increased metastatic dissemination. Further research is needed to elucidate eosinophil-tumor interactions and their clinical relevance.

# Abstract: Multicenter Randomized Trial of Deferred Cytoreductive Nephrectomy in Synchronous Metastatic Renal Cell Carcinoma Receiving Checkpoint Inhibitors: Nordic Sun Trial

**AUTHORS**

Niels Fristrup, Aarhus University Hospital and Aarhus University, Aarhus, Denmark

Lars Lund, Odense University Hospital and University of Southern Denmark, Odense, Denmark

Christian Beisland, Haukeland Universitetssjukehus, Bergen, Norway

Axel Bex, Royal Free London NHS Foundation Trust, London, UK

Børje Ljungberg, Umeå University, Umeå, Sweden

Frede Donskov, University of Southern Denmark, Esbjerg, Denmark

Anne Kirstine Møller Darras, Herlev University Hospital, Copenhagen, Denmark

Jesper Palshof, Herlev University Hospital, Copenhagen, Denmark

Jon Roikjær Henriksen, Odense University Hospital and University of Southern Denmark, Odense, Denmark

Niels Viggo Jensen, Odense University Hospital and University of Southern Denmark, Odense, Denmark

Tommy Kjærgaard Nielsen, Aalborg University Hospital and Aalborg University, Aalborg, Denmark

Anders Kjellman, Karolinska Universitetssjukhuset, Stockholm, Sweden

Per-Olof Lundgren, Karolinska Universitetssjukhuset, Stockholm, Sweden

Magnus Lindskog, Karolinska Universitetssjukhuset, Stockholm, Sweden

Katriina Jalkanen, Helsinki University Hospital, Helsinki, Finland

Lars Dyrskjøt, Aarhus University Hospital, Aarhus, Denmark

Sarah Grønbech Steen, Aarhus University Hospital, Aarhus, Denmark

Ane Iversen, Aarhus University Hospital, Aarhus, Denmark

Mads Agerbæk, Aarhus University Hospital, Aarhus, Denmark

Iben Lyskjær, Aarhus University Hospital, Aarhus, Denmark

Background: Primary tumor removal by cytoreductive nephrectomy (CN) in synchronous metastatic renal cell carcinoma patients has been investigated in the context of various treatment regimens. In the era of targeted tyrosine kinase inhibitor therapy two randomized trials, CARMENA and SURTIME, have questioned the role and timing of CN with results pointing towards an improved benefit upon a deferred approach; except for patients with >3 IMDC risk factors. The deferred CN approach ensures systemic therapy for all patients, avoids systemic treatment delay, and spares surgery in patients with progressive tumors. Here we evaluate deferred cytoreductive nefrectomy in the era of immunotherapy.

Methods: This is an open, randomized, multicenter comparison trial, designed to evaluate the effect of deferred cytoreductive nephrectomy compared with no surgery following initial nivolumab/ipilimumnab or a TKI-IO combination, in mRCC patients with IMDC intermediate and poor risk. Patients are recruited from in all four Nordic countries (Denmark, Norway, Sweden and Finland) with an intended full accrural in 2028. The trial aims to show that deferred cytoreductive nephrectomy improves overall survival in patients with synchronous metastatic renal cell carcinoma treated with immunotherapy. NORDIC SUN furthermore integrates a comprehensive translational research program with tissue, blood, and microbiome sampling for biomarker analysis. Currently, 175 out of 400 patients are enrolled. Clinical Trial Registry Number: NCT03977571.



# Abstract: Optimization of comprehensive total transcriptomic profiling in renal cell carcinomas

**AUTHORS**

Radka Vaclavikova, Laboratory of Pharmacogenomics, Biomedical Center, Faculty of Medicine in Pilsen, Charles University, Pilsen, Czech Republic

Karolina Seborova, Toxicogenomics Unit, National Institute of Public Health, Prague, Czech Republic

Kamila Koucka, Toxicogenomics Unit, National Institute of Public Health, Prague, Czech Republic

Tereza Tesarova, Toxicogenomics Unit, National Institute of Public Health, Prague, Czech Republic

Pavel Soucek, Toxicogenomics Unit, National Institute of Public Health, Prague, Czech Republic

Milan Hora, Department of Urology, Faculty of Medicine in Pilsen and University Hospital, Charles University, Pilsen, Czech Republic

Kristyna Pivovarcikova, Department of Pathology, Faculty of Medicine in Pilsen and University Hospital, Charles University, Pilsen, Czech Republic

Ondrej Fiala, Department of Oncology and Radiotherapeutics, Faculty of Medicine in Pilsen and University Hospital, Charles University, Pilsen, Czech Republic

Total RNA sequencing (total RNA-Seq) provides complete characterization of human transcriptome. Whereas mRNA-sequencing captures only polyadenylated transcripts, total RNA-Seq encompasses RNA species, thereby enabling a more comprehensive assessment of gene expression and transcriptomic complexity. This expanded analytical scope is relevant for general oncology and especially for renal cell carcinomas (RCC), where non-coding RNAs contribute substantially to tumor biology and disease progression. We focused on optimization of total transcriptomic profiling beyond protein-coding regions by implementing total RNA-Seq in ccRCC tumor samples. Three ribodepletion-based library preparation kits were evaluated (3 samples per kit): Lexogen’s Total RNA-Seq Library Prep Kit with RiboCop rRNA Depletion Kit V1.2, Kapa Biosystems’ KAPA RNA HyperPrep Kit with RiboErase (HMR), and New England BioLabs’ NEBNext Ultra II RNA Library Prep Kit with rRNA Depletion Kit (HMR). Library quality and quantity was assessed using the High Sensitivity DNA Kit. Libraries were sequenced on Illumina NextSeq 2000 platform (paired-end, 2×150bp, targeting 16 million reads per sample). Final kit selection was guided by quality control metrics derived from FastQC, alignment performance evaluated using the RSeQC package, and mainly by the efficiency of depletion estimated by sortMeRNA package. The best ribodepletion performance had RiboCop rRNA Depletion Kit V1.2 from Lexogen (~1% rRNA after depletion). Accordingly, except better performance of Lexogen and Kapa Biosystems kits in the ribodepletion step, they also outperformed the New England Biolabs kit in alignment statistics (81% vs. 85% and 89%) and provide our future solution for total transcriptome characterization. Supported by the Czech Health Research Council under project No. NW25-03-00122, by the Charles University Research Fund (Cooperatio program No. 207043 – Surgical disciplines), and by the project „Integration of biomedical research and health care in the Pilsen metropolitan area“; reg. no. CZ.02.01.01/00/23_021/0008828) - co-funded by the European Union and by the State Budget of the Czech Republic.

# Abstract: Left Radical Nephrectomy for Renal Cell Carcinoma with Tumor Thrombus extending into Supradiaphragmatic Inferior Vena Cava Without the Use of cardiopulmonary Bypass, Veno-venous Bypass, or Deep Hypothermic Circulatory Arrest

**AUTHORS**

Nikolaos Gianniris, Evaggelismos General Hospital, Department of Urology, Athens, Greece

Charalambos Kypraios, Evaggelismos General Hospital, Department of Urology, Athens, Greece

Triantafillos Papadopoulos, Evaggelismos General Hospital, Department of Urology, Athens, Greece

Apostolis Athanasiou, Evaggelismos General Hospital, Department of Urology, Athens, Greece

Ioannis Xoxakos, Evaggelismos General Hospital, Department of Urology, Athens, Greece

Dimitrious Karamanolakis, Evaggelismos General Hospital, Department of Urology, Athens, Greece

Dimitrious Chasiotis, Evaggelismos General Hospital, 2nd General Surgery Department, Athens, Greece

Ioannis Efthimiou, Evaggelismos General Hospital, Department of Urology, Athens, Greece

INTRODUCTION & OBJECTIVES: The surgical treatment of renal cancer (RCC) with extension to the inferior vena cava (IVC) is uncommon. The presence of a tumor thrombus is not a negative factor for patient survival; only a poor general condition of the patient or metastatic disease reduces survival in these patients. We present a case report where neither cardiopulmonary bypass nor hypothermia was required.

CASE REPORT: A 68-year-old patient with an 8 cm tumor of the left kidney with extension of a tumor thrombus IIIb, platelet thrombus extending to the common iliac veins (Fig.), and a recent episode of pulmonary embolism underwent complete resection transabdominally via a Mercedes-type incision with mobilization of the liver and peritoneum of the upper abdomen. The IVC was exposed sub-hepatically and above the hepatic veins, with mobilization of the diaphragm and ligation of the phrenic vessels. This was followed by splenectomy and radical nephrectomy. The IVC tumor within the mediastinum was mobile and could be manually milked down to allow placement of vascular forceps. Pringle’s maneuver and vascular clamps were placed in the standard order to allow IVC incision for the removal of platelet and tumor thrombus. Operative time was 6 hours and 20 minutes. Blood loss was 500 mL The patient remained in the ICU for 24 hours and was discharged home on the 15th postoperative day with low molecular weight heparin. The pathology report showed RCC of conventional type, nuclear grade 2 & 3, stage pT3a with negative surgical margins. He referred to our oncology colleagues for adjuvant immunotherapy. The patient is disease-free at 24 months.

CONCLUSION: Radical nephrectomy with IVC tumor level IIIb is feasible with an abdominal and transdiaphragmatic approach.



# Abstract: Refining frailty-based patient selection for same-day robot-assisted radical nephrectomy

**AUTHORS**

Anne-Sofie Vibæk Eisum, Aalborg University Hospital, Department of Renal Surgery and Urology, Aalborg, Denmark

Theresa Junker, Odense University Hospital, Department of Urology, Odense, Denmark

Clara Bender, Aalborg University, Department of Health Science and Technology, Aalborg, Denmark

Lone Jørgensen, Aalborg University Hospital, Department of Clinical Nursing and Clinical Medicine, Aalborg, Denmark

Pia Danstrup-Dinesen, Aalborg University Hospital, Department of Anaesthesia and Intensive Care Unit, Aalborg, Denmark

Hayder Al-Husseinawi, Aalborg University Hospital, Department of Renal Surgery and Urology, Aalborg, Denmark

Tommy Kjærgaard Nielsen, Aalborg University Hospital, Department of Renal Surgery and Urology, Aalborg, Denmark

Introduction: Robot-assisted radical nephrectomy (RARN) is a well-established treatment for localised kidney cancer. Implementation of same-day surgery requires careful identification of patients at risk of postoperative complications after early discharge, as traditional perioperative risk stratification may insufficiently capture individual physiological reserve. This study aims to refine frailty assessment to optimise patient selection for safe same-day RARN. Material and

Methods: ROB’N’SAFE is an ongoing prospective study comprising three sub-studies. This abstract focuses on Study I which examines preoperative frailty assessment in relation to same-day surgery.

Study I: A prospective observational cohort study assessing preoperative frailty markers and feasibility of same-day discharge following RARN. Variables include Clinical Frailty Scale, physical performance tests (handgrip strength and chair-stand test), CT-derived body composition data, and home-based wearable-derived biometric data. The primary outcome is successful same-day discharge without complications or readmission within 72 hours. Secondary outcomes include 30-day complications, readmission, health literacy, and patient-reported quality of life.

Preliminary results: To date, 13 patients have been included. Median age was 68 years (range 30–84), and 77% were male. Same-day discharge was achieved in 9 patients (69%). Median Clinical Frailty Scale was 2 (range 1–3), and preoperative frailty measures demonstrated heterogeneity within the cohort. No patients experienced complications requiring hospital admission or readmission within 72 hours. Wearable-derived activity data and CT imaging-derived biomarkers are currently under evaluation.

Conclusion: These preliminary findings support the feasibility of same-day RARN in carefully selected patients. Ongoing integration of multimodal frailty measures may further improve precision in patient selection and optimise safety in same-day kidney cancer surgery. By combining clinic-based frailty screening with home-based monitoring and imaging-derived biomarkers, this study aims to provide a more nuanced assessment of physiological reserve to support safe same-day surgery.

# Abstract: Preoperative prediction of cancer-specific and other-cause mortality after surgery for nonmetastatic renal cell carcinoma

**AUTHORS**

Philip Hedegaard, Department of Urology, Copenhagen University Hospital – Zealand University Hospital Roskilde, Roskilde, Denmark

Marie Juul-Haslund, Department of Urology, Copenhagen University Hospital - Herlev and Gentofte, Herlev, Denmark

Rasmus D. Petersson, Department of Urology, Copenhagen University Hospital – Zealand University Hospital Roskilde, Roskilde, Denmark

Emma Heeno, Department of Urology, Aarhus University Hospital, Aarhus, Denmark

Rasmine Bak, Department of Urology, Odense University Hospital, Odense, Denmark

Lars Lund, Department of Urology, Odense University Hospital, Odense, Denmark

Frederik F. Thomsen, Department of Urology, Copenhagen University Hospital – Zealand University Hospital Roskilde, Roskilde, Denmark

Background: Accurate preoperative risk assessment is essential for shared decision-making in patients with non-metastatic renal cell carcinoma (nmRCC), particularly in the presence of competing mortality risks. We assessed and internally validated a preoperative model to predict 10-year cancer-specific mortality (CSM) and other-cause mortality (OCM) after surgery for nmRCC.

Methods: Danish patients undergoing surgery for nmRCC between 2010 and 2018 were identified in the DaRenCa Study-3 and divided into training and validation cohorts. A competing risk model was developed, assessing associations between preoperative covariates and CSM using a Fine-Gray model, with death from other causes as a competing event. A separate model was constructed for OCM.

Results: 2,633 patients were included, with a median follow-up of 7.1 years. For cancer-specific mortality, three covariates were included in the final model: tumor size (categorized according to the AJCC pT classification), age, and presence of symptoms at diagnosis. For other-cause mortality, the model included smoking status (current, former, never), age modelled with natural splines, and ECOG/WHO performance status (0 vs. ≥1). As an example, a 70-year-old patient diagnosed with an asymptomatic 4 cm tumor had an estimated 10-year CSM risk of 4%. The same patient would have an estimated 10-year OCM risk of 28% if they were a current smoker with a performance status of 0. The model showed strong discrimination in the validation cohort (10-year AUC: 0.74 for both CSM and OCM models). Calibration demonstrated good agreement between predicted and observed risks in the low-to-intermediate risk ranges, particularly for CSM.

Conclusion: This preoperative risk prediction model provides survival estimates that can support individualized treatment and shared decision-making.



# Abstract: Surgical Management of Epithelioid Angiomyolipoma with Inferior Vena Cava Invasion

**AUTHORS**

Rikke Vilsbøll Milling, Aarhus University Hospital, Department of Urology, Aarhus, Denmark

Sofie Møller Graff, Aarhus University Hospital, Department of Pathology, Aarhus, Denmark

Anna Krarup Keller, Aarhus University Hospital, Department of Urology, Aarhus, Denmark

Mia Gebauer Madsen, Aarhus University Hospital, Department of Urology, Aarhus, Denmark

Epithelioid angiomyolipoma (eAML) is a rare variant of angiomyolipoma, defined by the presence of more than 80% epithelioid cells. While angiomyolipoma is traditionally regarded as a benign mesenchymal renal tumor, eAML carries malignant potential and may demonstrate aggressive behavior. A woman in her 40s was referred to our department from another urological unit for evaluation of surgical eligibility following biopsy-confirmed eAML of the right kidney with a 2 cm inferior vena cava tumour thrombus. A PET–CT scan demonstrated no evidence of metastatic disease. The case was reviewed at a multidisciplinary team conference and was deemed suitable for surgical intervention. An open radical nephrectomy with IVC thrombectomy was performed. The kidney and renal hilum were exposed through a transperitoneal approach. The medial hepatic ligament was divided, and adjacent organs were mobilized to allow adequate exposure of the kidney and renal vessels. The right renal artery was identified between the inferior vena cava and the aorta, isolated, ligated, and divided. The inferior vena cava was subsequently exposed and controlled both cranially and caudally to the right renal vein. Vena cava was opened adjacent to the right renal vein, allowing removal of the tumour thrombus, after which the vena cava was closed using a 5-0 suture. The kidney was mobilized and removed, followed by careful haemostasis and layered closure of the abdominal wall. Histopathological examination confirmed eAML with negative surgical margins. Postoperatively, the patient experienced pain as the only complication and was discharged on postoperative day six. Only a few cases of eAML with inferior vena cava invasion have been reported. In the present case, the primary diagnostic workup was performed at another institution. We do not recommend preoperative biopsy, as it may delay definitive surgical treatment. Due to its malignant potential, eAML should be managed as renal cell carcinoma.

# Abstract: CT-Guided Cryoablation of T1a Renal Cell Carcinoma: Technical efficacy and oncological outcomes from a Single-Center

**AUTHORS**

Simone Buchardt Brandt, Department of Urology, Aarhus University Hospital, Aarhus, Denmark

Theresa Junker, Department of Urology, Odense University Hospital, Odense, Denmark

Casper Riis Therkildsen, Department of Urology, Aarhus University Hospital, Aarhus, Denmark

Nina Juul Brønnum, Department of Urology, Aarhus University Hospital, Aarhus, Denmark

Jens Borgbjerg, Department of Radiology, Akershus University Hospital, Lørenskog, Norway

Nis Elbrønd Larsen, Department of Radiology, Aarhus University Hospital, Aarhus, Denmark

Gratien Andersen, Department of Radiology, Aarhus University Hospital, Aarhus, Denmark

Ole Graumann, Department of Radiology, Aarhus University Hospital, Aarhus, Denmark

Mia Gebauer Madsen, Department of Urology, Aarhus University Hospital, Aarhus, Denmark

Background: Percutaneous CT-guided cryoablation (PCA) has emerged as a minimally invasive alternative to partial nephrectomy for T1a renal cell carcinoma (RCC), potentially preserving renal function while achieving favorable oncological outcomes. However, large-scale evidence on long-term efficacy remains limited. Methods: We conducted a retrospective analysis of all patients undergoing primary PCA for biopsy-verified T1aN0M0 RCC at a single center from 2015-2022. Exclusion criteria included previous RCC, hereditary kidney cancer syndromes, or insufficient follow-up. Primary technical efficacy was defined as complete ablation on first follow-up imaging; secondary efficacy included successful re-cryoablation. Oncological outcomes were monitored through regular imaging and laboratory follow-up. Local progression was defined as nodular enhancement in the ablation cavity on imaging, with no residual tumor on prior scans, more than three months after PCA. Results: A total of 472 patients underwent primary PCA with median follow-up of 4 years. Median age at treatment was 67 years, and 69% had ASA classification of I-II. Primary technical efficacy was 98.7%. Treatment of incomplete PCA included repeat PCA (n=3), active surveillance (n=2), and surgical resection (n=1). All repeat PCA procedures were successful, resulting in a secondary technical efficacy of 99.4% (469/472). Local progression occurred in 1.3% of patients at a median of 417 days post-treatment; all cases were successfully treated with recryoablation. Metastases developed in 1.3% of patients. Conclusion: In this study percutaneous CT-guided cryoablation had a high technical success, durable local control and low metastasis rates in patients with T1a RCC. These findings suggest that cryoablation is a viable nephron-sparing treatment option for appropriately selected patients.

# Abstract: Renal cell carcinoma metastasis in a contralateral ureter

**AUTHORS**

Theodore Poulton, Royal Devon and Exeter Hospital, Exeter, Devon, UK

Aryaan Majumdar, University of Exeter, Exeter, Devon, UK Tom Minto, Royal Devon and Exeter Hospital, Exeter, Devon, UK

Sarah Welsh, Royal Devon and Exeter Hospital, Exeter, Devon, UK

Nicholas Campain, Royal Devon and Exeter Hospital, Exeter, Devon, UK

A 73 year old male patient with a history of right radical nephrectomy for clear cell renal cell carcinoma (ccRCC) eight years prior presented acutely with visible haematuria, left loin pain, and reduced renal function. Non-contrast CT demonstrated left-sided hydronephrosis secondary to a 2.5cm distal left ureteric lesion. Due to severely impaired renal function, urgent ureteric stenting was performed, with diagnostic ureteroscopy and biopsy. Subsequent CT urogram demonstrated radiological features suspicious of ureteric transitional cell carcinoma, however ureteroscopic biopsies confirmed metastatic clear cell RCC. Ureteric recurrence of RCC have been described, but more frequently occur in the ipsilateral ureteric stump and typically within shorter timeframes from initial nephrectomy. Following multidisciplinary team discussion, the patient underwent a left robot-assisted distal ureterectomy and reimplantation, driven by the need to preserve renal function, avoid lifelong ureteric stenting and in the absence of other sites of metastatic disease. The response of an isolated intraluminal ureteric metastases to systemic therapy is unknown. It was postulated that deferring local treatment risk may risk ureteric obstruction if there was local progression of the metastatic deposit. Post-operative histology confirmed clear cell RCC with clear margins. Staging CT identified two stable small pulmonary nodules. Two months later, biopsy of a superficial chest wall lesion confirmed another ccRCC metastasis. Systemic therapy with axitinib and avelumab was planned. Repeat staging demonstrated disease progression, with interval increase in pulmonary nodules and new pancreatic and metastases. This case represents an exceptionally rare case of late RCC metastasis in a contralateral ureter 8 years post-radical nephrectomy. Surgical resection achieved local disease control and renal preservation, negating the need for lifelong ureteric stenting. Subsequent systemic progression highlights the challenges in timing and sequencing of local and systemic treatment for recurrent oligometastatic disease.

# Abstract: The management of renal manifestations in Von Hippel–Lindau Syndrome: Insights from a single institution’s three-decade experience

**AUTHORS**

Selcuk Erdem, Istanbul University Istanbul Faculty of Medicine, Division of Urologic Oncology, Department of Urology, Istanbul, Turkey

Firat Ozervarli, Istanbul University Istanbul Faculty of Medicine, Department of Urology, Istanbul, Turkey

Melih Bicer, Istanbul University Istanbul Faculty of Medicine, Department of Urology, Istanbul, Turkey

Yasin Ates, Istanbul University Istanbul Faculty of Medicine, Department of Urology, Istanbul, Turkey

Ozge Hurdogan, Istanbul University Istanbul Faculty of Medicine, Department of Pathology, Istanbul, Turkey

Tugba Kalayci, Istanbul University Istanbul Faculty of Medicine, Department of Medical Genetics, Istanbul, Turkey

Zehra Oya Uyguner, Istanbul University Istanbul Faculty of Medicine, Department of Medical Genetics, Istanbul, Turkey

Yasemin Ozluk, Istanbul University Istanbul Faculty of Medicine, Department of Pathology, Istanbul, Turkey

Oner Sanli, Istanbul University Istanbul Faculty of Medicine, Division of Urologic Oncology, Department of Urology, Istanbul, Turkey

Faruk Ozcan, Istanbul University Istanbul Faculty of Medicine, Division of Urologic Oncology, Department of Urology, Istanbul, Turkey

Introduction&Objectives: Von Hippel–Lindau (VHL) Syndrome is characterized by genetic based, early-onset, multifocal and/or bilateral renal cysts and tumors. This study presents a tertiary institution's three-decade experience in managing renal manifestations in VHL Syndrome. Materials&Methods: Between 1995-2025, 51 patients with VHL syndrome were surveilled and/or treated for renal cyst/tumor at our institution. Surgical or interventional treatment was indicated for tumors ≥3 cm, while smaller cysts/tumors were managed with active surveillance(AS). Demographico-clinical, histopathological, renal functional and survival outcomes were retrospectively analyzed. Results: The median age was 29 years at VHL diagnosis and 44 years at first renal surgery. Forty-two patients (82.4%) had hereditary and nine (17.6%) sporadic VHL. AS was the initial management in 27 patients (52.9%), of whom 16 (31.4%) remained in AS with median 111 months. One patient (2%) received belzutifan. Overall, 35 patients (68.6%) had at least one renal intervention, and 11 (21.6%) had prior AS with median 32 months surveillance, whereas 24 (47.1%) underwent intervention directly. Total intervention number was 71, including 46 (64.8%) partial nephrectomies, 22 (30.9%) radical nephrectomies, and 3 (4.2%) cryoablations. A total of 189 renal tumors and 96 cysts were removed. After 109 months follow-up, 16 (45.7%) patients progressed, 13 (37.1%) with denovo renal tumor and 3(8.6%) with distant metastases. Fifteen-year progression-and distant metastasis-free survival were 36.3% and 93.1%, respectively. Five patients (9.8%) required renal replacement therapy (three dialysis, two transplantation). In 46 patients, median eGFR decreased from 92.1 to 72.1 mL/min/1.73 m^2^. Fifteen-year overall survival was comparable between AS and surgery groups (83.9% vs 69.0%, p=0.523). Conclusions: This long-term single institution experience confirms that 3 cm rule is safe and effective strategy for decision making on AS, surgical or interventional treatments to preserve renal function while maintaining durable cancer control.

# Abstract: Outcomes for patients with metastatic chromophobe renal cell carcinoma treated with systemic therapies: a large multicentre retrospective study

**AUTHORS**

Nadine Mansour, Department of Medical Oncology, Hôpital Européen Georges-Pompidou, Université Paris Cité, Paris, France

Edouard Auclin, Department of Medical Oncology, Gustave Roussy, Paris Saclay University, Villejuif, France

Ronan Flippot, Department of Medical Oncology and Hepato-Gastroenterology, Hospices Civils de Lyon, Lyon, France

Mathilde Vincens, Department of Medical Oncology, CRCM, Aix-Marseille University, CNRS, INSERM, Marseille, France

Mathilde Beaufils, Department of Medical Oncology, Institut Català d'Oncologia (ICO) - Hospital Universitari Bellvitge - Institut d'Investigació Biomèdica de Bellvitge, L'Hospitalet de Llobregat, Barcelona, Spain

Javier Gavira, Department of Medical Oncology, Bordeaux University Hospital, Bordeaux, France

Marine Gross-Goupil, Department of Medical Oncology, Centre Antoine Lacassagne, Université Côte d'Azur, Nice, France

Delphine Borchiellini, Department of Medical Oncology, Centre Jean Perrin, Clermont-Ferrand, France

Hakim Mahammedi, Laboratory of Experimental Oncology, KU Leuven, Leuven, Belgium; Department of General Medical Oncology, University Hospitals Leuven, Leuven Cancer Institute, Leuven, Belgium

Benoit Beuselinck, Department of Medical Oncology, Centre Eugène Marquis, Rennes, France

Brigitte Laguerre, Centre François Baclesse, Caen University Hospital, Caen, France

Romain Levard, Department of Medical Oncology, Henri Mondor Hospital, Créteil, France

Charlotte Joly, Department of Medical Oncology, Pitié-Salpêtrière Hospital, AP-HP, Paris, France

Loic Jaffrelot, Department of Medical Oncology, Saint-Louis Hospital, AP-HP, Université Paris Cité, Paris, France

Clement Dumont, Department of Medical Oncology, Tours University Hospital, Tours, France

Mathilde Cancel, Department of Urology, Bichat Claude Bernard Hospital, AP-HP, Université Paris Cité, Paris, France

Idir Ouzaid, Department of Medical Oncology, Charles Nicolle University Hospital, Rouen, France

Maele Fontaine, Department of Urology, Toulouse University Hospital, Toulouse, France

Thomas Prudhomme, Department of Urology, Toulouse University Hospital, Toulouse, France

Constance Thibaut, Department of Medical Oncology, Hôpital Européen Georges-Pompidou, Université Paris Cité, Paris, France

Stéphane Oudard, Department of Medical Oncology, Hôpital Européen Georges-Pompidou, Université Paris Cité, Paris, France

Background: Chromophobe renal cell carcinoma (chRCC) accounts for ~5% of kidney cancers. There are no established treatment guidelines for metastatic chRCC. While immunotherapy (IO) and tyrosine kinase inhibitors (TKIs) show efficacy in metastatic clear cell renal cell carcinoma, data supporting their use in chRCC remain limited.

Patients and methods: We conducted a multicentre retrospective study of patients with advanced or metastatic chRCC treated with systemic therapy in 18 centres between 2013 and 2025. Patients were categorised by first-line treatment as TKI monotherapy or IO-based therapy (anti–PD-1 alone or combined with a TKI and/or anti–CTLA-4). The primary endpoint was objective response rate (ORR); secondary endpoints were progression-free survival (PFS), and overall survival (OS). Survival outcomes were estimated using Kaplan–Meier methods, and Cox models were used to assess prognostic factors.

Results: 120 patients were included (median age 61 years, 55% male, 28% with sarcomatoid features). First-line treatments were TKI monotherapy (74.2%) and IO-based therapy (25.8%). In the overall cohort, ORR was 28.2%, DCR was 74.5%, and median PFS and OS were 9.8 and 41 months, respectively. Compared with TKI monotherapy (median PFS 9.1 months; median OS 35.2 months), IO-based therapy showed numerically longer median PFS (14.2 months) and OS (41.7 months). Within the sarcomatoid subgroup, IO-based therapy was associated with numerically longer PFS (14.4 vs 4.4 months) and OS (20.1 vs 15.1 months) compared with TKI monotherapy. In multivariable Cox analysis, International Metastatic RCC Database Consortium poor risk status was an independent predictor of shorter PFS and OS.

Conclusion: IO-based regimens may provide greater benefit compared to single-agent TKI in chRCC, especially in sarcomatoid tumours. Prospective biomarker-driven studies are needed to optimise treatment selection.



# Abstract: Digital Monitoring of Self-Reported Symptoms in Patients Treated With Cabozantinib Plus Nivolumab for Advanced Clear-Cell Renal Cell Carcinoma: Protocol for the CANIQOL Multicentre Phase IV Study

**AUTHORS**

Romain Levard, Centre François Baclesse, Caen, France

Alexandra Leconte, Centre François Baclesse, Caen, France

François Gernier, Centre François Baclesse, Caen, France

Justine Lequesne, Centre François Baclesse, Caen, France

Charles Parnot, Cureety, Paris, France

François Christy, Centre François Baclesse, Caen, France

Bénédicte Clarisse, Centre François Baclesse, Caen, France

Florence Joly, Centre François Baclesse, Caen, France

Background: Cabozantinib plus nivolumab is a first-line standard of care for advanced clear-cell renal cell carcinoma (RCC) based on CheckMate 9ER. In routine practice, patients often differ from clinical trial populations, and clinician-reported adverse events underestimate symptomatic burden. Remote patient monitoring (RPM) using patient-reported outcomes (PROs) may improve early detection and management of toxicities and support adherence. CANIQOL was designed to assess the clinical utility of digital RPM in patients treated with cabozantinib–nivolumab in real-world settings. Methods: CANIQOL is a prospective, multicentre, single-arm, real-world phase IV study conducted in France. Adults initiating cabozantinib (40 mg once daily) plus nivolumab (240 mg every 2 weeks or 480 mg every 4 weeks) are enrolled. Patients complete weekly PROCTCAE assessments for 6 months via the Cureety platform, generating automated alerts prompting clinical actions. Additional instruments include FACIT-F, FKSI-10, HADS, the Girerd adherence scale, and the French System Usability Scale. The primary endpoint is the proportion of patients with at least one RPM-triggered treatment-management adjustment within 3 months. Secondary endpoints include symptom trajectories, fatigue, quality of life, anxiety/depression, adherence, satisfaction, treatment duration, RECIST 1.1 outcomes, and IMDC subgroup analyses. Eighty-three patients will be enrolled to obtain 70 evaluable participants. First inclusion occurred in October 2025. Conclusion: CANIQOL will provide real-world evidence on the feasibility and clinical relevance of high-frequency PRO-based monitoring to optimize toxicity management, adherence, and patient experience during first-line cabozantinib–nivolumab for advanced RCC.

# Abstract: Assessing Patient Decision Regret Following Partial Nephrectomy for Benign Renal Masses: A Patient-Reported Outcomes Study

**AUTHORS**

Sarika Nalagatla, Frimley Renal Cancer Centre, Frimley Park Hospital, Frimley, UK

Maria Yacoub, University College London, Division of Surgery and Interventional Science, London, UK

Aqua Asif, Frimley Renal Cancer Centre, Frimley Park Hospital, Frimley, UK

Samuel Davis, Frimley Renal Cancer Centre, Frimley Park Hospital, Frimley, UK

Manar Malki, Frimley Renal Cancer Centre, Frimley Park Hospital, Frimley, UK

Muddassar Hussain, Frimley Renal Cancer Centre, Frimley Park Hospital, Frimley, UK

Neil Barber, Frimley Renal Cancer Centre, Frimley Park Hospital, Frimley, UK

Background: Percutaneous renal mass biopsy (RMB) provides histological confirmation for indeterminate renal lesions, yet its routine use before robot-assisted laparoscopic partial nephrectomy (RALPN) remains controversial. Approximately 30% of surgically excised small renal masses (SRMs <4cm) are benign. While RALPN offers excellent oncological control and renal preservation, it carries peri-operative risks. Decision regret, a recognised patient-reported outcome in urological oncology, reflects dissatisfaction with a healthcare choice. This study assessed the prevalence and determinants of decision regret in patients undergoing RALPN without prior RMB who were subsequently found to have benign histology, to inform shared decision-making (SDM). Methods: A mixed-methods study combined retrospective clinical data with prospective patient-reported outcomes. Patients undergoing RALPN for SRMs ≤4cm (January 2010-July 2025) without RMB and with benign histology in a high-volume tertiary referral centre were included. A standardised questionnaire incorporating the validated five-item Decision Regret Scale (DRS) was distributed by post, electronically or by telephone. Significant regret was defined as DRS ≥25. Free-text responses underwent thematic analysis. Results: Of 1,480 RALPNs performed, 158 patients met inclusion criteria; 58/106 consenting participants responded. Median age was 66 years and median tumour size was 30mm. No Clavien-Dindo ≥III complications occurred. Respondents were predominantly White (94.8%) with mixed educational attainment. Most recalled being informed the mass could be benign (79.3%). RMB discussion was recalled by 55.2%, while 29.3% reported no discussion and 15.5% could not recall. Mean DRS was 13.8 (95% CI 10.3-17.4); 27.6% reported DRS ≥25. Despite benign histology, 87.9% felt surgery was the right decision and only 29.3% would have preferred biopsy retrospectively. Regret was lowest among those recalling detailed RMB discussion and rating information as excellent. Conclusions: Decision regret after RALPN for benign pathology was low. Higher information quality and detailed RMB counselling correlated with lower regret, supporting structured SDM and explicit pre-operative discussion of RMB.

# Abstract: Exploring the clinical predictors of worse disease-free survival (DFS) among patients with non-metastatic (M0) resected clear-cell renal cell carcinoma (ccRCC): insights for future neoadjuvant clinical trial design

**AUTHORS**

Salvatore Granata, AOU Careggi, Unit of Urology and Renal Transplantation, Florence, Italy

Antonio Crescente, AOU Careggi, Unit of Urology and Renal Transplantation, Florence, Italy

Alessio Pecoraro, AOU Careggi, Unit of Urology and Renal Transplantation, Florence, Italy

Francesco Sessa, University of Florence, Department of Experimental and Clinical Medicine, Florence, Italy

Giulia Bocu, AOU Careggi, Unit of Urology and Renal Transplantation, Florence, Italy

Edoardo Dibilio, AOU Careggi, Unit of Urology and Renal Transplantation, Florence, Italy

Sergio Serni, AOU Careggi, Unit of Urology and Renal Transplantation, Florence, Italy

Lorenzo Masieri, AOU Careggi, Unit of Urology and Renal Transplantation, Florence, Italy

Riccardo Campi, AOU Careggi, Unit of Urology and Renal Transplantation, Florence, Italy

Neoadjuvant/perioperative immunotherapy is emerging as a promising strategy for patients with high-risk resectable RCC, mirroring other tumor types. One key limitation of available neoadjuvant trials is heterogeneous inclusion criteria; arguably, patients who may benefit the most from neoadjuvant/perioperative treatment are those with poorer prognosis. To provide insights on future clinical trial design, we evaluated the impact of three prognostic scores (KEYNOTE-564 criteria, Leibovich 2003, and UroPredict) on DFS in patients with resected M0 ccRCC, exploring the clinical predictors of their worst prognostic category. We queried our prospective institutional RCC database to select patients with resected M0ccRCC between 01/2017-05/2024. DFS (per Keynote-564) was estimated using the Kaplan–Meier method. A Cox proportional hazards regression model was used to calculate HRs with 95%CIs. Multivariable logistic regression analysis evaluated the clinical predictors of the worst prognostic category. 518 patients were included (median age 67y; 70%males; median preoperative eGFR 81ml/min; median tumour diameter 3.5cm; partial nephrectomy 74%). 29% met the KEYNOTE-564 criteria, 12% had a Leibovich high-risk, 13% had a UroPredict high-risk. The median follow-up was 24 months (IQR 9.5–48.5). DFS was significantly lower in patients meeting the KEYNOTE-564 criteria (HR 2.93, 95% CI: 1.79–4.80), in patients with Leibovich high-risk (HR: 4.24, 95% CI: 2.45–7.35), and in patients with UroPredict high-risk (HR: 4.58, 95% CI: 2.74–7.65). At multivariable analysis, cT stage (cT3-4/cT1-2) and increasing anatomic tumour complexity were independent predictors of each score's worst prognostic group (Figure). In patients with M0 resectable (biopsy-proven) ccRCC, non-organ confined and anatomically complex tumors at preoperative imaging carry a higher risk of the worst pathological prognostic score categories and worse DFS. These patients are theoretically those who may benefit the most from neoadjuvant/perioperative therapy and therefore the ideal candidates for future clinical trials.



# Abstract: Mapping the Cancer Landscape of BAP1 Tumor Predisposition Syndrome in a Surveillance Cohort

**AUTHORS**

Freja Hansen, Department of Urology, Aarhus University Hospital, Aarhus, Denmark

Kirstine Stochholm, Department of Endocrinology and Internal Medicine, Aarhus University Hospital, Aarhus, Denmark

Lise Graversen, Department of Clinical Genetics, Aarhus University Hospital, Aarhus, Denmark

Mia Gebauer Madsen, Department of Urology and Department of Clinical Medicine, Aarhus University Hospital, Aarhus, Denmark

Background: Pathogenic variants in the tumor suppressor gene BAP1 are associated with an increased risk of developing a spectrum of malignancies, including uveal melanoma, malignant mesothelioma, cutaneous melanoma, renal cell carcinoma (RCC), basal cell carcinoma as well as benign skin lesions known as BAP1-inactivated melanocytic nevi. In addition, other tumors such as meningioma and cholangiocarcinoma have also been suggested to be associated with the syndrome. Methods: All patients enrolled in the BAP1 Tumor Predisposition Syndrome surveillance program at a single center were included. Clinical data including demographics, tumor type, treatment, follow-up information and other relevant clinical details were retrospectively collected from electronic patient records. Results: Thirty-three patients (17 women) with a median age of 41 years (range, 19-77 years) were included. Urological malignancies were observed in seven patients (21.2%), with a median age of 52 years. Among the urological malignancies, RCC was the most frequent diagnosis (n=5, 71.4%), with a median age at diagnosis of 42 years. Other urological malignancies included one case of urothelial carcinoma in situ in the bladder and one of malignant mesothelioma originating from tunica vaginalis testis. Of RCC cases, four were of clear cell type and one was unclassified. At the time of diagnosis, metastatic disease was present in a 27-year-old patient with RCC and in the patient with malignant mesothelioma. Beyond urological malignancies, patients presented with various types of cancer: basal cell carcinoma (n=14), malignant melanoma (n=4), malignant mesothelioma (n=3), uveal melanoma (n=1) and cholangiocarcinoma (n=1). Conclusion: The mapping of tumors in our BAP1 cohort underscores the necessity of a structured surveillance program, including urological monitoring, to ensure optimal treatment. Conflict of interest disclosure: The authors declare no conflict of interest.

# Abstract: Long-Term Remission of Metastatic Renal Cell Carcinoma Diagnosed During Pregnancy in a Patient with BAP1 Tumor Predisposition Syndrome

**AUTHORS**

Freja Hansen, Department of Urology, Aarhus University Hospital, Aarhus, Denmark

Kirstine Stochholm, Department of Endocrinology and Internal Medicine, Aarhus University Hospital, Aarhus, Denmark

Ane Bundsbæk Bøndergaard Iversen, Department of Oncology, Aarhus University Hospital, Aarhus, Denmark

Lise Graversen, Department of Clinical Genetics, Aarhus University Hospital, Aarhus, Denmark

Mia Gebauer Madsen, Department of Urology and Department of Clinical Medicine, Aarhus University Hospital, Aarhus, Denmark

Background: BAP1 Tumor Predisposition syndrome (BAP1-TPDS) is associated with increased risk of renal cell carcinoma (RCC), uveal melanoma, malignant mesothelioma, cutaneous melanoma and basal cell carcinoma, often with early onset. Case Report: A 27-year-old woman, 30 weeks’ gestation presented with sudden-onset macroscopic haematuria and left-sided flank pain. Initial differential diagnosis included nephrolithiasis and cystitis. Renal ultrasound revealed a solid mass in the upper pole of the left kidney. MRI confirmed a 7 cm renal tumor without vascular invasion. Pulmonary metastases were also detected (largest 1.3 cm) on the MRI. Due to aggressive disease, a planned caesarean section was performed at 33 weeks of gestation to allow initiation of oncological treatment. Three weeks postpartum, the patient underwent laparoscopic left-sided nephrectomy, including adrenalectomy and removal of 17 lymph nodes. Histopathological examination confirmed RCC of clear cell type, Fuhrman grade 3, pT3a, Leibovich score 6, no lymph node or adrenal metastasis. PET-CT revealed a reactive right bronchial hilar node, which was confirmed as metastatic RCC. The patient received four cycles of Nivolumab/Ipilimumab followed by 20 cycles of Nivolumab monotherapy over a two year-period. After 17 cycles, imaging showed complete response, however post-treatment CT-scan revealed a 16 mm lesion in the left diaphragmatic crus, consistent with metastatic RCC. The patient subsequently underwent stereotactic radiotherapy targeting the metastasis. She remains in remission for 5 years. Given the young age, genetic testing was performed and revealed BAP1-TPDS. Family history included multiple cases of mesothelioma. Conclusion: RCC in young patients may indicate hereditary kidney cancer syndromes, such as BAP1-TPDS. Multimodal treatment including surgery, immunotherapy and radiotherapy can achieve remission even in metastatic disease. Early genetic testing is important for surveillance, early detection and family counselling. Conflict of interest disclosure: The authors declare no conflict of interest.

# Abstract: Refining Risk-stratification in Patients with Non-metastatic Clear-cell Renal Cell Carcinoma meeting the KEYNOTE-564 Criteria using the Leibovich 2003 and UroPredict Prognostic Models: Implications for Shared Decision-making on Adjuvant Therapy

**AUTHORS**

Salvatore Granata, AOU Careggi, Unit of Urology and Renal Transplantation, Florence, Italy

Antonio Crescente, AOU Careggi, Unit of Urology and Renal Transplantation, Florence, Italy

Alessio Pecoraro, AOU Careggi, Unit of Urology and Renal Transplantation, Florence, Italy

Francesco Sessa, University of Florence, Department of Experimental and Clinical Medicine, Florence, Italy

Giulia Bocu, AOU Careggi, Unit of Urology and Renal Transplantation, Florence, Italy

David Ka-wai Leung, The Chinese University of Hong Kong, S. H. Urology Centre Department of Surgery, Hong Kong, Hong Kong

Sergio Serni, AOU Careggi, Unit of Urology and Renal Transplantation, Florence, Italy

Lorenzo Masieri, AOU Careggi, Unit of Urology and Renal Transplantation, Florence, Italy

Riccardo Campi, AOU Careggi, Unit of Urology and Renal Transplantation, Florence, Italy

Current Guidelines recommend offering adjuvant pembrolizumab to patients with resected clear cell renal cell carcinoma (ccRCC) meeting the KEYNOTE-564 criteria. The concept of using the Leibovich 2003 score - or similar scores - for selecting patients for adjuvant pembrolizumab (among patients meeting the KEYNOTE-564 criteria) has been proposed by a multidisciplinary European Delphi study. The aim of the study was to refine risk-stratification in patients meeting the KEYNOTE-564 criteria using the Leibovich 2003 and UroPredict scores. We selected patients who underwent surgery for M0ccRCC between 01-2017 and 05-2024 at a referral Academic Centre, who met the KN564criteria. Prognostic groups were reported according to the Leibovich and Uropredict subgroups (https://uropredict.sophiagenetics.com/?model=uropredict2). The primary outcome was disease-free survival (DFS). We investigated discrimination, calibration, net benefit, and prognostic value of both scores, focusing on the potential clinical implications of using them at specific cut-offs to guide patient selection for adjuvant therapy. Overall, 29% ccRCC patients met the KEYNOTE-564 criteria. When stratified by the Leibovich and UroPredict scores, these patients fell into heterogeneous prognostic groups. Median follow-up was 24 months (IQR 9.5– 48.5). The best cut-offs to maximize sensitivity and negative predictive value were 5 (Leibovich score) and 0.23 (UroPredict). DFS was significantly lower in patients with a Leibovich score > 5 (HR 3.4, 95% CI 1.2–9.5, p=0.025) as well as in those with UroPredict >0.23 (log-rank-p = 0.037) (Figure). Applying the Leibovich score (<5 vs >5) would spare adjuvant therapy in 36 (24%) patients, 32 (89%) of whom would avoid unnecessary treatment, whereas using the UroPredict (<0.23 vs >0.23) would spare adjuvant therapy in 19 (13%) patients, all of whom would avoid unnecessary treatment. The Leibovich 2003 and UroPredict scores identify a subset of patients at worse prognosis among those meeting the KN564 criteria, supporting individualized shared decision-making on adjuvant pembrolizumab.



# Abstract: The accuracy of KEYNOTE-564 study eligibility criteria on recurrence prediction in papillary renal cell carcinoma: A multi-institutional comparative analysis with prognostic scores

**AUTHORS**

Selcuk Erdem, Istanbul University Istanbul Faculty of Medicine, Division of Urologic Oncology, Department of Urology, Istanbul, Turkey

Riccardo Campi, University of Florence, Unit of Urological Robotic Surgery and Renal Transplantation, Department of Experimental and Clinical Medicine, Florence, Italy

Tobias Klatte, Helios Klinikum Bad Saarow, Department of Urology, Bad Saarow, Germany

Daniele Amparore, University of Turin School of Medicine, San Luigi Gonzaga Hospital, Division of Urology, Department of Oncology, Orbassano, Turin, Italy

Maria Carme Mir, Mount Sinai Icahn School of Medicine, Elmhurst/Queens Hospital, Department of Urology, New York, NY, USA

Umberto Capitanio, IRCCS San Raffaele Scientific Institute, Department of Urology and Division of Experimental Oncology, Urological Research Institute, Milan, Italy

Eduard Roussel, University Hospitals Leuven, Department of Urology, Leuven, Belgium

Riccardo Bertolo, Azienda Ospedaliera Universitaria Integrata of Verona, Department of Urology, Verona, Italy

Nicola Pavan, University of Palermo, Urology Clinic, Department of Precision Medicine in Medical Surgical and Critical Care, Palermo, Italy

Onder Kara⼗, Istanbul University Istanbul Faculty of Medicine, Division of Urologic Oncology, Department of Urology, Istanbul, Turkey

Zhenjie Wu, Changhai Hospital Naval Medical University, Department of Urology, Shanghai, China

Umberto Anceschi, IRCCS Regina Elena National Cancer Institute, Department of Urology, Rome, Italy

Umberto Carbonara, University of Bari, Unit of Andrology and Kidney Transplantation, Department of Emergency and Organ Transplantation-Urology, Bari, Italy

Yasin Ates, Istanbul University Istanbul Faculty of Medicine, Division of Urologic Oncology, Department of Urology, Istanbul, Turkey

Mert Erden, Istanbul University Istanbul Faculty of Medicine, Division of Urologic Oncology, Department of Urology, Istanbul, Turkey

Sergio Serni, University of Florence, Unit of Urological Robotic Surgery and Renal Transplantation, Department of Experimental and Clinical Medicine, Florence, Italy

Oner Sanli, Istanbul University Istanbul Faculty of Medicine, Division of Urologic Oncology, Department of Urology, Istanbul, Turkey

Faruk Ozcan, Istanbul University Istanbul Faculty of Medicine, Division of Urologic Oncology, Department of Urology, Istanbul, Turkey

Axel Bex, Royal Free London NHS Foundation Trust, University College London, Division of Surgery and Interventional Science, London, UK

Grant Stewart, University of Cambridge, Department of Surgery, Cambridge, UK

Introduction&Objectives: Although the KEYNOTE-564 study was conducted in clear cell renal cell carcinoma (ccRCC), its eligibility criteria might also stratify recurrence risk in papillary RCC (papRCC). This study aimed to evaluate the accuracy of the KEYNOTE-564 eligibility criteria for recurrence prediction in a large multi-institutional non-metastatic papRCC cohort. Materials&Methods: A total of 1826 non-metastatic papRCC patients undergoing curative surgery at twelve tertiary institutions were retrospectively documented and included in this study. Patients were categorized in 2 groups by KEYNOTE-564 criteria (eligible vs. ineligible), and also stratified by the risk groups of GRANT, VENUSS and Leibovich-2018 prognostic scores. The primary outcome was defined as recurrence-free survival (RFS). Results: Overall, 358 patients (19.6%) were eligible, and 1468 (80.4%) were ineligible. Eligible patients were significantly older (65 vs. 63 years, p=0.002), frequently male (81.0% vs. 75.7%, p=0.033), and symptomatic (32.6% vs. 15.6%, p<0.001). They had larger tumors (4.0 vs. 3.6 cm, p<0.001), higher grade (G3–4: 34.3% vs. 11.1%, p<0.001), and more necrosis (51.1% vs. 25.7%, p<0.001). After median follow-up of 39 months, 221 patients (12.1%) recurred. Estimated 5-year RFS (58.9% vs. 93.0%,p<0.001) was significantly lower in the eligible group. In multivariable Cox regression, male gender (p=0.018), GRANT (p<0.001) and VENUSS (p<0.001) classifications independently predicted RFS, whereas KEYNOTE-564 criteria did not. The concordance (C) indices for recurrence prediction were 72.3% for GRANT score, 69.9% for GRANT classification, 79.3% for VENUSS score, 77.3% for VENUSS classification, 73.6% for Leibovich-2018 classification, and 71.4% for KEYNOTE-564 criteria. Conclusions: KEYNOTE-564 eligibility criteria can predict RFS in non-metastatic papRCC after curative surgery. However, its predictive accuracy is inferior to established prognostic models such as GRANT, VENUSS, and Leibovich-2018. Among these, the VENUSS score demonstrated the highest prognostic accuracy for postoperative recurrence risk in non-metastatic papRCC.



# Abstract: Defining subtype-specific PD-1, PD-L1 and CTLA-4 immunopositivity thresholds for personalized prognostication in surgically treated non-metastatic renal cell carcinoma

**AUTHORS**

Selcuk Erdem, Istanbul University Istanbul Faculty of Medicine, Division of Urologic Oncology, Department of Urology, Istanbul, Turkey

Serdar Turan, Istanbul University Istanbul Faculty of Medicine, Division of Urologic Oncology, Department of Urology, Istanbul, Turkey

Yasin Ates, Istanbul University Istanbul Faculty of Medicine, Division of Urologic Oncology, Department of Urology, Istanbul, Turkey

Tunc Aydinoglu, Istanbul University Istanbul Faculty of Medicine, Division of Urologic Oncology, Department of Urology, Istanbul, Turkey

Ozge Hurdogan, Istanbul University Istanbul Faculty of Medicine, Department of Pathology, Istanbul, Turkey

Yasemin Ozluk, Istanbul University Istanbul Faculty of Medicine, Department of Pathology, Istanbul, Turkey

Oner Sanli, Istanbul University Istanbul Faculty of Medicine, Division of Urologic Oncology, Department of Urology, Istanbul, Turkey

Faruk Ozcan, Istanbul University Istanbul Faculty of Medicine, Division of Urologic Oncology, Department of Urology, Istanbul, Turkey

Introduction&Objectives: The randomized controlled trials on adjuvant immunotherapy in renal cell carcinoma (RCC) have been conducted in clear cell RCC (ccRCC), and programmed death-ligand 1 (PD-L1) with 1% immunopositivity threshold was the only biomarker utilized. This study aimed to compare the prognostic value of different immunopositivity thresholds (1%, 2%, 5%, and 10%) for programmed death-1 (PD-1), PD-L1, and cytotoxic T-lymphocyte–associated protein 4 (CTLA-4) among ccRCC, papillary RCC (papRCC), and chromophobe RCC (chRCC). Materials&Methods: A total of 103 patients undergoing curative surgery for non-metastatic RCC were included: 34 ccRCC, 30 papRCC, and 39 chRCC. Formalin-fixed paraffin-embedded tissues were assessed by immunohistochemistry for PD-1, PD-L1, and CTLA-4 expression. Tumor proportion score (TPS), combined positivity score (CPS) and immune cell (IC) score were used for PD-L1, and IC score for PD-1 and CTLA-4. Patients were categorized into “low” and “high” groups according to 1%, 2%, 5%, and 10% immunopositivity thresholds. Recurrence-free survival (RFS) was compared between groups in overall cohort and within each RCC subtype. Results: In overall cohort, only 1% threshold for PD-1 significantly predicted 2-year RFS (p=0.014), whereas other thresholds did not. In papRCC subgroup, PD-1 expression significantly predicted 2-year RFS at all thresholds—1% (p=0.008), 2% (p=0.012), 5% (p<0.001), and 10% (p=0.002). Furthermore, 10% threshold was also predictive for PD-L1 TPS (p=0.007), PD-L1 CPS (p=0.030), and CTLA-4 (p<0.001) in papRCC. None of the markers were associated with RFS in ccRCC or chRCC at any threshold. Conclusions: This study demonstrates that all immunopositivity thresholds for PD-1 and 10% thresholds for PD-L1 TPS, PD-L1 CPS, and CTLA-4 provided significant predictive value on RFS, whereas no association was observed in ccRCC or chRCC. These findings may guide future biomarker-driven prognostication and adjuvant trial design in papRCC.

# Abstract: Circulating KIM-1 and microRNA Signatures Predict Response and Survival in Advanced Renal Cell Carcinoma Treated With Neoadjuvant Therapy

**AUTHORS**

David Pelegrina Sánchez, University Hospital 12 de Octubre, Madrid, Spain

Jorge Esteban Villarubia, University Hospital 12 de Octubre, Madrid, Spain

Esther Montesinos, Instituto de Investigación Imas12, Madrid, Spain

Ignacio A. Reina, Instituto de Investigación Imas12, Madrid, Spain

Álvaro Martín de Bernardo, Instituto de Investigación Imas12, Madrid, Spain

Laura García-Gómez, Instituto de Investigación Imas12, Madrid, Spain

Carolina Rubio, Instituto de Investigación Imas12, Madrid, Spain

Jesús M. Paramio, Instituto de Investigación Imas12, Madrid, Spain

Marta Dueñas, Instituto de Investigación Imas12, Madrid, Spain

Guillermo de Velasco, University Hospital 12 de Octubre, Madrid, Spain

INTRODUCTION: Advanced renal cell carcinoma (mRCC) remains a clinically heterogeneous disease, in which optimal selection of patients for cytoreductive nephrectomy (CN) in the era of targeted therapies is still debated. Reliable biomarkers to predict response to neoadjuvant therapy and survival outcomes are needed to guide treatment sequencing and surgical decision-making.

OBJECTIVES: This study evaluates the clinical and prognostic value of circulating biomarkers in patients with mRCC enrolled in the prospective CABOPRE trial (NCT06377722). Patients with mRCC received 12 weeks of neoadjuvant cabozantinib followed by CN and continuation of treatment. This analysis focuses on circulating kidney injury molecule-1 (KIM-1) and plasma/exosomal microRNAs as predictors of treatment response, tumour burden dynamics, and survival outcomes. METHODS: Plasma samples were collected prior to initiating neoadjuvant cabozantinib and at the time of cytoreductive nephrectomy. Circulating and exosomal miRNA expression profiles were analyzed using a multiplex expression assay. Circulating KIM-1 levels were quantified by ELISA and correlated with treatment response and progression-free survival (Graph I).

RESULTS: KIM-1 levels were significantly higher in patients with early progression and inferior survival outcomes at the time of surgery. A similar trend was observed in baseline samples, supporting its value as a negative prognostic biomarker (Graph I). Distinct circulating miRNA expression patterns were observed based on treatment response and survival outcomes. Responders showed upregulation of hsa-miR-374a-5p, hsa-let-7 family members, and hsa-miR-126-3p, whereas non-responders demonstrated increased expression of hsa-miR-1224-3p, hsa-miR-429, and hsamiR-548. Longer survival (≥12 months) was associated with higher expression of hsa-miR-150-5p and hsa-miR-31-5p.

CONCLUSIONS: Circulating biomarkers derived from liquid biopsy show potential clinical utility in mRCC treated with neoadjuvant therapy. Elevated KIM-1 identifies patients with unfavourable outcomes, while specific circulating miRNA signatures are associated with response and survival, supporting future biomarker-driven patient selection strategies.



# Abstract: Association of long non-coding RNAs expression and transcriptomic patterns with treatment outcomes in metastatic renal cell carcinoma

**AUTHORS**

Kamila Koucká, Laboratory of Pharmacogenomics, Biomedical Center, Faculty of Medicine in Pilsen, Charles University, Pilsen, Czech Republic; Toxicogenomics Unit, National Institute of Public Health, Prague, Czech Republic

Tereza Tesařová, Laboratory of Pharmacogenomics, Biomedical Center, Faculty of Medicine in Pilsen, Charles University, Pilsen, Czech Republic; Toxicogenomics Unit, National Institute of Public Health, Prague, Czech Republic

Radka Václavíková, Laboratory of Pharmacogenomics, Biomedical Center, Faculty of Medicine in Pilsen, Charles University, Pilsen, Czech Republic; Toxicogenomics Unit, National Institute of Public Health, Prague, Czech Republic

Karolína Šeborová, Laboratory of Pharmacogenomics, Biomedical Center, Faculty of Medicine in Pilsen, Charles University, Pilsen, Czech Republic; Toxicogenomics Unit, National Institute of Public Health, Prague, Czech Republic

Milan Hora, Department of Urology, Faculty of Medicine in Pilsen and University Hospital, Charles University, Pilsen, Czech Republic

Ondřej Hes, Department of Pathology, Faculty of Medicine in Pilsen and University Hospital, Charles University, Pilsen, Czech Republic

Kristýna Pivovarčíková, Department of Pathology, Faculty of Medicine in Pilsen and University Hospital, Charles University, Pilsen, Czech Republic

Pavel Souček, Laboratory of Pharmacogenomics, Biomedical Center, Faculty of Medicine in Pilsen, Charles University, Pilsen, Czech Republic; Toxicogenomics Unit, National Institute of Public Health, Prague, Czech Republic

Ondřej Fiala, Laboratory of Cancer Treatment and Tissue Regeneration, Biomedical Center, Faculty of Medicine in Pilsen, Charles University, Pilsen, Czech Republic; ARON Research Foundation ETS, Macerata, Italy

Long non-coding RNAs (lncRNAs) are key regulators of cancer development and are increasingly recognized as molecular biomarkers. This study examined the association between cancer-related lncRNAs, response to the tyrosine kinase inhibitor sunitinib, transcriptomic alterations, and clinical outcomes in patients with metastatic renal cell carcinoma (mRCC). Paired tumor and adjacent non-malignant renal tissue samples were obtained from 38 patients with metastatic clear cell RCC treated with first-line sunitinib. Expression of 84 cancer-associated lncRNAs was analyzed using qPCR. In a subgroup of 20 patients, RNA sequencing was performed to assess protein-coding gene expression and investigate lncRNA–mRNA associations. Differential expression analyses were conducted between tumor and non-malignant tissues and between patients with good and poor responses to sunitinib. Fifty lncRNAs were differentially expressed in tumor samples compared with non-malignant tissues. Stratification by treatment response identified 14 lncRNAs with significant expression differences. Higher expression of HNF1A-AS1 and IPW was associated with prolonged progression-free survival, elevated TUSC7 expression correlated with poor response to sunitinib. In total, levels of 26 lncRNAs were significantly correlated with mRNA expression levels of 65 protein-coding genes. The lncRNAs with the highest number of correlations with protein-coding genes were MEG3 and SNHG16. And higher expression of CLIP4 was observed in patients responding well to sunitinib. Overall, this study identifies lncRNAs with predictive and prognostic relevance in mRCC treated with sunitinib, supporting the potential of lncRNA-based profiling in personalized treatment strategies. Supported by the Ministry of Health of the Czech Republic in cooperation with the Czech Health Research Council under project No. NW25-03-00122, by the Charles University Research Fund (Cooperatio program No. 207043 – Surgical disciplines), and by the project „Integration of biomedical research and health care in the Pilsen metropolitan area“; reg. no. CZ.02.01.01/00/23_021/0008828) - co-funded by the European Union and by the State Budget of the Czech Republic.

# Abstract: First interim analysis of the SIGNS-RCC multicenter study for the identification of prognostic and predictive factors in renal cell carcinoma

**AUTHORS**

Matteo Rosellini, Department of Medical and Surgical Sciences (DIMEC), University of Bologna, Bologna, Italy

Veronica Mollica, IRCCS Azienda Ospedaliero-Universitaria di Bologna, Bologna, Italy

Ilaria Zampiva, IOV Istituto Oncologico Veneto IRCCS UOC Oncologia 1, Padua, Italy

Stefano Manduca, University of Verona, Department of Engineering for Innovation Medicine, Section of Innovation Biomedicine, Oncology Area, Verona, Italy

Emanuela Fantinel, Azienda Ospedaliera Universitaria Integrata di Verona, Medical Oncology Unit, Verona, Italy

Sara Elena Rebuzzi, Medical Oncology Unit 2, Azienda Ospedaliero-Universitaria Città della Salute e Della Scienza di Torino, Ospedale Molinette, Turin, Italy

Davide Bimbatti, IOV Istituto Oncologico Veneto IRCCS UOC Oncologia 1, Padua, Italy

Guido Martignoni, University of Verona, Department of Diagnostics and Public Health, Section of Pathology, Verona, Italy

Jessica Menis, Azienda Ospedaliera Universitaria Integrata di Verona, Medical Oncology Unit, Verona, Italy

Cecilia Nasso, Ospedale Santa Corona, Department of Oncology, Pietra Ligure, Italy

Michela Piacentini, University of Verona, Department of Engineering for Innovation Medicine, Section of Innovation Biomedicine, Oncology Area, Verona, Italy

Francesco Massari, Department of Medical and Surgical Sciences (DIMEC), University of Bologna, Bologna, Italy

Sebastiano Buti, Medicine and Surgery Department, University of Parma, Parma, Italy

Roberto Iacovelli, Medical Oncology, Università Cattolica del Sacro Cuore, Fondazione Policlinico Universitario Agostino Gemelli IRCCS, Rome, Italy

Ester Tasselli, Centro Ricerche Cliniche, Azienda Ospedaliera Integrata, Verona, Italy

Anna Sordo, Centro Ricerche Cliniche, Azienda Ospedaliera Integrata, Verona, Italy

Francesco Pierantoni, IOV Istituto Oncologico Veneto IRCCS, Department of Oncology - UOC Oncologia 3, Castelfranco Veneto, Italy

Giuseppe Verlato, University of Verona, Department of Diagnostics and Public Health, Section of Epidemiology and Medical Statistics, Verona, Italy

Michele Milella, University of Verona, Department of Engineering for Innovation Medicine, Section of Innovation Biomedicine, Oncology Area, Verona, Italy

Anna Caliò, University of Verona, Department of Diagnostics and Public Health, Section of Pathology, Verona, Italy

Background: SIGNS-RCC is a multicohort, multicentric study aimed at identifying prognostic and predictive biomarkers for renal cell carcinoma (RCC). 3 cohorts are included: a retrospective cohort (A) including metastatic RCC patients treated with first-line TKI; a prospective cohort (B) enrolling first-line IO+TKI treated patients; a retrospective cohort (C) of radically resected patients. We present the results of the first-interim analysis of cohort A/B. Methods: Aims of the study (Fig.1) are: 1. Identification of a new clinical prognostic score using patient-/tumor-related variables. 2. Identification of tissue-based signatures. Tumor tissue will be analyzed by IHC, FISH, NGS, multiplex IHC, and RNAscope to define RCC signatures and TME sources. 3. Identification of circulating signatures (cohort B). Circulating cytokines will be quantified by ELISA, with exploratory PBMC subset analysis (MDSCs, CD4^+^CD25high Tregs) in selected patients. Results: Patient enrollment started in August 2023 in 10 Italian Centers. To date, 91 patients have been enrolled in Cohort A and 90 in Cohort B, exceeded 50% of the planned sample size. In cohort A, median PFS was 34.7 months. Significant prognostic factors for PFS at multivariate analysis were sarcomatoid dedifferentiation (HR 2.98, p=0.013), N1 (HR 4.81, p<0.001), nephrectomy (HR 0.2, p<0.001). In cohort B, pembrolizumab/lenvatinib was the most common treatment (70.5%), followed by pembrolizumab/axitinib (15.4%) and nivolumab/cabozantinib (14.1%). After a median follow-up of 5.8 months, median PFS in the overall population was not reached (95%CI 1.9-NA). Regarding ORR, sarcomatoid differentiation, peritoneal and CNS metastases was significantly associated with lower odds of response (p=0.046, p=0.005, p=0.047, respectively), while no associations were found for histology, grading, prior nephrectomy, IMDC groups, first-line regimens, and circulating biomarkers at baseline. Conclusions: Preliminary analyses confirm project feasibility with solid progress; final results require completed enrollment and longer follow-up.



# Abstract: Microbial Dysbiosis as a Determinant of Clinical Outcomes with CBM588 in Metastatic Renal Cell Carcinoma (mRCC)

**AUTHORS**

Ruchi Agarwal, City of Hope Comprehensive Cancer Center, Duarte, CA, USA

Nazli Dizman, MD Anderson Cancer Center, Houston, TX, USA

Ali Moradi, Beth Israel Deaconess Medical Center, Boston, MA, USA

Hedyeh Ebrahimi, City of Hope Comprehensive Cancer Center, Duarte, CA, USA

Xinran Qi, City of Hope Comprehensive Cancer Center, Duarte, CA, USA

Antonio Liuz Cruz Gomes, City of Hope Comprehensive Cancer Center, Duarte, CA, USA

Miguel Zugman, City of Hope Comprehensive Cancer Center, Duarte, CA, USA

Vitor Abreu de Goes, City of Hope Comprehensive Cancer Center, Duarte, CA, USA

Salvador Jaime-Casas, City of Hope Comprehensive Cancer Center, Duarte, CA, USA

Xiaochen Li, City of Hope Comprehensive Cancer Center, Duarte, CA, USA

Tanya B. Dorff, City of Hope Comprehensive Cancer Center, Duarte, CA, USA

Joann Hsu, City of Hope Comprehensive Cancer Center, Duarte, CA, USA

Alexander Chehrazi-Raffle, City of Hope Comprehensive Cancer Center, Duarte, CA, USA

Motomichi Takahashi, Miyarisan Pharmaceutical Co., Ltd., Tokyo, Japan

Kentaro Oka, Miyarisan Pharmaceutical Co., Ltd., Tokyo, Japan

Seiya Higashi, Miyarisan Pharmaceutical Co., Ltd., Tokyo, Japan

Peter P. Lee, Osel, Inc., Mountain View, CA, USA

Thomas P. Parks, Osel, Inc., Mountain View, CA, USA

Jennifer Karmouch, City of Hope Comprehensive Cancer Center, Duarte, CA, USA

Robert Jenq, City of Hope Comprehensive Cancer Center, Duarte, CA, USA

Sumanta Pal, City of Hope Comprehensive Cancer Center, Duarte, CA, USA

Introduction: Two early-phase trials showed that CBM588, a Clostridium butyricum-based live bacterial product, modulates the gut microbiome to improve response to immune-checkpoint inhibitor (ICI)-based standard of care (SOC) combinations (nivolumab/ipilimumab and nivolumab/cabozantinib) in mRCC. Beyond exploring the mechanism of action and therapeutic potential of CBM588, these studies provide a novel setting to explore the microbial biomarkers to guide treatment selection. Herein, we examine microbial dysbiosis as a determinant of outcomes with CBM588. Methods: We conducted a pooled analysis of two trials in which patients were randomized (2:1) to SOC with CBM588 or SOC alone. Microbial dysbiosis was assessed via the previously published TOPOSCORE assessment and computed as a continuous variable (S score) with higher scores indicating worse microbial dysbiosis. Baseline S scores were compared between patients with and without response (R and NR assessed by RECIST 1.1) and by ICI-backbone using the Mann-Whitney U test. Results: Among 59 patients included, 39 received SOC+CBM588 and 20 received SOC. At a median follow-up of 44.2 months, the objective response rate was 69.2% vs 20.0% with SOC+CBM588 versus SOC (p=0.001). In the overall cohort, the median baseline S score was 0.58 (IQR 0.49-0.67) and 0.70 (IQR 0.56-0.83) in R and NR with SOC+CBM588 (p=0.155), and 0.74 (IQR 0.60-0.87) and 0.81 (IQR 0.79-0.86) in R and NR with SOC alone (p=0.360). In patients who received nivolumab/ipilimumab as the SOC backbone, median S score was 0.67 (IQR 0.62-0.81) in R versus 0.54 (IQR 0.43-0.61) in NR with CBM588 (p=0.044), while no association was observed in those who received nivolumab/cabozantinib backbone with CBM588 (median S score 0.71 [IQR 0.52-0.85] in R vs 0.82 [IQR 0.69-0.83], p=0.738). Conclusion: Our findings reinforce the growing body of evidence linking dysbiosis and outcomes with ICI-based treatments in mRCC and offer insights into its backbone-dependent behavior as a biomarker.

# Abstract: Integrated prognostic signature to improve risk group stratification in metastatic renal cell carcinoma

**AUTHORS**

Stefano Manduca, University of Verona, Department of Engineering for Innovation Medicine, Section of Innovation Biomedicine, Oncology Area, Verona, Italy

Matteo Rosellini, University of Bologna, Bologna, Italy

Veronica Mollica, IRCCS Azienda Ospedaliero-Universitaria di Bologna, Bologna, Italy

Ilaria Zampiva, IOV Istituto Oncologico Veneto IRCCS UOC Oncologia 1, Padua, Italy

Emanuela Fantinel, Azienda Ospedaliera Universitaria Integrata di Verona, Medical Oncology Unit, Verona, Italy

Sara Elena Rebuzzi, Medical Oncology Unit 2, Ospedale Molinette, Azienda Ospedaliero-Universitaria Città della Salute e Della Scienza di Torino, Turin, Italy

Davide Bimbatti, IOV Istituto Oncologico Veneto IRCCS UOC Oncologia 1, Padua, Italy

Matteo Brunelli, University of Verona, Department of Diagnostics and Public Health, Section of Pathology, Verona, Italy

Guido Martignoni, University of Verona, Department of Diagnostics and Public Health, Section of Pathology, Verona, Italy

Jessica Menis, Azienda Ospedaliera Universitaria Integrata di Verona, Medical Oncology Unit, Verona, Italy

Cecilia Nasso, Ospedale Santa Corona, Department of Oncology, Pietra Ligure, Italy

Michela Piacentini, University of Verona, Department of Engineering for Innovation Medicine, Section of Innovation Biomedicine, Oncology Area, Verona, Italy

Pierpaolo Marchetti, University of Verona, Department of Diagnostics and Public Health, Section of Epidemiology and Medical Statistics, Verona, Italy

Roberta Vesentini, University of Verona, Department of Diagnostics and Public Health, Section of Epidemiology and Medical Statistics, Verona, Italy

Giuseppe Verlato, University of Verona, Department of Diagnostics and Public Health, Section of Epidemiology and Medical Statistics, Verona, Italy

Francesco Pierantoni, IOV Istituto Oncologico Veneto IRCCS, Department of Oncology - UOC Oncologia 3, Castelfranco Veneto, Italy

Francesco Massari, Department of Medical and Surgical Sciences (DIMEC), University of Bologna, Bologna, Italy

Marco Maruzzo, Oncology 3 Unit, Istituto Oncologico Veneto IRCCS, Padua, Italy

Michele Milella, University of Verona, Department of Engineering for Innovation Medicine, Section of Innovation Biomedicine, Oncology Area, Verona, Italy

Anna Caliò, University of Verona, Department of Engineering for Innovation Medicine, Section of Innovation Biomedicine, Oncology Area, Verona, Italy

Background: Prognostic factors for metastatic RCC (mRCC) are warranted to guide treatment management. Herein, we present the results of a retrospective cohort of mRCC patients who received first-line TKI monotherapy at four Italian Institutions between 2013 and 2021. Methods: The purpose was to assess the feasibility of developing an integrated prognostic signature incorporating both clinical and tissue-based features. The primary endpoint was OS. A three-group model was constructed based on survival distributions, stratifying patients into favorable, intermediate, and poor-risk subgroups. Agreement between the new score and the IMDC score was assessed, and their respective prognostic performances were compared by calculating the AUC from ROC analysis. We performed exploratory clinical and tissue-based analyses focused on the “outliers” (if their survival outcome exceeded or defected at least 50% from what predicted by IMDC risk score). Tissue blocks were obtained by either nephrectomy or biopsy. IHC was performed for tumor microenvironment characterization (CD8, CD15, CD56), BAP1 and PBRM1 mutational status. FISH was used to assess chromosome 9p loss. Results: 193 patients were enrolled, 75 (38.9%) were classified as IMDC good, 77 (39.9%) as intermediate, and 41 (21.2%) as poor risk. Median OS for the entire population was 47.8 months. Median OS was 65.8 months for the good-risk, 44.0 months for the intermediate-risk, and 12.9 months for the poor-risk group. At univariate analysis, statistically significant prognostic factors for OS were: high NLR, bone metastases, pancreatic metastases, CNS metastases, active surveillance, all IMDC factors with the exception of thrombocytosis. At multivariate analysis, anemia, neutrophilia and Karnofsky PS confirmed their prognostic role; LDH > 1.5 ULN, pancreatic and CNS metastases demonstrated additional significant performance and entered the model. The resulting six-factors model categorized patients into three risk subgroups: 0-1 factors categorized as favorable risk (82 patients, 56.2%, mOS 65.5 months), 2-3 as intermediate risk (54 patients, 37%, mOS 24.9 months), ≥4 as poor risk (10 patients, 6.8%, mOS 4.6 months). 33 intermediate-risk patients (52.4%) were reclassified as favorable-risk, while 4 favorable-risk (7.4%) and 21 poor-risk patients (72.4%) were reclassified as intermediate-risk according to the new score. The new score showed slightly improved performance (AUC 0.63 vs 0.61) and demonstrated 79.1% concordance with the IMDC score (p = 0.178). Analysis of the overall population identified a substantial proportion of “outliers” (40.9%). Preliminary results indicated that PBRM1 and BAP1 mutations were significantly associated with better-than-expected survival (p = 0.041 and 0.047). Conclusions: A new six-factors prognostic score is able stratify survival of mRCC patients with a similar performance to IMDC score.



# Abstract: SDH-Deficient Renal Cell Carcinoma: A Case Series of Four Patients

**AUTHORS**

Nektarios Alevizopoulos, Evaggelismos General Hospital, Athens, Greece

Michael G. Pavlakis, Metropolitan General Hospital, Athens, Greece

Georgios Kanellopoulos, Evaggelismos General Hospital, Athens, Greece

Ioannis Xoxakos, Evaggelismos General Hospital, Athens, Greece

Christina Vourlakou, Evaggelismos General Hospital, Athens, Greece

Vaios Oreopoulos, Evaggelismos General Hospital, Athens, Greece

Background: Succinate dehydrogenase (SDH)-deficient renal cell carcinoma (RCC) is a rare subtype of RCC, often associated with germline mutations and hereditary tumor syndromes. Early recognition is essential for appropriate management and genetic counseling.First described in 2004, this subtype often affects young adults and may occur as part of hereditary paraganglioma-pheochromocytoma syndromes. SDH-deficient RCC is distinguished by eosinophilic, vacuolated cytoplasm and loss of SDHB immunostaining. Methods: We present four patients diagnosed with SDH-deficient RCC at our institution, detailing clinical presentation, histopathology, immunohistochemistry, genetic findings, management, and outcomes. Results: Tumor size ranged from 3.5 to 5 cm. Histologically, tumors exhibited eosinophilic, vacuolated cytoplasm. SDHB immunohistochemistry was negative in all cases. Germline SDHB mutations were detected in two patients. Partial nephrectomy was performed in three cases and radical nephrectomy in one. No local recurrences were observed during a median follow-up of 24 months. Conclusion: SDH-deficient RCC is rare but clinically significant entity. Diagnosis via immunohistochemistry and genetic testing informs patient management and family counseling. Awareness of this entity prevents misclassification and guides treatment.SDH-deficient RCC,should be considered in young patients with eosinophilic renal tumors. Immunohistochemistry for SDHB and genetic testing are essential for accurate diagnosis, management, and familial risk assessment.

# Abstract: Renal Metastases from Non Renal Primary Cancers: A Case Series of Five Patients and Review of the Literature

**AUTHORS**

Nektarios Alevizopoulos, Evaggelismos General Hospital, Athens, Greece

Michael G. Pavlakis, Iaso General Hospital, Athens, Greece

Georgios Kanellopoulos, Evaggelismos General Hospital, Athens, Greece

Vaios Oreopoulos, Evaggelismos General Hospital, Athens, Greece

Background: Metastases to the kidney from non renal primary cancers are clinically uncommon despite their relative frequency in autopsy series.Most published literature comprises case reports and small series; larger clinical datasets remain limited. Methods: We retrospectively reviewed 5 patients diagnosed with renal metastases at Evaggelismos General Hospital from January 2015 to December 2025. Demographics, clinical presentation, imaging, histopathology, management, and outcomes were analyzed. Results: The cohort included 5 patients (mean age 72, M:F ratio 4:1). Primary tumors included lung carcinoma (n=2), colorectal adenocarcinoma (n=2), and ovarian cancer (n=1).Renal involvement was simultaneous with systemic disease in most cases. Imaging typically showed hypodense renal lesions on CT/MRI scans. Histopathological confirmation was obtained by core biopsy (n=4) or nephrectomy( n=1). Management varied from systemic therapy to nephrectomy.Biopsy helps avoid unnecessary nephrectomy unless solitary symptomatic lesion necessitates resection.Prognosis remains variable; dependent on primary cancer and extent of systemic disease. Conclusions: Renal metastases are rare clinical findings. In patients with known primary malignancies, incidental renal masses should prompt consideration of metastatic disease; confirmatory biopsy can avoid unnecessary nephrectomy. Surgical intervention may be indicated in solitary metastasis with symptomatology.Renal metastasis from extrarenal tumors, though rare, should be recognized in differential diagnosis of renal masses in oncologic patients. A multidisciplinary approach enhances prompt diagnosis and tailored management.

# Abstract: Cabozantinib in Chromophobe Renal Cell Carcinoma: A Case Series of Five Patients Assessing Progression-Free Survival and Quality of Life

**AUTHORS**

Nektarios Alevizopoulos, Evaggelismos General Hospital, Oncology Department, Athens, Greece

Michael G. Pavlakis, Metropolitan General Hospital, Haemato-Oncology Department, Athens, Greece

Panagiotis Bacarakos, Metropolitan General Hospital, Haemato-Oncology Department, Athens, Greece

Marina Tsantekidou, Evaggelismos General Hospital, Oncology Department, Athens, Greece

Georgios Kanellopoulos, Metropolitan General Hospital, Haemato-Oncology Department, Athens, Greece

Maria Vasilaki-Antonatou, Evaggelismos General Hospital, Oncology Department, Athens, Greece

Christina Vourlakou, Evaggelismos General Hospital, Oncology Department, Athens, Greece

Background: Chromophobe renal cell carcinoma (chRCC) is an uncommon subtype of renal neoplasm, and therapeutic strategies in the metastatic context remain limited.Cabozantinib, a multi-targeted tyrosine kinase inhibitor, has demonstrated efficacy in clear cell RCC; however, data pertaining to chRCC is scarce yet increasingly encouraging in this challenging therapeutic landscape for renal malignancies. Methods: Five patients diagnosed with metastatic chromophobel renal cell carcinoma (chRCC) who were given cabozantinib at a single oncological institution were subjected to a retrospective analysis. The outcomes evaluated encompassed progression-free survival (PFS), radiographic response, adverse events classified according to CTCAE v5.0, and functional status assessed through ECOG performance status. Results: Median age at treatment initiation was 68 years (range 56–80). All patients had previously undergone nephrectomy and had received one to two systemic therapeutic trials; however, they unfortunately experienced substantial progression characterized by the emergence of new metastatic lesions. The median progression-free survival (PFS) was 7.8 months. Optimal responses included partial remissions in three patients and stable disease in two patients. The majority of adverse events were classified as grade 1–2, encompassing diarrhea, fatigue, and hypertension; no grade 4–5 events were documented. The ECOG performance status was either maintained or exhibited minimal improvement in four patients, reflecting a preserved functional quality of life. Conclusions: Cabozantinib exhibited notable disease control with an acceptable safety profile and maintained functional status in this limited cohort of metastatic chromophobe renal cell carcinoma (chRCC) patients. These findings advocate for its consideration in this uncommon subtype of renal cell carcinoma and emphasize the critical need for further studies.

# Abstract: Quality of Life in Elderly Patients (≥75 Years) With Advanced Renal Cell Carcinoma ARCC Treated With Avelumab–Axitinib and Nivolumab–Cabozantinib: A Case Series

**AUTHORS**

Nektarios Alevizopoulos, Evaggelismos General Hospital, Athens, Greece

Georgios Kanellopoulos, Evaggelismos General Hospital, Athens, Greece

Vaios Oreopoulos, Evaggelismos General Hospital, Athens, Greece

Michael G. Pavlakis, Evaggelismos General Hospital, Athens, Greece

Background: Combination regimens incorporating immune checkpoint inhibitors (ICIs) and tyrosine kinase inhibitors (TKIs), such as avelumab–axitinib and nivolumab–cabozantinib, are established treatment options for advanced renal cell carcinoma (aRCC). However, elderly patients are frequently underrepresented in clinical trials, and real-world data addressing quality of life (QoL), frailty, and functional status in this population remain limited. Methods: This retrospective case series included fifteen elderly patients (aged ≥75 years) with aRCC treated with either avelumab–axitinib (n = 8) or nivolumab–cabozantinib (n = 7). Baseline geriatric assessment comprised Eastern Cooperative Oncology Group (ECOG) performance status, comorbidity burden, and clinical frailty evaluation. Quality of life was assessed using the European Organisation for Research and Treatment of Cancer Quality of Life Questionnaire (EORTC QLQ-C30) at baseline and at 3 and 6 months following treatment initiation. Treatment-related adverse events were recorded and graded according to the Common Terminology Criteria for Adverse Events (CTCAE), version 5.0. Results: The median age of the cohort was 78 years (range 75–85). Most patients had an ECOG performance status of 1 and a moderate comorbidity burden, while mild to moderate frailty was observed in a subset at baseline. In the avelumab–axitinib group, mean global health status/QoL scores showed a modest improvement from baseline to 3 months (70 to 74) and remained stable at 6 months (72). Patients receiving nivolumab–cabozantinib demonstrated overall stable QoL scores throughout follow-up (baseline 68; 3 months 69; 6 months 67). Fatigue and gastrointestinal toxicities were the most frequently reported adverse events. No grade 4 toxicities were observed, and no treatment discontinuations due to adverse events occurred. Conclusions: In this real-world cohort of elderly patients with aRCC, including individuals with baseline frailty and comorbidities, combination therapy with avelumab–axitinib or nivolumab– cabozantinib was associated with preservation of quality of life and an acceptable safety profile. These findings support the feasibility of contemporary combination regimens in carefully selected elderly patients and underscore the importance of integrating geriatric assessment and patient-reported outcomes into routine clinical practice. Larger prospective studies focusing on elderly and frail populations are warranted.

# Abstract: Effectiveness of Second-Line Treatments after Primary Resistance to Immune Checkpoint Inhibitor-Based Combinations in metastatic clear cell Renal Cell Carcinoma (mRCC)

**AUTHORS**

Adrien Marques Da Silva, Hôpital Européen Georges-Pompidou, Paris, France

Laurence Albigès, Gustave Roussy, Villejuif, France

Javier Gavira-Diaz, Gustave Roussy, Villejuif, France

Benjamin Auberger, CHU Brest, Brest, France

Pierre Bigot, CHU Angers, Angers, France

Clément Dumont, Hôpital Saint-Louis, Paris, France

Denis Maillet, Centre Hospitalier Lyon Sud, Lyon, France

Aurore Douge, CHU Clermont-Ferrand, Clermont-Ferrand, France

Loic Jaffrelot, Hôpital Pitié-Salpêtrière, Paris, France

Laetitia Augusto-Pelegrin, Centre Henri Becquerel, Rouen, France

Elouen Boughalem, Institut de Cancérologie de l'Ouest (ICO), Angers, France

Frédéric Rolland, Centre René Gauducheau, Saint-Herblain, France

Rafaele Ratta, Hôpital Foch, Suresnes, France

Pierre Combe, Pôle Santé Léonard de Vinci, Chambray-Lès-Tours, France

Tiffany Darbas, CHU Limoges, Limoges, France

Carole Helissey, Hôpital Begin, Vincennes, France

Melodie Carbonnaux, Centre Hospitalier d'Annecy, Annecy, France

Stéphane Oudard, Hôpital Européen Georges-Pompidou, Paris, France

Edouard Auclin, Hôpital Européen Georges-Pompidou, Paris, France

Introduction: Immune checkpoint inhibitor (ICI) combinations (combo) is the standard first-line (L1) treatment for mRCC. Primary resistance upon initiation of L1 treatment, occurs in approximately 10 to 20% of patients. No recommendations exist for second-line (L2) after primary resistance to L1 ICI combo. We aimed to describe the real world clinical practice, efficacy and safety of L2 treatment after primary progression under L1 ICI combo in mRCC. Methods: Retrospective, multicenter cohort study in France involving patients with mRCC with primary resistance L1 ICI combo and received L2 treatment. Primary resistance is defined as progression and change of treatment at the 3-month assessment. Primary endpoint : L2 objective response rate (ORR). Secondary endpoints :L2 PFS and OS, and tolerability. Results: From January 1, 2015, to June 30, 2024, 126 mRCC patients were included: 100 pts (79.4%) received L1 treatment with nivolumab-ipilimumab (NI), and 26 (20.6%) with ICI-TKI combos. L2 treatments were: cabozantinib (65.8%), axitinib (13.5%), sunitinib (7.9%), and pazopanib (3.2%). Additionally, 8 patients (6.3%) received a combo of ICI and TKI as L2 treatment. The median OS from L1 was 18,0 months. In L2 treatment, the ORR was 44%, and the DCR was 78%. The median PFS and OS were 7.9 and 13.8 months in the overall population with a median FU from L2 of 27.4 months. L2 ICI-TKI, pazopanib/sunitinib, or cabozantinib had a numerically higher ORR with 75%, 46% and 44%, respectively. Up to 116 patients (92.1%) in the study experienced at least one toxicity, and 54 (42.9%) a grade ≥ 3 toxicity. Results by type of L1 treatment are summarized in Table. Conclusion: We confirmed the poor prognosis of mRCC patients with primary resistance to ICI combo in L1. We found that L2 treatment were effective with absence of new toxicity signal. Pazopanib/sunitinib, cabozantinib or L2 ICI-TKI achieved interesting outcomes in this population.



# Abstract: Outcomes of immune-checkpoint inhibitor rechallenge in metastatic clear-cell renal cell carcinoma: results from a global real-world evidence study

**AUTHORS**

Antonio Cigliola, Department of Medical Oncology, IRCCS San Raffaele Hospital, Milan, Italy

Brigida Anna Maiorano, Department of Medical Oncology, IRCCS San Raffaele Hospital, Milan, Italy

Federico Rossari, Department of Medical Oncology, IRCCS San Raffaele Hospital, Milan, Italy

Valentina Tateo, Department of Medical Oncology, IRCCS San Raffaele Hospital, Milan, Italy

Chiara Mercinelli, Department of Medical Oncology, IRCCS San Raffaele Hospital, Milan, Italy

Nazli Dizman, The University of Texas MD Anderson Cancer Center, Houston, TX, USA

Maurizio Colecchia, Department of Pathology, IRCCS San Raffaele Hospital, Milan, Italy

Francesco De Cobelli, Department of Radiology, IRCCS San Raffaele Hospital, Milan, Italy

Alessandro Larcher, Department of Urology, IRCCS San Raffaele Hospital, Milan, Italy

Giuseppe Rosiello, Department of Urology, IRCCS San Raffaele Hospital, Milan, Italy

Alberto Briganti, Department of Urology, IRCCS San Raffaele Hospital, Milan, Italy

Francesco Montorsi, Department of Urology, IRCCS San Raffaele Hospital, Milan, Italy

Umberto Capitanio, Department of Urology, IRCCS San Raffaele Hospital, Milan, Italy

Philippe Spiess, Department of Genitourinary Oncology, H. Lee Moffitt Cancer Center and Research Institute, Tampa, FL, USA

Neeraj Agarwal, Huntsman Cancer Institute, University of Utah, Salt Lake City, UT, USA

Sumanta K. Pal, Department of Medical Oncology and Experimental Therapeutics, City of Hope Comprehensive Cancer Center, Duarte, CA, USA

Andrea Necchi, Department of Medical Oncology, IRCCS San Raffaele Hospital, Milan, Italy

Background: Immune-checkpoint inhibitors (ICIs), with or without tyrosine kinase inhibitors (TKIs), represent the backbone first-line treatment for patients with metastatic clear-cell renal cell carcinoma (mccRCC). While multiple ICI-based regimens are approved, limited data exist on the efficacy of ICI rechallenge, particularly regarding timing of rechallenge initiation. Methods: Using the TriNetX research database, we conducted a retrospective analysis of pts with mccRCC treated with ≥2 lines of ICI-based therapy across major international centers (2016–2024). Kaplan–Meier analysis was used to estimate progression-free survival (PFS) and overall survival (OS) following ICI rechallenge. Propensity score matching (PSM) was applied to adjust for age, sex, stage, metastatic sites, prior ICI type, and treatment sequencing strategy. Results: Among 6,737 patients with mccRCC, 288 (4.3%) received ≥2 lines of ICI. Median age was 63.8 years. The most common first-line regimens were nivolumab(N)+cabozantinib (44.1%), N+ipilimumab (26.5%), pembrolizumab(P)+axitinib (20%), and P+lenvatinib (8.4%). ICI rechallenge sequences were: N>N (47.6%), P>P (22.2%), N>P (17%), and P>N (13.2%). Median duration of prior ICI therapy was 19.5 months, and the median interval between ICI therapies was 8.91 months. After a median follow-up of 19.3 months, median OS following ICI rechallenge was 33.2 months, and median PFS was 8.5 months. Rechallenge ≥6 months after prior ICI was associated with improved PFS (8.8 vs. 5.2 months) and OS (34.9 vs. 19.4 months; p = 0.014). PSM confirmed that the OS benefit was independent of covariates (p = 0.03). Among patients with sarcomatoid features (n = 14), median OS was 35.1 months, with a median PFS of 6.56 months. Conclusions: Our findings suggest that ICI rechallenge may be effective regardless of prior immunotherapy, with longer treatment-free intervals (≥6 months) emerging as a key determinant of improved outcomes and a potential surrogate marker of response to be prospectively validated.

# Abstract: Contemporary assessment of risk stratification in ccRCC

**AUTHORS**

Aryaan Majumdar, University of Exeter, Exeter, Devon, UK

Theodore Poulton, Royal Devon and Exeter Hospital, Exeter, Devon, UK

Sarah Welsh, Royal Devon and Exeter Hospital, Exeter, Devon, UK

Thomas Dutton, Royal Devon and Exeter Hospital, Exeter, Devon, UK

Nicholas Campain, Royal Devon and Exeter Hospital, Exeter, Devon, UK

Background: Risk stratification for Renal Cell Carcinoma (RCC) is essential for surveillance and identifying patients for adjuvant immunotherapy (AIO). The Leibovich score (LS) is well established in clear cell RCC (ccRCC), but external validation studies were in historic cohorts prior to the AIO era. Predicting metastasis-free survival (MFS) in recent trial cohorts suggest recurrence risk may now be lower. We assessed recurrence rates in a real-world contemporary cohort. Methods: Retrospective evaluation of 526 consecutive patients undergoing nephrectomy in a UK tertiary centre (Oct 2020 – Nov 2025) confirmed 404 patients with RCC (n=329 clear cell). Histological data and follow-up were reviewed to record local (DFS) and distant (MFS) recurrence defined as per definitions from RECUR. Recurrence rates were compared against the predicted rates of the Leibovich score. Results: ccRCCs were stratified by LS into low (n=94), intermediate (n=107) and high (n=128) risk. Overall, 11% patients with ccRCC had metastatic recurrence and 13.1% (n=43) received AIO. At 1-year post-nephrectomy MFS was 100%, 96,7% and 80.0% in low, intermediate and high-risk groups respectively, compared with 99.5%, 90.4% and 57.7% in the original Leibovich cohort. At 3 years, corresponding MFS rates were 100%, 89.4% and 61.7%, in our cohort, versus 97.9%, 79.8% and 37.1% in the Leibovich cohort. Analysis by individual LS breakdown demonstrated an inverse relationship between risk score and time to recurrence, with medians of 731.5 days (LS 5), 365 days (LS 6), decreasing progressively to 174 days (LS 10). Conclusion: External validation of recurrence risk in a contemporary cohort of patients demonstrates early MFS exceeding that reported with risk stratification nomograms. In the AIO era more refined risk stratifications models are required to guide adjuvant treatment. Short timeframes to progressive disease for very high LS suggests potential undertreatment with single agent AIO.



# Abstract: Predictive Value of the Meet-URO and IMDC scores in metastatic renal cell carcinoma (mRCC) receiving 1st line immune-combinations (Meet-URO 33 study)

**AUTHORS**

Davide Bimbatti, Oncology 1 Unit, Istituto Oncologico Veneto IOV - IRCCS, Padua, Italy

Francesca Maines, Medical Oncology, Santa Chiara Hospital, Trento, Italy

Mariella Sorarù, U.O. Oncologia, Ospedale di Camposampiero, Camposampiero, Italy

Lucia Bonomi, Medical Oncology Unit, ASST Papa Giovanni XXIII, Bergamo, Italy

Emanuela Fantinel, Department of Oncology, Azienda Ospedaliera Universitaria Integrata di Verona, University of Verona, Verona, Italy

Annalisa Guida, Medical Oncology Unit, Santa Maria Hospital, Terni, Italy

Maria Giuseppa Vitale, Division of Oncology, Department of Oncology and Hematology, University Hospital of Modena, Modena, Italy

Sarah Scagliarini, Medical Oncology Unit, AORN Cardarelli Hospital, Naples, Italy

Cecilia Nasso, Medical Oncology, Ospedale Santa Corona, Pietra Ligure, Italy

Filippo Maria Deppieri, Medical Oncology Unit, AULSS 3 Serenissima, Mestre-Venice, Italy

Brigida Anna Maiorano, Department of Medical Oncology, IRCCS San Raffaele Hospital, Milan, Italy

Claudia Caserta, Oncology Unit, Santa Maria della Misericordia Hospital, Perugia, Italy

Alessia Cavo, Oncology Unit, ASL3 - Villa Scassi Hospital, Genoa, Italy

Arianna Dri, Department of Medical Oncology, Centro di Riferimento Oncologico di Aviano CRO-IRCCS, Aviano, Italy

Francesca La Russa, 15. Department of Oncology, Ospedale San Bortolo, Vicenza, Italy

Vincenza Conteduca, Unit of Medical Oncology and Biomolecular Therapy and CREATE Center for Research and Innovation in Medicine, Department of Medical and Surgical Sciences, University of Foggia, Policlinico Riuniti, Foggia, Italy

Silvia Zai, Medical Oncology Unit, Azienda Ospedaliera SS. Antonio e Biagio e Cesare Arrigo, Alessandria, Italy

Francesco Pierantoni, Medical Oncology Unit, Azienda Ospedaliera SS. Antonio e Biagio e Cesare Arrigo, Alessandria, Italy

Fabrizio Di Costanzo, Department of Medical Oncology, Università degli Studi di Napoli Federico II, Naples, Italy

Sara Elena Rebuzzi, Medical Oncology Unit 2, Ospedale Molinette, Azienda Ospedaliero-Universitaria Città della Salute e della Scienza di Torino, Turin, Italy

Background: The prognostic IMDC and Meet-URO scores are well-established in untreated/pretreated mRCC patients. However, their predictive performance with 1st line immune-combinations is still unclear. Methods: We conducted an Italian multicenter retrospective-prospective observational study of mRCC patients receiving 1st line ICI–ICI or ICI–TKI. Overall (OS) and progression-free survival (PFS) were estimated using the Kaplan–Meier method. The predictive value of the Meet-URO and the IMDC scores was assessed using Cox proportional hazards models including treatment-by-risk interaction. Results: 1511/1907 enrolled patients were assessable. After a mFU of 27.8 months, in the overall population, in the ICITKI (76%) and ICI-ICI (24%) groups: mOS was 37.8, 39.0 and 37.8 months, respectively; PFS was 17.9, 19.4 and 13.1 months, respectively. Hazard ratios for ICI– TKI vs ICI–ICI were consistent across Meet-URO and IMDC groups for OS and PFS and no significant treatment-by-risk interaction was observed for both prognostic scores (Table). Conclusions: In this large real-world study, Meet-URO and IMDC prognostic scores did not show predictive value for selecting 1st line immune-combinations ICI-ICI vs ICI-TKI. Therefore, other factors should be considered for guiding treatment choice (e.g. metastatic sites, tumor burden).



# Abstract: Tivozanib Monotherapy as First-Line Treatment in Intermediate-Risk Metastatic Renal Cell Carcinoma: TIVOREAL-SOGUG, a Spanish Real-World Experience

**AUTHORS**

Enrique Gallardo, Hospital Universitari Parc Taulí, Sabadell, Barcelona, Spain

Begoña Pérez-Valderrama, Hospital Universitario Virgen del Rocío, Sevilla, Spain

Inmaculada Aparicio, Hospital General Universitario Gregorio Marañón, Madrid, Spain

Laura López, Hospital Universitario de León, León, Spain

Blanca Távara, Hospital Universitario Lucus Augusti, Lugo, Spain

Lucía Oliva, Hospital Universitario Virgen de la Victoria, Málaga, Spain

María José Méndez-Vidal, Maimonides Institute for Biomedical Research of Córdoba (IMIBIC), Hospital Universitario Reina Sofía (HURS), Medical Oncology Department, Córdoba, Spain

Guillermo Crespo, Hospital Universitario de Burgos, Burgos, Spain

Javier Gavira, Institut Català d'Oncologia, L’Hospitalet, Barcelona, Spain

Aránzazu González-del-Alba, Hospital Universitario Puerta de Hierro-Majadahonda, Madrid, Spain

Cristina Pernaut, Hospital Universitario Severo Ochoa, Madrid, Spain

Miguel Ángel Climent, Instituto Valenciano de Oncología, Valencia, Spain

Imanol Martínez, Hospital Universitario Fundación Jiménez Díaz, Madrid, Spain

Cristina Suárez, Hospital Universitario Vall d'Hebron, Barcelona, Spain

Victoria Tirado, Hospital General Universitario Gregorio Marañón, Madrid, Spain

Cristina López, Hospital General Universitario Gregorio Marañón, Madrid, Spain

Ángel Rodríguez, Hospital Universitario de León, León, Spain

Alejandro José Barroso, Hospital Universitario Virgen del Rocío, Sevilla, Spain

Eduardo Zamorano, Hospital Universitario Virgen de la Victoria, Málaga, Spain

Sergio Vázquez, Hospital Universitario Lucus Augusti, Lugo, Spain

BACKGROUND: Tivozanib is a selective VEGFR tyrosine kinase inhibitor approved for first-line (1L) treatment of metastatic renal cell carcinoma (mRCC). Real-world data in IMDC intermediate-risk (IR) patients remain limited. This study evaluated tivozanib effectiveness and safety of in routine clinical practice.

METHODS: TIVOREAL-SOGUG is a multicenter, retrospective, real-world observational study of patients with clear-cell mRCC receiving 1L tivozanib (2017-2024) at 14 Spanish oncology centers of the Spanish Genitourinary Oncology Group (SOGUG). Primary endpoint: progression-free survival (PFS). Secondary endpoints: objective response rate (ORR), overall survival (OS) and safety. Survival was estimated using Kaplan-Meier methodology; tumor response per RECIST 1.13. Post hoc exploratory analysis compared IR patients with 1 or 2 IMDC risk factors (RF).

RESULTS: Of 198 evaluable patients, 100 (50.5%) had IMDC-IR disease: 60 with 1 RF and 40 with 2 RF. Baseline characteristics were balanced except for gender (37.5% female in 2 RF vs. 18.3% in 1 RF) and ECOG performance status (71.1% ECOG >1 in 2 RF vs. 11.6% in 1 RF). Previous nephrectomy: 43.5% in 2 RF; 18.3% in 1 RF. Median follow-up: 44.6 months (95% CI 39.3 – 49.9). Efficacy outcomes were comparable between subgroups and consistent with the overall population (Table 1). Tolerability was favorable: 42.0% maintained full dose, 13.0% required dose reduction, and 9.0% discontinued tivozanib due to toxicity, with no differences between subgroups. Among patients discontinuing 1L tivozanib (n=79), 64.5% received subsequent treatment.

CONCLUSIONS: First-line tivozanib demonstrated clinically meaningful effectiveness and acceptable tolerability in IMDC-IR mRCC. Durable disease control was observed across patients with 1 or 2 RF, with comparable outcomes between subgroups and similar to the overall population. These real-world data support tivozanib as a viable option for IR mRCC, regardless of the number of associated RF.



# Abstract: Local treatment of bone metastases in renal cell cancer: a retrospective study

**AUTHORS**

EW Dootjes, Erasmus Medical Center, Rotterdam, Netherlands

DLM van Broekhoven, Leiden University Medical Center, Leiden, Netherlands

A Joosse, Erasmus Medical Center, Rotterdam, Netherlands

KAT Naipal, Erasmus Medical Center, Rotterdam, Netherlands

EAM Froklage, Erasmus Medical Center, Rotterdam, Netherlands

E Oomen-de Hoop, Erasmus Medical Center, Rotterdam, Netherlands

PK Bos, Erasmus Medical Center, Rotterdam, Netherlands

AAM van der Veldt, Erasmus Medical Center, Rotterdam, Netherlands

Abstract Background: Since systemic treatments have improved the overall survival (OS) for patients with metastatic renal cell cancer (mRCC), an increasing number of these patients are diagnosed with bone metastases (BMs). Local treatment of BMs is often required because these metastases significantly impact mobility and quality of life. The objective was to investigate OS in patients with different local treatments of BM. Methods: A single-center retrospective cohort study was conducted in a tertiary referral center. All patients with mRCC and BMs were included. Local treatment was defined as local aggressive therapy (i.e. surgery or radiotherapy with remineralization regimen) or local low-dose radiotherapy (i.e. radiotherapy with nonremineralization regimen). Primary endpoint was OS, defined as time from first local treatment of BM, according to different local therapies. Results: A total of 189 mRCC patients with BMs were included. At time of mRCC diagnosis, 51.9% of patients presented with BMs. Of 189 patients, 81% developed skeletal-related events (SRE) and 141 (74.6%) patients received local treatment for BMs. Of these 141 patients, first local treatment of BMs consisted of a non-remineralization radiotherapy regimen (n=74), surgery (n=34), or a remineralization radiotherapy regimen (n=20). A total of 66 patients had multiple local treatments. Median time from diagnosis of first BM to SRE was 1.4 months (IQR=0.3-10.2). Median time from diagnosis of mRCC to local aggressive therapy and local low-dose radiotherapy was 1.2 (IQR=0.1-7.2) and 5.2 (IQR=0.9-14.9) months, respectively. Local aggressive therapy was associated with an improved median OS as compared to local low-dose radiotherapy [33.0 months (IQR=13.4-63.4) versus 6.0 months (IQR=2.3-51.8), p<0.0001]. Conclusions: Patients with mRCC and local aggressive therapy of BMs had a better OS compared to patients with local low-dose radiotherapy of BMs. These findings underscore the need for a multidisciplinary approach to optimize the local treatment for individual patients with mRCC and BMs.

# Abstract: Defining Outcomes of Sequential Therapies Incorporating Cabozantinib (CABO) as Second Line in Metastatic Renal Cell Carcinoma (mRCC)

**AUTHORS**

Gloria Messina, Università Cattolica del Sacro Cuore, Rome, Italy

Chiara Ciccarese, Department of Medical Oncology, Fondazione Policlinico Universitario A. Gemelli IRCCS, Rome, Italy

Romina Rose Pedone, Università Cattolica del Sacro Cuore, Rome, Italy

Alessandro Scala, Università Cattolica del Sacro Cuore, Rome, Italy

Daniela Arduini, Department of Medical Oncology, Fondazione Policlinico Universitario A. Gemelli IRCCS, Rome, Italy

Denis Occhipinti, Università Cattolica del Sacro Cuore, Rome, Italy

Paola Troisi, Università Cattolica del Sacro Cuore, Rome, Italy

Maria Antonia Fucile, Università Cattolica del Sacro Cuore, Rome, Italy

Rexhina Ajdhoni, Università Cattolica del Sacro Cuore, Rome, Italy

Chiara Ligato, Università Cattolica del Sacro Cuore, Rome, Italy

Valeria Sardaro, Università Cattolica del Sacro Cuore, Rome, Italy

Luigi Roca, Università Cattolica del Sacro Cuore, Rome, Italy

Alessio Neri, Università Cattolica del Sacro Cuore, Rome, Italy

Davide Di Leo, Università Cattolica del Sacro Cuore, Rome, Italy

Rachele Belletto, Università Cattolica del Sacro Cuore, Rome, Italy

Fabiana Caliciotti, Università Cattolica del Sacro Cuore, Rome, Italy

Andrea Missimei, Università Cattolica del Sacro Cuore, Rome, Italy

Nair Nudrat Syeda, Università Cattolica del Sacro Cuore, Rome, Italy

Federica Colasanti, Università Cattolica del Sacro Cuore, Rome, Italy

Giampaolo Tortora, Department of Medical Oncology, Fondazione Policlinico Universitario A. Gemelli IRCCS, Rome, Italy

Roberto Iacovelli, Department of Medical Oncology, Fondazione Policlinico Universitario A. Gemelli IRCCS, Rome, Italy

Immunotherapy (IO)-based combinations, including dual immune checkpoint inhibition (IO–IO) or IO plus VEGFR-TKI (IO–TKI), represent the standard first-line (1L) treatment for mRCC. The CONTACT-03 trial demonstrated the activity of CABO in the second line (2L), without differences according to prior therapy. We evaluated whether the type of 1L treatment influences the efficacy of 2L CABO and outcomes of the entire treatment sequence. We retrospectively analyzed mRCC patients treated at our institution with 2L CABO following IO-based combinations. The primary endpoint was median overall survival (mOS) from the start of 1L. Secondary endpoints included median PFS2 (from 1L initiation to progression on 2L CABO), efficacy according to 1L regimen, and IMDC risk groups. Between November 2018 and February 2025, 46 patients received 2L CABO (22 after IO– IO, 24 after IO–TKI). Median age was 61.9 years; 72% were male; 76% had prior nephrectomy; 89% had clear-cell histology; and 33% had metastatic disease at diagnosis. IMDC risk was favorable in 11%, intermediate in 80%, and poor in 9%. After a median follow-up of 29.7 months, 35 patients (76%) progressed to 2L CABO and 20 (43%) died. Median OS was 31.9 months, with no significant difference between IO–IO and IO–TKI (not reached vs 27.0 months; p=0.30). In intermediate–poor risk patients, mOS was similar (31.9 vs 27.1 months; p=0.48). Median PFS on 2L CABO was 9.6 months (95% CI, 7.3–11.9). Median PFS2 was 22.7 months and did not differ by 1L regimen (24.0 vs 20.0 months; p=0.98). CABO retains consistent activity as 2L therapy after IO-based combinations, regardless of prior regimen. Further studies are needed to define optimal sequencing, particularly when CABO is used earlier.

# Abstract: Real world effectiveness of first-line cabozantinib and nivolumab in advanced renal cell carcinoma: multicentre experience from the United Kingdom

**AUTHORS**

Ilesha Ewart, Beatson West of Scotland Cancer Centre, Glasgow, UK

Jack Leach, Edinburgh Cancer Centre, Edinburgh, UK

Harisha Shivamoggi, Beatson West of Scotland Cancer Centre, Glasgow, UK

Sarah Buchanan, Northern Ireland Cancer Centre, Belfast, UK

Ruba Hamed, Northern Ireland Cancer Centre, Belfast, UK

Alison Clayton, Northern Ireland Cancer Centre, Belfast, UK

Caroline Forde, Northern Ireland Cancer Centre, Belfast, UK

Mark Stares, Edinburgh Cancer Centre, Edinburgh, UK

Jennifer Allison, Edinburgh Cancer Centre, Edinburgh, UK

Jahangeer Malik, Edinburgh Cancer Centre, Edinburgh, UK

Kyriakos Ioannou, Beatson West of Scotland Cancer Centre, Glasgow, UK

Sarah Slater, Beatson West of Scotland Cancer Centre, Glasgow, UK

Manreet Randhawa, Beatson West of Scotland Cancer Centre, Glasgow, UK

Balaji Venugopal, Beatson West of Scotland Cancer Centre, Glasgow, UK

Introduction: Combination immune checkpoint inhibitors and vascular endothelial growth factor receptor tyrosine kinase inhibitors have changed the treatment paradigm in advanced renal cell carcinoma (RCC). Cabozantinib combined with nivolumab (cabo/nivo) is approved as first-line treatment for RCC. We aimed to assess real world experience of cabo/nivo at three large cancer centres in the UK. Method: We performed a retrospective review of medical records of patients (pts) receiving cabo/nivo from 1st October 2021 until 1st September 2025. Time to treatment failure (TTTF), defined as time to subsequent therapy, was considered a surrogate for progression free survival (PFS). Overall response rate (ORR) was as documented by clinicians. Overall survival (OS) and TTTF was estimated by Kaplan Meier method. Results: Of 184 pts treated, 69% were male and 31% female; median age 65 (18-85 years). At baseline, 33% were WHO performance status (PS) 0, 52% PS1, 14% PS2 and 1% PS3. As per International Metastatic RCC Database Consortium (IMDC) score, 16% had favourable risk disease, 50% intermediate and 34% poor. 141 (77%) pts had clear cell RCC, 43 (23%) non-clear cell RCC and 14 (8%) sarcomatoid features. At baseline, 117 (64%) pts had lung, 67 (36%) bone, 34 (18%) liver, and 26 (14%) brain metastases. Median TTTF and OS was 19.24 and 26.11 months respectively. ORR was 58% and 27 (15%) pts had stable disease. 44 (24%) pts had a grade ≥3 adverse event (AE) related to either cabozantinib or nivolumab. Immune-mediated AE occurred in 98 (53%) pts; 53 (29%) required steroid treatment and 11 (6%) further immunosuppressants. Cabozantinib dose was reduced in 102 (55%) pts. Conclusion: Cabo/nivo has robust clinical efficacy in real word experience, including in patients underrepresented in clinical trials. ORR and PFS was comparable with outcomes reported in clinical trials with a manageable safety profile.

# Abstract: Population characteristics and treatment choice for patients with metastatic renal cell carcinoma treated at Guy’s Cancer Centre

**AUTHORS**

Jack Brophy, King's College Hospital NHS Foundation Trust, London, UK

Danielle Crawley, Guy's Cancer Centre, London, UK

Sarah Rudman, Guy's Cancer Centre, London, UK

Kate Smith, Guy's Cancer Centre, London, UK

Deborah Enting, Guy's Cancer Centre, London, UK

Background: In the last decade outcomes for patients with metastatic Renal Cell Carcinoma (mRCC) have been transformed by the introduction of combination immunotherapy (ipilimumab/nivolumab) and combination immunotherapy with tyrosine kinase inhibitors. However, the UK National Kidney Cancer Audit (2023) highlighted large geographical discrepancies in those receiving first line systemic anti cancer therapy (SACT). Across Hospital trusts in the United Kingdom this ranged from 9-87% of patients. Here we present treatment data from a single London tertiary centre. Methods: The electronic patient records of 71 patients with a diagnosis of renal cell carcinoma treated at our trust over the last 10 years were used to collect data on patient demographics, tumour characteristics, and lines of treatment. Approval was obtained from the Trust’s Research and Development department. Results: The patient demographic data showed a mean age of 67 years with a mean age at diagnosis of 59 years. The population was 68% male and 32% female. First line treatment in the metastatic setting is detailed in Table 1. Conclusion: These data provide a retrospective illustration of the population characteristics of patients with mRCC and the SACT used in the first line setting. The percentage of metastatic patients who receive first line SACT at Guy’s Cancer Centre (96%) compares favourably to the nationwide median of 48.3% identified by the National Kidney Cancer Audit (2023), and is similar to the GSTT specific percentage identified by the audit (78%). The regional variation identified in this audit raises concerns that certain populations within the UK are being under treated. The underlying causes are unclear.



# Abstract: Real world efficacy and treatment experiences of cabozantinib plus nivolumab (cabo-nivo) in metastatic renal cell carcinoma (mRCC): a REFINE analysis

Jeffrey Zhong, Division of Solid Tumor Oncology, University Hospitals Cleveland Medical Center, Case Western Reserve University School of Medicine, Cleveland, OH, USA

Seunghee Margevicius, Department of Population Quantitative Health Sciences, Case Western Reserve University, Cleveland, OH, USA

Abby Grier, Division of Solid Tumor Oncology, University Hospitals Cleveland Medical Center, Case Western Reserve University School of Medicine, Cleveland, OH, USA

Michael Zimmerman, Department of Medical Oncology, Thomas Jefferson Sidney Kimmel Cancer Center, Philadelphia, PA, USA

Ahmet Yildrim, Genitourinary Medical Oncology Program, Department of Hematology and Medical Oncology, Winship Cancer Institute of Emory University, Atlanta, GA, USA

Joannah Jung, Division of Hematology-Oncology, University of California San Diego Moores Cancer Center, La Jolla, CA, USA

Mark Chang, Department of Solid Tumor Oncology, Atrium Health Wake Forest Baptist Comprehensive Cancer Center, Charlotte, NC, USA

Nagendra Dhanikonda, Department of Hematology-Oncology, Fox Chase Cancer Center, Philadelphia, PA, USA

Alex Eng, Division of Oncology, Medical College of Wisconsin, Milwaukee, WI, USA

Sourat Darabi, Hoag Family Cancer Institute, Newport Beach, CA, USA

Khaldoune Hamade, Department of Medical Oncology, Thomas Jefferson Sidney Kimmel Cancer Center, Philadelphia, PA, USA

Bashar Abuqayas, Division of Oncology, Holden Comprehensive Cancer Center, Iowa City, IA, USA

Deepak Kilari, Division of Oncology, Medical College of Wisconsin, Milwaukee, WI, USA

Pooja Ghatalia, Department of Hematology-Oncology, Fox Chase Cancer Center, Philadelphia, PA, USA

Claud Grigg, Department of Solid Tumor Oncology, Atrium Health Wake Forest Baptist Comprehensive Cancer Center, Charlotte, NC, USA

Mehmet Asim Bilen, Genitourinary Medical Oncology Program, Department of Hematology and Medical Oncology, Winship Cancer Institute of Emory University, Atlanta, GA, USA

Kevin Zarrabi, Department of Medical Oncology, Thomas Jefferson Sidney Kimmel Cancer Center, Philadelphia, PA, USA

Pingfu Fu, Department of Population Quantitative Health Sciences, Case Western Reserve University, Cleveland, OH, USA

Rana McKay, Division of Hematology-Oncology, University of California San Diego Moores Cancer Center, La Jolla, CA, USA

Pedro Barata, Division of Solid Tumor Oncology, University Hospitals Cleveland Medical Center, Case Western Reserve University School of Medicine, Cleveland, OH, USA

Background: Cabo-nivo is a standard first-line regimen for mRCC, but real-world data describing treatment delivery, tolerability, and effectiveness remains limited. In this study, we prospectively evaluated real-world clinical outcomes and treatment experiences with cabo-nivo. Methods: Patients with mRCC treated with cabo-nivo between September 1st, 2023 and January 1st, 2026 in the multi-site REFINE consortium (Zhong et al, Oncologist 2025) were analyzed. Baseline disease and patient characteristics were collected. Real-world treatment metrics included dose reductions, duration of therapy, steroid use, and hospitalizations. Efficacy outcomes included investigator-assessed objective response rate (ORR) using RECIST principals, real world progression-free survival (PFS), and overall survival (OS), measured from cabo-nivo initiation. Results: A total of 83 patients from 8 academic centers were included (median age 63.5 years; 73.3% male; clear cell 63.3%; sarcomatoid differentiation 6%; IMDC favorable/intermediate/poor risk 20.5%/63.9%/14.5%). 60 patients had no prior treatment exposure, while 23 patients had prior treatment exposure to IO-IO, IO-TKI, TKI monotherapy, or adjuvant pembrolizumab. With median follow-up 23.1 months, median treatment duration was 9.46 months. Median PFS and OS were 27.3 months (95% CI: 16.22-33.96) and 31.6 months (95% CI: 22.62-42.89), respectively. OS was significantly prolonged in clear cell subtype (HR 0.49, 95% CI 0.25-0.97, p = 0.04). Upfront dose reductions occurred in 3.6% of patients, on-treatment dose reductions in 33.7%, and permanent discontinuation in 41.0% (41.2% due to treatment related adverse event). Corticosteroid use rate was 24.1% (85% high dose) and 14.5% of patients experienced treatment-related hospitalizations, most commonly from pneumonitis (25%). Conclusion: In this multicenter real-world analysis, cabo-nivo showed clinically meaningful effectiveness with expected dose modifications consistent with a real world population. These findings complement clinical trial data and provide important context for real-world delivery of cabo-nivo.

# Abstract: Ablation and Surgery Show Comparable Long-Term Outcomes for T1a Renal Cell Carcinoma: Nationwide Danish Data

**AUTHORS**

Jesper Jespersen, Department of Molecular Medicine, Aarhus University, Aarhus, Denmark

Johanne Ahrenfeldt, Department of Molecular Medicine, Aarhus University, Aarhus, Denmark

Pernille Tonnesen, Department of Molecular Medicine, Aarhus University, Aarhus, Denmark

Tommy Nielsen, Department of Urology, Aalborg University Hospital, Aalborg, Denmark

Anna Krarup Keller, Department of Urology, Aarhus University Hospital, Aarhus, Denmark

Laura Iisager, Department of Molecular Medicine, Aarhus University, Aarhus, Denmark

Iben Lyskjær, Department of Molecular Medicine, Aarhus University, Aarhus, Denmark

Background: Incidental detection of small renal masses has increased early-stage RCC diagnoses. Ablation is a minimally invasive alternative to surgery for T1a tumors (<4 cm), but long-term oncologic outcomes are debated. Methods: We linked nationwide Danish registries to identify adults with T1a RCC diagnosed from 2013– 2021 (n = 3,094). After exclusions (n = 1,232), 1,862 patients treated with ablation, resection, or nephrectomy were included. Outcomes compared with treatment procedure included length of stay, 30-day hospital contacts, local recurrence, and distant metastasis. Progression (distant metastasis or local recurrence) was evaluated using Fine-Gray competing-risk regression adjusted for age, sex, comorbidity, morphology, tumor size, and grade. Results: Use of ablation increased from 9% in 2004 to 32% in 2021, with marked regional variation (5-47% in 2013-2021). Median hospital stay was 0 days after ablation versus 2 days after surgery (p<0.001), and fewer ablation patients had any 30-day hospital contact (72% vs 90%; p<0.001). Five-year local recurrence rates were higher after ablation than after resection (2.4% vs 1.2%; p = 0.007), with a trend toward earlier recurrence (median 635 vs 1,447 days; p = 0.073). These recurrences were typically managed with repeat ablation or surgery. Distant metastasis rates were similar after ablation and resection (1.7% vs 1.9%), and adjusted risk of progression (metastasis or local recurrence) did not differ (HR 1.46, 95% CI 0.60–3.56; p=0.40). Conclusions: In the nationwide cohort, ablation for T1a RCC was associated with shorter hospitalization and fewer early contacts, while metastatic risk was comparable to resection. Local recurrence was more frequent after ablation, but was manageable with additional surgery or ablation. Limitations include missing tumor size for ablation cases and incomplete grading data.

# Abstract: Activity of frontline immune checkpoint inhibitor (ICI)-based therapy in metastatic renal cell carcinoma (mRCC) with rhabdoid features

**AUTHORS**

Macarena Rey-Cardenas, Department of Medical Oncology, Gustave Roussy, Paris Saclay University, Villejuif, France

Javier Gavira, Department of Medical Oncology, Institut Català d’Oncologia L’Hospitalet, L’Hospitalet de Llobregat, Barcelona, Spain

Antía Iglesias-Beiroa, Department of Medical Oncology, Hospital Universitario de La Princesa, Madrid, Spain

Arantxa Agulleiro-Pérez, Department of Medical Oncology, Institut Català d’Oncologia L’Hospitalet, L’Hospitalet de Llobregat, Barcelona, Spain

Maria Pané-Foix, Department of Pathology, Hospital Universitari de Bellvitge-IDIBELL, L’Hospitalet de Llobregat, Barcelona, Spain

Nathalie Rioux-Leclercq, Department of Pathology, University Hospital of Rennes, Rennes, France

Mariangela Calabrese, Department of Medical Oncology, Gustave Roussy, Paris Saclay University, Villejuif, France

Luigi Roca, Department of Medical Oncology, Gustave Roussy, Paris Saclay University, Villejuif, France

Natacha Naoun, Department of Medical Oncology, Gustave Roussy, Paris Saclay University, Villejuif, France

Bernard Escudier, Department of Medical Oncology, Gustave Roussy, Paris Saclay University, Villejuif, France

Ronan Flippot, Department of Medical Oncology, Gustave Roussy, Paris Saclay University, Villejuif, France

Laurence Albiges, Department of Medical Oncology, Gustave Roussy, Paris Saclay University, Villejuif, France

Background: Metastatic renal cell carcinoma (RCC) with sarcomatoid and/or rhabdoid dedifferentiation typically exhibits aggressive biological behaviour and poor prognosis. While sarcomatoid RCC (sRCC) shows compelling response to immune checkpoint inhibitors (ICI), outcomes of rhabdoid RCC (rRCC) on ICI are unknown. Methods: We retrospectively reviewed patients with rRCC, sRCC, mixed s/rRCC and grade 4 tumors with no sRCC or rRCC features (g4RCC) treated with frontline ICI combinations, either dual ICI or ICI plus VEGFR tyrosine kinase inhibitor (TKI). Endpoints were objective response rate (ORR), defined as the proportion of patients achieving complete or partial response, progression-free survival (PFS) and overall survival (OS). Results: Overall, 98 patients were included, 86 (88%) with clear-cell and 12 (12%) with non-clear-cell RCC. As per IMDC score, 6 (6%), 55 (56%), and 37 (38%) patients had favorable, intermediate and poor risk disease, respectively. The cohort consisted of 33 (34%) rRCC, 26 (27%) sRCC, 25 (26%) s/rRCC, and 14 (14%) g4RCC patients. The distribution of treatment modalities (dual ICI vs. ICI+TKI) was balanced across groups, with around 70% dual ICI use in rRCC, sRCC and s/rRCC groups. With a median follow-up time of 31.2 months (mo), the median PFS for rRCC, sRCC, s/rRCC and g4RCC were 27.3, 4.6, 21.5 and 11.7 mo, respectively (p=0.08). For patients on dual ICI, PFS was 31.8 mo in rRCC (95% CI: 11.2-NA) compared to 3.5 mo in sRCC (95% CI: 2.3-NA) (p = 0.05). No significant difference was found for OS (p = 0.42). Patients with rRCC had the highest ORR (67%), followed by s/rRCC (60%), g4RCC (58%), and sRCC (40%). Histology was significantly associated with response (Fisher p = 0.04), with lower odds of response in sRCC (OR 0.32). Conclusions: ICI-based regimens showed significant activity in patients with rRCC, presenting a higher ORR and PFS compared to patients with sRCC, supporting their use as an effective treatment for this subpopulation. sRCC is associated with a poorer prognosis, despite the use of dual ICI.

# Abstract: Real-world assessment of clinical stage, pathological stage, and disease-free survival in clear cell renal cell carcinoma to inform neoadjuvant trial inclusion criteria

**AUTHORS**

Mouhamad Ismail, Cambridge University Hospital, Cambridge, UK

James Blackmur, Urology Department, NHS Lothian, Edinburgh Institute of Genetics and Cancer, University of Edinburgh, Edinburgh, UK

Hossameldin Abdallah, Cambridge University Hospital, Cambridge, UK

Se-Eun Kim, Dana-Farber Cancer Institute-ECOG-ACRIN Biostatistics Center, Boston, MA, USA

Ahmed Anber, Cambridge University Hospital, Cambridge, UK

Neus Costafreda-Fu, University of Cambridge, Cambridge, UK

Teele Kuusk, Cambridge University Hospital, Cambridge, UK

Alex Laird, Urology Department, NHS Lothian, Edinburgh Institute of Genetics and Cancer, University of Edinburgh, Edinburgh, UK

Sabrina Rossi, University of Cambridge, Cambridge, UK

Rebecca Sen, Cambridge University Hospital, Cambridge, UK

Samara Shahjahan, University of Cambridge, Cambridge, UK

Sarah Welsh, Royal Devon University Hospitals NHS Foundation Trust, Exeter, UK

Naomi Haas, Abramson Cancer Center, Perelman School of Medicine, University of Pennsylvania, Philadelphia, PA, USA

Axel Bex, Royal Free London NHS Foundation Trust, London, UK

Grant D. Stewart, University of Cambridge, Cambridge, UK

James O. Jones, University of Cambridge, Cambridge, UK

Background: Assignment to adjuvant immunotherapy for renal cell carcinoma (RCC) depends on histopathological assessment of the resected tumour, information unavailable in a neoadjuvant trial. Estimating relapse risk using only pre-surgical MDT data is therefore essential for the design of neoadjuvant studies. Methods: We evaluated the relationship between pre-surgical characteristics: clinical stage and CT-measured tumour size and post-surgical characteristics: pathological stage, and disease-free survival (DFS) in patients with clear cell RCC without distant metastases who underwent surgery at two UK tertiary centres (Cambridge [ARTIST Study NCT04060537] and Edinburgh). TNM 8th Edition criteria were applied. DFS was analysed using Kaplan–Meier curves, log-rank tests, and Cox proportional hazards models. Results: 736 patients were included. Patients with cT1–2 disease had significantly better DFS than those with cT3–4 (p < 0.0001; HR 3.45, 95% CI 2.49–4.79). Up-staging to pT3+ occurred in 44.6% of cT1–2 patients, whereas only 3.2% of cT3 patients were down-staged (figure 1). Among cT1–2 patients, those up-staged to pT3+ had substantially worse DFS than those remaining pT1–2 (p < 0.0001; HR 5.13, 95% CI 3.15–8.36). Up-staged tumours were significantly larger (median 6.5 cm (IQR 5-8.3) vs 4.0 cm (IQR 3-5.6); p < 0.0001). A size threshold also had value: in the whole population, tumours <7 cm were associated with significantly better DFS (p < 0.0001; HR 2.57, 95% CI 1.83–3.53). Conclusions: Baseline cT3 stage reliably identifies patients with high risk pT3 disease. Up-staging from cT1-2 and tumour size threshold is common and linked to poorer DFS, further work is needed to identify this subset before surgery or neoadjuvant therapy. Further UK international data expansion are underway to refine predictive models.



# Abstract: Cabozantinib plus nivolumab as first-line treatment for advanced renal cell carcinoma: final results of the prospective real-world CaboCombo study

**AUTHORS**

Philippe Barthélémy, Medical Oncology Department, Hôpitaux Universitaires de Strasbourg, Strasbourg, France

Pierre Gimel, Centre d'Urologie Site Médipole – Centre Catalan d'Urologie, Cabestany, France

Hakim Mahammedi, Department of Medical Oncology, Centre Jean Perrin, Clermont-Ferrand, France

Lionnel Geoffrois, Institut de Cancérologie de Lorraine – Alexis Vautrin, Vandoeuvre-lès-Nancy, France

Morgan Goujon, Centre Hospitalier Universitaire de Besançon – Jean Minjoz, Besançon, France

Carolina Saldana, Centre Hospitalier Universitaire Henri Mondor, Créteil, France

Sébastian Bailly, Centre Hospitalier Universitaire de Clermont-Ferrand – Gabriel-Montpied, Clermont-Ferrand, France

Dimitrios Dionysopoulos, Department of Medical Oncology, Papageorgiou Hospital, Aristotle University of Thessaloniki, School of Health Sciences, Faculty of Medicine, Thessaloniki, Greece

Marilena di Napoli, Department of Urology and Gynaecology, Istituto Nazionale Tumori IRCCS Fondazione G. Pascale, Naples, Italy

Diego Teyssonneau, Department of medical Oncology, Institut Bergonié, Bordeaux, France

Vanessa Barue, Ipsen, Paris, France

Valérie Perrot, Ipsen, Paris, France

Pierre Bigot, Department of Urology, Centre Hospitalier Universitaire d’Angers, Angers, France

Cabozantinib plus nivolumab (CaboNivo) is an approved first-line (1L) treatment for advanced renal cell carcinoma (aRCC), based on the phase 3 CheckMate 9ER trial which demonstrated significantly improved outcomes versus sunitinib. CaboCombo (NCT05361434) assessed the real-world effectiveness and tolerability of 1L CaboNivo in patients with aRCC, including populations not represented in CheckMate 9ER.

Methods: CaboCombo is a phase 4, prospective, international, observational study of consecutively-recruited adults with clear-cell aRCC treated with 1L CaboNivo in real-world clinical practice. The primary endpoint was 18-month real-world overall survival (OS) rate from CaboNivo initiation. Secondary endpoints included real-world progression-free survival (rwPFS), best overall response, overall response rate (rwORR), disease control rate, time to response, duration of response and safety. Here, we report final study data.

Results: Among 311 included patients (median age, 67.0 years; 72.3% male), 12.5% had ECOG performance status >1, 93.5% had metastatic disease (bone, 35%; brain, 7%) and 57.2% had prior nephrectomy. Known IMDC risk (n=204) was favorable, intermediate and poor for 20.1%, 54.4% and 25.5% of patients, respectively. Median CaboNivo treatment duration was 15.2 (range, 0.1–34.9) months and median follow-up was 19.7 (range, 0.1–35.3) months. The primary endpoint of 18-month OS rate was 74% (95% confidence interval [CI], 69–79%). Median rwPFS was 14.0 months (95% CI, 12.2–16.0) and rwORR was 62.1% (95% CI, 56.2–67.8). Median dose of cabozantinib was 25.95 mg/day; TEAEs were experienced by 96.5% and SAEs by 44.4% of patients. No new safety signals were identified. Effectiveness and safety data are reported in the table.

Conclusions: Results of this prospective real-world study are consistent with CheckMate 9ER results, including a more frail and elderly population, supporting the use of 1L CaboNivo in aRCC.



# Abstract: CURE-RCC: Utilisation and Real-World Effectiveness of Management Strategies in non-metastatic Renal Cell Carcinoma

**AUTHORS**

Rasmus D. Petersson, Department of Urology, Zealand University Hospital, Roskilde, Denmark

Tommy Kjærgaard Nielsen, Department of Urology, Aalborg University Hospital, Aalborg, Denmark

Mia Gebauer Madsen, Department of Urology, Aarhus University Hospital, Aarhus, Denmark

Katrine Schou-Jensen, Department of Urology, Herlev-Gentofte Hospital, Copenhagen, Denmark

Michael Porath, Department of Urology, Lillebaelt Hospital, Vejle, Denmark

Anne Kirstine Møller Darras, Department of Oncology, Herlev-Gentofte Hospital, Copenhagen, Denmark

Niels Fristrup, Department of Oncology, Aarhus University Hospital, Aarhus, Denmark

Thomas Bretlau, Department of Radiology,

Herlev-Gentofte Hospital, Copenhagen, Denmark

Philip Hedegaard, Department of Urology, Zealand University Hospital, Roskilde, Denmark

Lars Lund, Department of Urology, Odense University Hospital, Odense, Denmark

Frederik F. Thomsen, Department of Urology, Zealand University Hospital, Roskilde, Denmark

Background: The rising incidence of kidney cancer is largely driven by incidental detection of small renal masses, meaning more patients are diagnosed at earlier stages where several treatment strategies are relevant. This has intensified concerns about overtreatment, as some small tumours may never become clinically significant. At the same time, newer techniques, such as ablation therapy and robot-assisted surgery are increasingly used despite limited comparative evidence regarding benefits, risks, and long-term outcomes. In addition, patients’ concerns and quality-of-life implications are often insufficiently understood. We are setting up a national prospective cohort study aimed at addressing these evidence gaps in non-metastatic kidney cancer. Methods: Adults (≥18 years) managed in routine care with active surveillance, ablation therapy, partial nephrectomy or radical nephrectomy will be enrolled across all eight Danish urological centres. In total, 5,920 patients will be included over 5 years. Baseline data will include socioeconomic factors, comorbidity, tumour characteristics, renal function, and treatment details. Comparative effectiveness analyses will follow a target trial emulation framework using modern causal inference methods (including propensity score approaches) to enable robust comparisons in a setting where randomised trials have been infeasible. Patient-reported outcomes will be collected via questionnaires (EORTC QLQ-C30 and QoR-15) distributed to participants using the Danish national digital post system, at 1, 3, and 12 months after treatment. Long-term follow-up will be performed at 5, 10, and 15 years after diagnosis and, in addition to patient-reported outcomes, will capture oncological, renal, and cardiovascular outcomes - including recurrence and survival endpoints. Perspective: By combining a nationwide cohort with target trial emulation, patient-reported outcomes, and long-term follow-up, the study will provide robust evidence to guide personalised treatment selection and strengthen patient counselling.



# Abstract: Validation of the IMDC Prognostic Model in Patients With Metastatic Renal Cell Carcinoma Treated With First-Line Tivozanib: TIVOREAL-SOGUG, A Spanish Real-World Experience

**AUTHORS**

Sergio Vázquez, Hospital Universitario Lucus Augusti, Lugo, Spain

María José Méndez-Vidal, Maimonides Institute for Biomedical Research of Córdoba (IMIBIC), Hospital Universitario Reina Sofía (HURS), Medical Oncology Department, Córdoba, Spain

Aránzazu González-del-Alba, Hospital Universitario Puerta de Hierro-Majadahonda, Madrid, Spain

Inmaculada Aparicio, Hospital General Universitario Gregorio Marañón, Madrid, Spain

Laura López, Hospital Universitario de León, León, Spain

Begoña Pérez-Valderrama, Hospital Universitario Virgen del Rocío, Sevilla, Spain

Enrique Gallardo, Hospital Universitari Parc Taulí, Sabadell, Barcelona, Spain

Javier Gavira, Institut Català d'Oncologia, L’Hospitalet, Barcelona, Spain

Guillermo Crespo, Hospital Universitario de Burgos, Burgos, Spain

Cristina Pernaut⼗, Hospital Universitario Lucus Augusti, Lugo, Spain

Lucía Oliva, Hospital Universitario Virgen de la Victoria, Málaga, Spain

Miguel Ángel Climent, Instituto Valenciano de Oncología, Valencia, Spain

Cristina Suárez, Hospital Universitario Vall d'Hebron, Barcelona, Spain

Imanol Martínez, Hospital Universitario Fundación Jiménez Díaz, Madrid, Spain

Victoria Tirado, Hospital General Universitario Gregorio Marañón, Madrid, Spain

Ángel Rodríguez, Hospital Universitario de León, León, Spain

Alejandro José Barroso, Hospital Universitario Virgen del Rocío, Sevilla, Spain

Natalia Fernández, Hospital Universitario Lucus Augusti, Lugo, Spain

Rosa Rodríguez, Maimonides Institute for Biomedical Research of Córdoba (IMIBIC), Hospital Universitario Reina Sofía (HURS), Medical Oncology Department, Córdoba, Spain

José Ángel Arranz, Hospital General Universitario Gregorio Marañón, Madrid, Spain

Background: The rising incidence of kidney cancer is largely driven by incidental detection of small renal masses, meaning more patients are diagnosed at earlier stages where several treatment strategies are relevant. This has intensified concerns about overtreatment, as some small tumours may never become clinically significant. At the same time, newer techniques, such as ablation therapy and robot-assisted surgery are increasingly used despite limited comparative evidence regarding benefits, risks, and long-term outcomes. In addition, patients’ concerns and quality-of-life implications are often insufficiently understood. We are setting up a national prospective cohort study aimed at addressing these evidence gaps in non-metastatic kidney cancer. Methods: Adults (≥18 years) managed in routine care with active surveillance, ablation therapy, partial nephrectomy or radical nephrectomy will be enrolled across all eight Danish urological centres. In total, 5,920 patients will be included over 5 years. Baseline data will include socioeconomic factors, comorbidity, tumour characteristics, renal function, and treatment details. Comparative effectiveness analyses will follow a target trial emulation framework using modern causal inference methods (including propensity score approaches) to enable robust comparisons in a setting where randomised trials have been infeasible. Patient-reported outcomes will be collected via questionnaires (EORTC QLQ-C30 and QoR-15) distributed to participants using the Danish national digital post system, at 1, 3, and 12 months after treatment. Long-term follow-up will be performed at 5, 10, and 15 years after diagnosis and, in addition to patient-reported outcomes, will capture oncological, renal, and cardiovascular outcomes - including recurrence and survival endpoints. Perspective: By combining a nationwide cohort with target trial emulation, patient-reported outcomes, and long-term follow-up, the study will provide robust evidence to guide personalised treatment selection and strengthen patient counselling.



# Abstract: Real-world treatment patterns in patients with recurrent renal cell carcinoma

**AUTHORS**

Shawna R. Calhoun, Merck & Co., Inc., Rahway, NJ, USA

Marley Boyd, TriNetX, LLC, Cambridge, MA, USA

Neil Dhopeshwarkar, TriNetX, LLC, Cambridge, MA, USA

E. Susan Amirian, TriNetX, LLC, Cambridge, MA, USA

Blessing Dube, Merck & Co., Inc., Rahway, NJ, USA

Gautam Jayram, USA Urology Associates, P.C., Nashville, TN, USA

Benjamin Garmezy, Sarah Cannon Research Institute, Nashville, TN, USA

Background: Approximately 15-45% of patients with renal cell carcinoma (RCC) experience recurrence, yet there is limited evidence on optimal management of recurrence post primary resection. This study describes global real-world practices in management of RCC recurrence. Methods: An observational, retrospective cohort study leveraging de-identified EHR data from the TriNetX Dataworks–USA network was conducted, repeating ex-US analyses exploratorily. Adults (≥18 years) with RCC recurrence post-nephrectomy (per a study validated algorithm) between January 2019-September 2024 were included. Patients diagnosed with other primary cancers or recorded systemic therapy within 90 days after initial nephrectomy were excluded. Patients were followed until the earliest occurrence of death, last EHR record, or data cut-off. Results: Approximately 13.8% of 27,136 eligible US patients with RCC had a recurrence. For the 1,201 patients meeting additional inclusion/exclusion criteria applied to recurrence (mean age 63.9 years, 70.6% male, 74.9% white), the median time from initial nephrectomy to recurrence was 512 days. Within 90 days of recurrence, 37.4% initiated systemic treatment, 21.9% had surgery, 8.8% had radiation therapy, and 36.1% had no recorded treatment. First-line systemic therapy regimens most commonly included a VEGF-inhibitor (52.8%), followed by an immunotherapy (43.1%). Results stratified by clear cell (16.2%) and non-clear cell (4.0%) types showed similar post-recurrence treatment trends, though histology data was often missing (79.9%). Ex-US results (n=1,020) showed higher use of systemic therapy (21.6%) over radiation (2.0%). Conclusion: In this open EHR network, systemic therapy was the most common treatment within 90 days post-recurrence. Since approximately one-third of patients have no recorded treatment, surveillance may be commonly utilized, or documentation of early therapy may be missing. Although most recurrent patients appear to receive therapy, many do not, suggesting increased adherence to multi-disciplinary pathways and consideration of adjuvant strategies may be appropriate.

# Abstract: Is there a benefit of upfront cytoreductive nephrectomy in synchronous metastatic RCC in the immunotherapy era? A population-based nationwide register study

**AUTHORS**

Tarik Almdalal, Department of Immunology, Genetics and Pathology, Uppsala University, Uppsala, Sweden; Department of Surgery and Urology, Eskilstuna County Hospital, Eskilstuna, Sweden

Alaa Kheir, Department of Immunology, Genetics and Pathology, Uppsala University, Uppsala, Sweden

Maja Fahlén, Regional Cancer Centre, Stockholm, Sweden

Ulrika Harmenberg, Department of Oncology-Pathology, Karolinska Institutet, Stockholm, Sweden; Department of Pelvic Cancer, Genitourinary Oncology, Karolinska University Hospital, Stockholm, Sweden

Börje Ljungberg, Department of Diagnostics and Intervention, Urology and Andrology, Umeå University, Umeå, Sweden

Magnus Lindskog, Department of Oncology-Pathology, Karolinska Institutet, Stockholm, Sweden; Department of Pelvic Cancer, Genitourinary Oncology, Karolinska University Hospital, Stockholm, Sweden; Department of Immunology, Genetics and Pathology, Uppsala University, Uppsala, Sweden

Background: The role of cytoreductive nephrectomy in synchronous metastatic renal cell carcinoma (M1 mRCC) is unclear in the current era of immune checkpoint inhibitors. Methods: M1 mRCC patients treated with first line ipilimumab-nivolumab (Ipi-Nivo) Jan 2019-June 2023 were identified in The National Swedish Kidney Cancer Register (NSKCR). Overall survival (OS) was compared for upfront cytoreductive nephrectomy (uCN) followed by Ipi-Nivo versus immediate start of Ipi-Nivo, in the whole cohort and in International Metastatic RCC Database Consortium (IMDC) intermediate and poor risk patients separately. A multivariable survival model was constructed using Cox regression. Results: The NSKCR identified 165 patients with M1 disease treated with Ipi-Nivo during the study period. With a minimal follow-up of 30 months, median OS was 26 months. ECOG 2-3 patients had shorter survival compared to ECOG 1-2 patients (HR for death, 2.78; 95% CI, 1.63-4.73; p<0.001) as did IMDC poor risk compared to intermediate risk patients (HR for death, 2.66; 95% CI, 1.68-4.21; p<0.001). uCN followed by Ipi-Nivo was associated with a 44% lower risk of death compared to immediate Ipi-Nivo (HR, 0.56; 95% CI, 0.37-0.84; p=0.005). In multivariable analysis including uCN, IMDC risk group and ECOG performance status, only IMDC poor risk remained independently associated with OS (HR, 2.63; 95% CI, 1,46-4.74; p=0.001). Analysing survival with uCN followed by Ipi-Nivo versus immediate Ipi-Nivo in the respective risk groups showed a significant benefit of uCN in IMDC intermediate risk patients (HR, 0.44; 95% CI 0.21-0.92; p=0.029) with no benefit in IMDC poor risk patients (HR, 1.1; 95% CI, 0.60-2.02; p=0.75). Conclusions: This population-based real-world study indicates that upfront cytoreductive nephrectomy should be considered in IMDC intermediate risk M1 patients with good performance status prior to start of ipililmumab-nivolumab.
